# Lay Conceptions of Happiness: Associations With Reported Well-Being, Personality Traits, and Materialism

**DOI:** 10.3389/fpsyg.2019.02377

**Published:** 2019-10-18

**Authors:** Mohsen Joshanloo

**Affiliations:** Department of Psychology, Keimyung University, Daegu, South Korea

**Keywords:** conceptions of happiness, happiness, well-being, eudaimonism, personality, materialism

## Abstract

Lay conceptions of happiness are beliefs about the nature, value, antecedents, and outcomes of happiness. Happiness research has largely focused on the levels, predictors, and outcomes of happiness, whereas conceptions of happiness have received less attention. This study sought to expand our understanding of these conceptions by examining a relatively large number of them (i.e., eudaimonism, inclusive happiness, externality of happiness, fear of happiness, transformative suffering, fragility of happiness, valuing happiness, and inflexibility of happiness), in samples from Korea and Canada. Five components of well-being (i.e., social well-being, psychological well-being, life satisfaction, positive affect, and negative affect), the Big Five personality traits, materialism, and demographic variables were measured in addition to conceptions of happiness. The results showed that conceptions of happiness predicted various components of well-being over and above personality traits and demographic variables. These conceptions contributed additional variance to the prediction of materialism. The conceptions were largely independent of personality traits, and there were gender and age differences in the conceptions of happiness. The results also suggest that two dimensions of “effortful virtuosity vs. doubtful pursuit” and “malleability vs. stability” are the underlying dimensions along which the conceptions of happiness vary. There were similarities and differences in the results for Korea and Canada. In sum, this study provides a relatively comprehensive and systematic exploration of the conceptions of happiness, their structure, nomological network, and their relevance to well-being research. It is hoped that these results will stimulate more research on lay conceptions of happiness.

## Introduction

Happiness research has largely focused on studying the levels, predictors, and outcomes of happiness. People’s lay understandings of happiness, however, have received relatively less attention. There are, yet, independent lines of research that focus on a single conception of happiness. For example, the distinction between hedonic and eudaimonic conceptions of well-being (Huta and Ryan, 2010; McMahan and Estes, 2011) and incremental and entity theories of well-being (Howell et al., 2016) have been independently investigated in previous research. However, prior research to date has not systematically explored the structure and associates of these conceptions. The present study sought to take initial steps toward this objective by simultaneously studying eight conceptions of happiness.

Psychologists have extensively studied lay beliefs about the self, others, world, future, and similar. For example, the personal construct theory posits that people anticipate events by the personal meanings they place on those events, i.e., constructs, and people’s behavior is partly shaped by their constructs (Winter and Reed, 2016). People’s social cognitions (i.e., interpretations of own and others’ social behaviors) have also been extensively studied (Fiske and Taylor, 2013). Overall, psychological findings indicate that lay beliefs and constructs have real-life consequences and changing them may lead to changes in mood, behavior, or life outcomes. In fact, changing personal beliefs is an important element of many branches of psychotherapy such as schema therapy and cognitive-behavioral therapy (Beck, 2005).

Conceptions of happiness are another group of personal beliefs and constructs that may have far-reaching real-life consequences. Yet, relatively little is known about these conceptions and their influence on people’s daily lives. In this paper, I will broadly define conceptions of happiness as lay beliefs about the nature, value, antecedents, and outcomes of happiness. Here, eight conceptions of happiness were examined in Korea and Canada. These conceptions are listed and defined in Table 1.

**TABLE 1:** The lay conceptions of happiness examined in this article.

**Title**	**Main theme**	**Definition**	**Sample item**
Eudaimonism	Nature of happiness	That well-being consists of meaningful activity, trying to actualize one’s potential, and gaining a rich understanding of the meaning of life rather than happy feelings, enjoyment, and the absence of negative feelings	Having a sense of purpose and direction in life
Inclusive happiness	Nature of happiness	That our happiness depends on the happiness and well-being of other people, animals, and the natural elements	The connection between your personal happiness and that of all human beings on earth
Externality of happiness	Nature of happiness	That one’s level of happiness is out of one’s control and largely depends on external factors	My happiness is controlled by forces outside my control
Fear of happiness	Value and consequences of happiness	That happiness can cause bad things to happen	Having lots of joy and fun causes bad things to happen
Transformative suffering	Nature and consequences of unhappiness/ill-being	That unhappiness has positive and sometimes transformative powers, and can be an ingredient of well-being	Sometimes sadness and suffering can lead us to happiness
Fragility of happiness	Nature of happiness	That happiness is fleeting and may easily turn into less favorable states	Something might happen at any time and we could easily lose our happiness
Valuing happiness	Value of happiness	That obtaining and maintaining happiness is very important	Feeling happy is extremely important to me
Inflexibility of happiness	Nature of happiness	That one’s level of happiness is fixed and unchangeable	Some people are very happy and some aren’t. People can’t really change how happy they are

Prior research shows that conceptions of happiness are associated with actual levels of well-being. For example, fear of happiness, externality of happiness, and fragility of happiness have been found to be negatively associated with subjective well-being (Joshanloo, 2017b, 2018a; Joshanloo et al., 2017). The present study sought to expand these findings by including a larger number of conceptions and outcomes. The study includes five dimensions of well-being as outcome variables (social well-being, psychological well-being, life satisfaction, positive affect, and negative affect). Social and psychological well-being capture eudaimonic and the latter three variables capture hedonic well-being (Joshanloo, 2016). Another outcome variable was materialism (emphasizing the acquisition of material goods and luxuries in its own right and as a pathway to happiness, Goldberg et al., 2003). It was expected that people’s conceptions of happiness would be associated with their materialistic values as, for example, materialism and hedonism have been found to be positively related (Karabati and Cemalcilar, 2010). To establish the incremental validity of the conceptions, the Big Five personality traits and demographic variables were controlled for in some of the analyses. There is evidence to suggest that personality traits are associated with conceptions of happiness. For example, Joshanloo (2018b) found a positive correlation between fragility of happiness and neuroticism. To expand these findings, the Big Five traits were also used as predictors of the conceptions of happiness.

The study sought to answer five main questions: (1) Are conceptions of happiness significant predictors of various dimensions of well-being? (2) Do the Big Five traits predict conceptions of happiness? (3) Do conceptions of happiness predict well-being and materialism over and above personality traits and demographic variables? (4) What are the underlying dimensions along which conceptions of happiness vary? (5) Do conceptions of happiness vary by gender and age? The purpose of the present study was not to statistically compare results from Korea and Canada and thus measurement invariance was not tested. The analyses were run and presented separately for each nation.

## Methods

### Participants

A total sample of 1177 Korean participants (average age = 40.955, *SD* = 12.097) were included in the study (females = 51.1%). A total sample of 660 Canadian participants (average age = 51.733, *SD* = 15.637) were included in the study (females = 62.9%). These samples consist only of participants who passed the three attention checks included in the survey. The participants were recruited through data collection agencies, and were paid for their participation.

### Measures

In Korea, all of the scales were translated from English into Korean by a team of bilinguals, research assistants, and professors. Reliabilities for all measures are reported in Table 2.

**TABLE 2:** Cronbach’s alphas.

	**Korea**	**Canada**
Social well-being	0.813	0.839
Psychological well-being	0.900	0.891
Life satisfaction	0.920	0.911
Negative affect	0.877	0.897
Positive affect	0.924	0.926
Inclusive happiness	0.893	0.883
Externality of happiness	0.802	0.801
Fear of happiness	0.823	0.904
Transformative suffering	0.830	0.819
Fragility of happiness	0.842	0.803
Valuing happiness	0.663	0.729
Inflexibility of happiness	0.686	0.775
Extraversion	0.720	0.804
Agreeableness	0.704	0.771
Conscientiousness	0.732	0.687
Neuroticism	0.645	0.736
Openness	0.746	0.660
Materialism	0.744	0.779

#### Well-Being

The *social and psychological well-being* subscales of the Mental Health Continuum-Short Form (Keyes, 2006) were used to measure social (five items) and psychological (six items) well-being. The items are responded to on a 6-point scale ranging from 0 = *never* to 5 = *every day.* The *satisfaction with life scale* (Diener et al., 1985) was used to measure life satisfaction. Each of the five items is rated on a 7-point scale ranging from 1 = *strongly disagree* to 7 = *strongly agree*. The *negative and positive affect scale* (Mroczek and Kolarz, 1998; Joshanloo, 2017a) was used to measure positive and negative affect. The scale includes six items for negative affect (e.g., nervous) and six items for positive affect (e.g., cheerful). Respondents indicate how much of the time (ranging from 1 = *none of the time* to 5 = *all of the time*) during the past 30 days they felt each of the affective states.

#### Materialism

The parent materialism measure (Goldberg et al., 2003) was used. The scale has eight items (e.g., “I’d rather spend time shopping than doing almost anything else”). A 4-point scale (1 = *disagree a lot* to 4 = *agree a lot*) was used in Canada. A 5-point scale with an additional response option (3 = *neutral*) was used in Korea.

#### Personality

The Big Five traits were measured using the Mini-IPIP (Donnellan et al., 2006), which is a 20-item short form of the 50-item International Personality Item Pool – five-factor model measure. Each personality trait is measured by four items, using a scale ranging from 1 = *strongly disagree* and 5 = *strongly agree*.

#### Conceptions of Happiness

The items of the 5-item *fear of happiness scale* (Joshanloo, 2013; Joshanloo et al., 2014) are rated on a 7-point scale ranging from 1 = *strongly disagree* to 7 = *strongly agree*. The 4-item *externality of happiness scale* (Joshanloo, 2017b), the 4-item *fragility of happiness scale* (Joshanloo et al., 2015), the 5-item *transformative suffering scale* (Joshanloo, 2014), and the 7-item *valuing happiness scale* (Mauss et al., 2011) have a response format identical to that of the fear of happiness scale. The *inflexibility of happiness scale* was developed for this study, based on Dweck’s (1999) measures of implicit theories of intelligence, personality, and morality. Four items were selected and the words intelligence, personality, or morality were replaced by the word happiness. The scale and validity evidence are presented in the Supplementary  Material.

The *eudaimonism and hedonism scale* was developed for the present study. The participants are asked to distribute 100 points among six components of well-being, which are chosen based on the existing empirical and theoretical literature (Ryan and Deci, 2001; Keyes and Annas, 2009; Tiberius and Hall, 2010; Joshanloo, 2016). Three of the components are hedonic and three of them are eudaimonic. Unlike Likert-type rating scales, in this constant-sum scale, a participant’s allocated points to a component affects his or her available number of points for other components. A high eudaimonism score necessarily means a low hedonism score, similarly the reverse. In addition, this scale is likely to reduce the influence of social desirability which is likely to occur for all of the six components (given that all of them are favorable aspects of well-being). The scale is provided in the Supplementary Material, as are statistical analyses in support of its validity. In the present study, only the eudaimonic well-being score is used and the hedonic well-being score is excluded from the analyses. Including both of the variables in multivariate analyses would be inappropriate given a perfect negative correlation between the two. However, any results obtained with the eudaimonic scores can be reversed to obtain the results for the hedonic well-being. For example, a correlation of 0.2 between eudaimonic well-being and another variable means a correlation of −0.2 between hedonic well-being and that variable.

The *inclusive happiness scale* was developed for the present study. Following a relatively large number of measures in psychology [such as the allo-inclusive identity scale by Leary et al. (2008)] that make use of Venn diagrams, this scale presents seven pairs of circles that range from two non-overlapping circles to circles that are nearly congruent. Each item asks about the perceived overlap between one’s happiness and the happiness of a group of people, animals, or plants. The scale and validity evidence for it are presented in the Supplementary  Material.

## Results and Discussion

In all of the regression tables, standardized coefficients are reported. Multicollinearity was not a concern in any of the analyses. The full regression results (including tolerance values, unstandardized regression coefficients, and confidence intervals) are provided in the Supplementary  Material.

### Conceptions of Happiness Predicting Levels of Well-Being

Ten separate regression analyses were conducted, one for each of the five well-being dimensions in each nation, with the eight conceptions of happiness as predictors. The results are reported in Table 3. The conceptions explain between 15.9% (predicting social well-being in Korea) and 30.4% (predicting positive affect in Canada) of the variance in well-being. The conceptions were generally better predictors in Canada than in Korea, and the conceptions were better predictors of hedonic aspects than eudaimonic aspects. Thus, well-being-related beliefs are associated with reported levels of experienced well-being. However, the presence or direction of causality cannot be inferred from the present cross-sectional results.

**TABLE 3:** Conceptions of happiness predicting levels of well-being (standardized regression coefficients).

	**Outcome**									
	**Social well-being**		**Psychological well-being**		**Life satisfaction**		**Negative affect**		**Positive affect**	
**Predictor**	**Korea**	**Canada**	**Korea**	**Canada**	**Korea**	**Canada**	**Korea**	**Canada**	**Korea**	**Canada**
Eudaimonism	0.068^∗^	0.131^∗∗∗^	0.077^∗∗^	0.074^∗^	–0.039	–0.043	0.019	0.012	−0.061^∗^	0.003
Inclusive happiness	0.240^∗∗∗^	0.156^∗∗∗^	0.176^∗∗∗^	0.180^∗∗∗^	0.165^∗∗∗^	0.163^∗∗∗^	–0.048	0.005	0.176^∗∗∗^	0.200^∗∗∗^
Externality of happiness	–0.129^∗∗∗^	–0.080	–0.166^∗∗∗^	–0.132^∗∗^	–0.196^∗∗∗^	–0.171^∗∗∗^	0.240^∗∗∗^	0.160^∗∗∗^	–0.187^∗∗∗^	–0.212^∗∗∗^
Fear of happiness	–0.095^∗∗^	–0.246^∗∗∗^	–0.182^∗∗∗^	–0.268^∗∗∗^	–0.132^∗∗∗^	–0.288^∗∗∗^	0.164^∗∗∗^	0.248^∗∗∗^	–0.181^∗∗∗^	–0.324^∗∗∗^
Transformative suffering	0.116^∗∗∗^	0.077^∗^	0.155^∗∗∗^	0.085^∗^	0.164^∗∗∗^	0.106^∗∗^	–0.048	–0.012	0.174^∗∗∗^	0.097^∗∗^
Fragility of happiness	–0.153^∗∗∗^	–0.146^∗∗∗^	–0.156^∗∗∗^	−0.084^∗^	–0.185^∗∗∗^	–0.114^∗∗^	0.126^∗∗∗^	0.070	–0.156^∗∗∗^	−0.088^∗^
Valuing happiness	0.059^∗^	0.016	0.098^∗∗^	–0.062	–0.050	−0.091^∗^	0.194^∗∗∗^	0.273^∗∗∗^	–0.023	−0.094^∗^
Inflexibility of happiness	–0.018	0.126^∗∗^	0.000	0.062	0.041	0.169^∗∗∗^	−0.066^∗^	–0.162^∗∗∗^	0.021	0.182^∗∗∗^
*R*^2^	0.159	0.178	0.180	0.210	0.179	0.241	0.224	0.288	0.186	0.304

*^∗^*p* < 0.05. ^∗∗^*p* < 0.01. ^∗∗∗^*p* < 0.001.*

### The Big Five Predicting Conceptions of Happiness

Sixteen separate regression analyses were performed, one for each of the eight conceptions in each nation, with the Big Five traits as predictors. As shown in Table 4, the contribution of the personality traits ranged between 1.4% (predicting transformative suffering in Korea) and 19.9% (predicting fear of happiness in Canada). Personality traits were generally better predictors in Canada than in Korea. With an average contribution of about 7% across the nations, it can be concluded that the personality traits and conceptions of happiness are associated, yet not strongly so.

**TABLE 4:** The Big Five predicting conceptions of happiness (standardized regression coefficients).

	**Predictor**											
	**Extraversion**		**Agreeableness**		**Conscientiousness**		**Neuroticism**		**Openness**		***R*^2^**	
**Outcome**	**Korea**	**Canada**	**Korea**	**Canada**	**Korea**	**Canada**	**Korea**	**Canada**	**Korea**	**Canada**	**Korea**	**Canada**
Eudaimonism	0.047	–0.048	–0.052	0.160^∗∗∗^	0.080^∗∗^	–0.019	–0.092^∗∗^	–0.049	0.050	0.184^∗∗∗^	0.025	0.072
Inclusive happiness	0.020	0.149^∗∗∗^	0.093^∗∗^	0.102^∗^	0.015	–0.064	–0.087^∗∗^	0.021	0.022	0.103^∗∗^	0.022	0.058
Externality of happiness	–0.053	–0.169^∗∗∗^	−0.077^∗^	–0.131^∗∗^	–0.031	−0.090^∗^	0.230^∗∗∗^	0.170^∗∗∗^	−0.068^∗^	–0.067	0.083	0.154
Fear of happiness	–0.032	–0.126^∗∗^	–0.106^∗∗^	–0.190^∗∗∗^	–0.055	–0.111^∗∗^	0.141^∗∗∗^	0.232^∗∗∗^	–0.048	–0.021	0.052	0.199
Transformative suffering	–0.020	–0.008	0.097^∗∗^	0.127^∗∗^	0.058	–0.123^∗∗^	0.055	0.055	–0.048	0.044	0.014	0.032
Fragility of happiness	–0.104^∗∗^	–0.142^∗∗∗^	0.026	0.008	0.034	–0.011	0.158^∗∗∗^	0.250^∗∗∗^	0.037	–0.043	0.032	0.108
Valuing happiness	0.019	0.037	–0.012	–0.069	0.080^∗∗^	–0.062	0.265^∗∗∗^	0.343^∗∗∗^	–0.028	–0.049	0.069	0.151
Inflexibility of happiness	–0.018	–0.012	–0.133^∗∗∗^	–0.216^∗∗∗^	–0.017	–0.078	–0.008	−0.106^∗^	–0.010	–0.070	0.022	0.071

*^∗^*p* < 0.05. ^∗∗^*p* < 0.01. ^∗∗∗^*p* < 0.001.*

### Incremental Contribution of Conceptions of Happiness to Experienced Well-Being

Ten separate hierarchical regression analyses were performed, one for each component of well-being in each nation. In Step 1, age, gender, and the Big Five were entered, and in Step 2, the conceptions were entered. As shown in Table 5, the unique contribution of the conceptions ranged between 3.5% (predicting psychological well-being in Canada) and 11% (predicting negative affect in Korea), suggesting that the conceptions contributed a significant amount of variance over and above the demographic and personality characteristics.

**TABLE 5:** Conceptions of happiness predicting levels of well-being and materialism over and above age, gender, and the Big Five (standardized regression coefficients).

	**Outcome**											
	**Social well-being**		**Psychological well-being**		**Life satisfaction**		**Negative affect**		**Positive affect**		**Materialism**	
**Predictor**	**Korea**	**Canada**	**Korea**	**Canada**	**Korea**	**Canada**	**Korea**	**Canada**	**Korea**	**Canada**	**Korea**	**Canada**
Age	0.011	0.054	–0.021	0.038	–0.094^∗∗^	0.026	–0.083^∗∗^	–0.147^∗∗∗^	–0.019	–0.015	–0.236^∗∗∗^	–0.291^∗∗∗^
Male	0.069^∗∗^	0.059	–0.034	0.038	–0.053^∗∗^	–0.033	0.034	–0.096^∗∗∗^	–0.016	0.023	–0.023	–0.010
Extraversion	0.214^∗∗∗^	0.118^∗∗^	0.233^∗∗∗^	0.173^∗∗∗^	0.173^∗∗∗^	0.067^∗^	–0.090^∗∗^	−0.059^∗^	0.250^∗∗∗^	0.103^∗∗^	0.053	0.068
Agreeableness	0.081^∗∗^	0.140^∗∗∗^	0.052	0.152^∗∗∗^	0.031	0.082^∗^	0.022	–0.032	–0.017	0.067^∗^	–0.003	–0.074
Conscientiousness	0.030	–0.004	0.132^∗∗∗^	0.093^∗∗^	0.097^∗∗∗^	0.047	–0.073^∗∗^	–0.114^∗∗∗^	0.044	0.084^∗∗^	0.050	0.014
Neuroticism	–0.156^∗∗∗^	–0.251^∗∗∗^	–0.221^∗∗∗^	–0.292^∗∗∗^	–0.174^∗∗∗^	–0.403^∗∗∗^	0.345^∗∗∗^	0.466^∗∗∗^	–0.192^∗∗∗^	–0.472^∗∗∗^	0.079^∗∗^	–0.009
Openness	0.061^∗^	0.064	0.160^∗∗∗^	0.042	0.044	–0.037	–0.003	0.067^∗^	0.076^∗∗^	–0.050	–0.010	–0.038
Eudaimonism	0.042	0.098^∗∗^	0.035	0.041	−0.054^∗^	–0.057	0.070^∗∗^	0.014	–0.091^∗∗∗^	–0.009	–0.111^∗∗∗^	−0.072^∗^
Inclusive happiness	0.212^∗∗∗^	0.105^∗∗^	0.132^∗∗∗^	0.117^∗∗∗^	0.140^∗∗∗^	0.127^∗∗∗^	–0.010	0.019	0.147^∗∗∗^	0.166^∗∗∗^	–0.010	0.023
Externality of happiness	−0.069^∗^	–0.033	–0.094^∗∗^	–0.063	–0.156^∗∗∗^	–0.119^∗∗^	0.165^∗∗∗^	0.114^∗∗^	–0.132^∗∗∗^	–0.146^∗∗∗^	0.158^∗∗∗^	0.072
Fear of happiness	–0.061	–0.136^∗∗^	–0.117^∗∗∗^	–0.121^∗∗^	–0.086^∗∗^	–0.154^∗∗∗^	0.135^∗∗∗^	0.101^∗∗^	–0.144^∗∗∗^	–0.176^∗∗∗^	−0.077^∗^	–0.010
Transformative suffering	0.098^∗∗^	0.042	0.137^∗∗∗^	0.046	0.154^∗∗∗^	0.071^∗^	–0.034	–0.004	0.165^∗∗∗^	0.058	–0.054	0.015
Fragility of happiness	–0.125^∗∗∗^	–0.102^∗∗^	–0.135^∗∗∗^	–0.031	–0.172^∗∗∗^	–0.062	0.089^∗∗∗^	0.010	–0.130^∗∗∗^	–0.023	0.047	0.062
Valuing happiness	0.063^∗^	0.099^∗^	0.119^∗∗∗^	0.036	–0.023	0.023	0.141^∗∗∗^	0.118^∗∗∗^	–0.003	0.033	0.369^∗∗∗^	0.292^∗∗∗^
Inflexibility of happiness	–0.017	0.081^∗^	–0.001	0.008	0.048	0.093^∗^	–0.026	–0.039	0.016	0.087^∗∗^	0.017	0.061
Demographics and personality’s *R*^2^	0.179	0.243	0.286	0.360	0.162	0.343	0.251	0.543	0.186	0.460	0.114	0.156
Conceptions’ incremental *R*^2^	0.090	0.057	0.085	0.035	0.107	0.059	0.110	0.050	0.108	0.074	0.184	0.124

*^∗^*p* < 0.05. ^∗∗^*p* < 0.01. ^∗∗∗^*p* < 0.001.*

### Incremental Contribution of Conceptions of Happiness to Materialism

As shown in Table 5, the unique contributions of the conceptions beyond personality and demographic variables were about 18% in Korea, and 12% in Canada. In Korea, the unique contribution of the conceptions was larger than the collective contribution of personality and demographic variables (about 11%). The strongest predictors of materialism were found to be age and valuing happiness.

### Exploring the Structure of the Conceptions of Happiness

**FIGURE 1:**
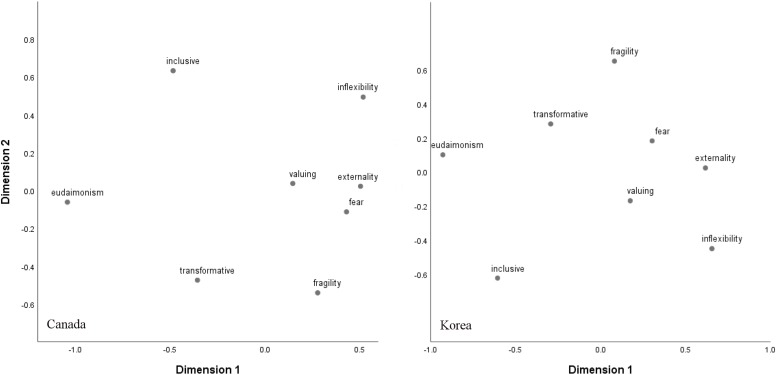
Multidimensional scaling plots of conceptions of happiness.

Multidimensional scaling was used to explore the underlying dimensions in the eight conceptions of happiness. The data were analyzed by means of metric PROXSCAL (Commandeur and Heiser, 1993) in SPSS 25. In compliance with the common recommendations for best practice (Davison et al., 2010; Bilsky et al., 2011; Borg et al., 2013), the analysis was based on squared Euclidean distances, and Z transformation, with a Torgerson initial configuration. The analysis was run separately in each nation. The resulting two-dimensional plots are presented in Figure 1 (Stress-1 = 0.144 and 0.124 in Korea and Canada, respectively). As can be seen, despite the point by point differences between the nations, the general structure of the conceptions is similar. Eudaimonism, transformative suffering, and inclusiveness clustered at the left side of the horizontal axis. Central to this cluster of variables is an emphasis on personal and social virtues (rather than happy feelings), transcending personal interests, and purpose in life. Thus the three variables tap into a very broad concept that can be titled “effortful virtuosity,” which is one way to transcend the active pursuit of or obsession with emotional happiness. On the other hand, externality, inflexibility, fear, valuing, and fragility form the opposite side of this dimension. These variables collectively indicate a hesitation about the value of happiness, doubt about its achievability, but valuing it anyway. Thus the cluster may be broadly titled “doubtful pursuit.” The variables constructing effortful virtuosity are generally positively associated with well-being, whereas the variables that make up doubtful pursuit are generally negatively associated with well-being (Table 3).

One end of the vertical dimension is occupied by fragility and transformative suffering, whereas the other end is occupied by inflexibility and inclusive happiness. What conceptually connects fragility and transformative suffering is their emphasis on change. Fragility of happiness emphasizes the notion that happiness can be altered, and transformative suffering emphasizes the notion that unhappiness can be altered. Thus both of the factors are about the possibility of change, and the cluster may be titled “malleability.” Inflexibility and inclusive happiness, on the other hand, are more about stability. This point is perhaps more obvious for inflexibility of happiness. Yet a central theme to inclusive happiness is the notion that our happiness depends on things other than ourselves, and thus changing our level of happiness would be a collective project which requires the involvement of others. In other words, changing one’s level of happiness would depend on the broader context of one’s actual and symbolic relationships with the non-self. In essence, inclusive happiness also implies the difficulty of changing personal happiness. Therefore, this cluster may be titled “stability.” It should also be noted that the latter cluster also represents the highly social variable of inclusive happiness as opposed to the malleability cluster which emphasizes personal control over happiness, and thus another underlying theme of this axis is social vs. personal.

### Relationships With Age and Gender

The correlations between age and conceptions are shown in Supplementary Table S5. The strongest association was −0.175 (between age and valuing in Canada), suggesting that age is not a strong predictor of conceptions, yet the associations are not all trivial. Notably, there are cultural differences in the age differences. For example, transformative suffering is positively correlated with age in Korea, and negatively correlated with age in Canada. Age was a positive correlate of valuing in Korea and a negative correlate in Canada. That conceptions of happiness vary by age is consistent with previous research. For example, Mogilner et al. (2011) report that younger people are more likely to associate happiness with excitement, and older people are more likely to associate happiness with peacefulness. Carlquist et al. (2017) found that older participants were more likely to include external life domains in their descriptions of well-being. Although longitudinal studies are needed for firmer conclusions, these findings do suggest that conceptions of happiness are contingent on one’s developmental stage. The present findings highlight the fact that the correlations between age and conceptions are culturally variable, and generalizing from one culture to the other is not warranted without additional analysis.

Sixteen separate *t* tests were performed to examine gender differences in the eight conceptions in each country. The results for significant gender differences (two in Korea and four in Canada) are shown in Supplementary Table S6. As shown, the effect sizes are small to moderate and the only culturally consistent gender difference is fear of happiness on which males scored higher than females in both cultures. Whereas some previous studies have not found any gender differences in conceptions of happiness (e.g., Tafarodi et al., 2012; Carlquist et al., 2017), others have revealed gender differences. For example, Furnham and Cheng (2000) found that females believed more that social support was an important cause of happiness. The present study also suggest that conceptions vary according to gender. Again, the present findings highlight the importance of culture by showing that the relationships between conceptions of happiness and gender are variable across the two countries.

## Conclusion

This study was the first systematic analysis of conceptions of happiness, their underlying structure, and their associations with personality, demographic variables, levels of well-being, and materialistic values. The results suggest that the two dimensions of effortful virtuosity vs. doubtful pursuit, and malleability vs. stability, can be inferred as two underlying dimensions along which the conceptions of happiness vary. The results also suggest that conceptions of happiness are largely independent of personality, they are associated with hedonic and eudaimonic aspects of well-being and materialism, and they contribute additional variance to the prediction of the outcomes over and above personality and demographic variables. There are cultural similarities and differences in the results, and thus findings from a single culture cannot be generalized to other cultures without further analysis.

Caution should be used in interpreting these results, however. Three of the scales were developed for this study, and their validity needs to be further investigated in Korea, Canada, and other countries. Findings will need to be replicated in more countries and cultures, with more diverse variables, and using longer scales of personality and eudaimonic well-being. Moreover, the present study inevitably examined a limited number of conceptions of happiness. There are other beliefs surrounding happiness that deserve research attention. For example, some cultures and individuals seem to differ on their views of how spiritual or material happiness is, or some distinguish between earthly and afterlife versions of happiness (Joshanloo, 2014). An interesting avenue for future research is to empirically examine these and other conceptions of happiness. Despite these limitations and the exploratory nature of the findings at this stage, it is hoped that the present study will stimulate further empirical research in this area. Diversification of the methods in this line of research by conducting more longitudinal (including diary studies) and experimental studies would be a necessary next step.

## Data Availability Statement

The datasets generated for this study are available on request to the corresponding author.

## Ethics Statement

The studies involving human participants were reviewed and approved by the Keimyung University IRB. The patients/participants provided their written informed consent to participate in this study.

## Author Contributions

The author confirms being the sole contributor of this work and has approved it for publication.

## Conflict of Interest

The authors declare that the research was conducted in the absence of any commercial or financial relationships that could be construed as a potential conflict of interest.
