# Supplementary material for: Lay Conceptions of Happiness: Associations With Reported Well-Being, Personality Traits, and Materialism
Source: Front Psychol. 2019 Oct 18;10:2377. doi: 10.3389/fpsyg.2019.02377 (PMC6813919; doi:10.3389/fpsyg.2019.02377)
Supplement: Supplementary file 1 [file Data_Sheet_1.docx]

**The Inflexibility of Happiness Scale**

Please rate the extent to which you agree with each of the following statements.

| 1 | 2 | 3 | 4 | 5 | 6 | 7 |
| --- | --- | --- | --- | --- | --- | --- |
| Strongly disagree | Somewhat disagree | A little disagree | Neither Agree or Disagree | A little agree | Somewhat agree | Strongly agree |

| 1. A person’s level of happiness is something very basic about them, and it can’t be changed much. |
| --- |
| 1. Whether a person is happy or not is deeply ingrained in their personality. It cannot be changed very much. |
| 1. Some people are very happy and some aren’t. People can’t really change how happy they are. |
| 1. No matter who somebody is, they can always change how happy a person they are. |

Separate exploratory factor analyses (principal axis factoring) were conducted in each nation. Scree tests indicated that the optimal number of factor is one in both of the nations. Factor loadings for the single-factor structure and Cronbach’s alphas are reported in Table S1. Together, these results support the one-factor structure of the scale and its acceptable reliability in both nations. This scale was expected to have a positive correlation with externality of happiness, given the two concepts’ emphasis on the lack of control over one’s happiness. As shown in Table S4, the two scales correlated moderately and positively in both nations, which indicates acceptable convergent validity. It is noteworthy that Howell, Passmore, and Holder (2016) have also developed a similar scale based on Dweck’s scales, titled the Implicit Theories of Well-Being Scale. Their scale is longer (eight items) and they use the word well-being rather than happiness in their items.

| Table S1  *Factor Loadings and Alphas for the Inflexibility of Happiness Scale* | | |
| --- | --- | --- |
|  | Korea | Canada |
| 1. A person’s level of happiness is something very basic about them, and it can’t be changed much. | .552 | .727 |
| 1. Whether a person is happy or not is deeply ingrained in their personality. It cannot be changed very much. | .764 | .886 |
| 1. Some people are very happy and some aren’t. People can’t really change how happy they are. | .743 | .776 |
| 1. No matter who somebody is, they can always change how happy a person they are (reverse-coded). | .330 | .356 |
| Eigenvalue | 2.079 | 2.434 |
| % of variance explained | 51.983 | 60.859 |
| Α | .686 | .775 |

**The Inclusive Happiness Scale**

Below are seven diagrams that express varying degree of relatedness or connection between two things. For example, Diagram 1 indicates no relationship or connectedness, Diagram 4 indicates a moderate degree of connectedness, and Diagram 7 indicates complete connectedness. For each of the four items below, please specify which diagram best shows the relatedness between the two things.


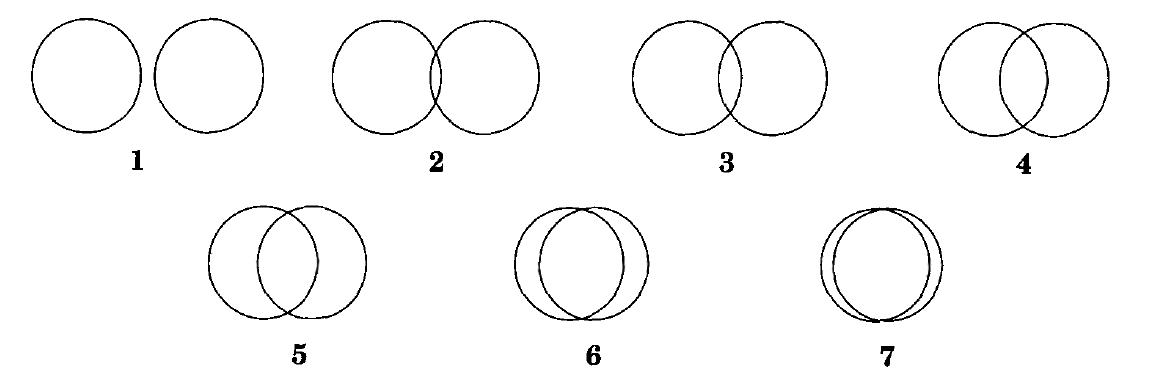


| 7 | 6 | 5 | 4 | 3 | 2 | 1 | 1. The connection between your personal happiness and that of your friends. |
| --- | --- | --- | --- | --- | --- | --- | --- |
| 7 | 6 | 5 | 4 | 3 | 2 | 1 | 1. The connection between your personal happiness and that of all human beings on earth. |
| 7 | 6 | 5 | 4 | 3 | 2 | 1 | 1. The connection between your personal happiness and that of your country. |
| 7 | 6 | 5 | 4 | 3 | 2 | 1 | 1. The connection between your personal happiness and that of the Earth. |
| 7 | 6 | 5 | 4 | 3 | 2 | 1 | 1. The connection between your personal happiness and that of a wild animal (such as a squirrel, rabbit, deer, or wolf). |
| 7 | 6 | 5 | 4 | 3 | 2 | 1 | 1. The connection between your personal happiness and that of all living creatures. |
| 7 | 6 | 5 | 4 | 3 | 2 | 1 | 1. The connection between your personal happiness and that of a tree. |

Separate exploratory factor analyses (principal axis factoring) in Korea and Canada were performed. Screes test indicated that the optimal number of factors would be one in both nations. The factors loadings of the one-factor structure and alphas are reported in Table S2. Together, these results support the one-factor structure of the scale and its acceptable reliability in both nations. This variable was expected to be positively associated with agreeableness, given the two variables’ social emphasis. The results showed that the two variables were positively correlated (.112 and .151, in Korea and Canada respectively, *p*s < .001).

It is noteworthy that one item, “The connection between your personal happiness and that of your family,” was removed from the scale due to its low loading (.209) on the inclusive happiness factor in Korea. The items had a relatively high kurtosis (2.163) and skewness (-1.362) in Korea and required a separate factor of itself. This indicates that in family oriented cultures such as Korea (Shin, Suh, Eom, & Kim, 2018), the majority will respond with very high ratings for this item and thus, the item is expected to be a source of cross-cultural non-invariance. In consequence, I decided to remove the item.

| Table S2  *Factor Loadings and Alphas for the Inclusive Happiness Scale* | | |
| --- | --- | --- |
|  | Korea | Canada |
| 1. The connection between your personal happiness and that of your friends. | .465 | .462 |
| 1. The connection between your personal happiness and that of all human beings on earth. | .753 | .720 |
| 1. The connection between your personal happiness and that of your country. | .698 | .708 |
| 1. The connection between your personal happiness and that of the Earth. | .821 | .818 |
| 1. The connection between your personal happiness and that of a wild animal (such as a squirrel, rabbit, deer, or wolf). | .799 | .785 |
| 1. The connection between your personal happiness and that of all living creatures. | .816 | .834 |
| 1. The connection between your personal happiness and that of a tree. | .827 | .721 |
| Eigenvalue | 4.331 | 4.163 |
| % of variance explained | 61.868 | 59.476 |
| Α | .893 | .883 |

**The Eudaimonism-Hedonism Scale**

Listed below are 6 factors that can be considered as components of well-being. Please let us know about your personal definition of well-being by distributing points to these 6 components. You have a total of 100 points that should be distributed based on the importance of each component in your personal opinion. Note that the total points you allocate to all components must be exactly 100. Please read the entire list of components before you begin allocating points.

| **Components of well-being** | | **Points** |
| --- | --- | --- |
| 1- Experiencing happy feelings | | …………. |
| 2- Having a sense of purpose and direction in life | | …………. |
| 3- Enjoying oneself | | …………. |
| 4- Trying to actualize one’s potential and talents | | …………. |
| 5- Gaining a rich understanding of the meaning of life | | …………. |
| 6- Absence of negative feelings | | …………. |
|  | Total: | …………. |

- PLEASE CHECK AGAIN: Total points allocated should not exceed 100.

Scoring: Hedonism: Items 1, 3, and 6; Eudaimonism: items 2, 4, and 5

Factor analysis may not be used to evaluate this measure because of the interdependence of the items on each other. In essence, all items are in competition with each other, and thus negative correlations are to be expected between all items. Yet, it was expected that any eudaimonic item would have weaker negative correlations with the other eudaimonic items than the hedonic items (and any hedonic item would have weaker negative correlations with hedonic than eudaimonic items). That is to say, eudaimonic items are expected to have weaker negative correlations between themselves and stronger negative correlations with hedonic items. The correlation matrix is shown in Table S3. The average correlation between the hedonic items was -0.131, and between eudaimonic items was 0.004. The average correlation between hedonic and eudaimonic items was -.280. This also suggests that eudaimonic items are more likely to be collectively given high or low points, than hedonic items that are more likely to compete with each other. This analysis was supplemented by multidimensional scaling (PROXSCAL, derived from the Torgerson starting configuration, with z-standardized variables). The two-dimensional plots for both of the nations are presented in Figure S1. As can be seen, the hedonic and eudaimonic items formed two separate clusters in each of the two nations. Figure S2 shows the distribution of points in the nations. Eudaimonism was expected to have a stronger relationship with the eudaimonic components of well-being (social and psychological well-being), than the hedonic components (life satisfaction and affect). This prediction was supported, which attests to the convergent validity of the scale. As shown in Table 3, eudaimonism was a better predictor of eudaimonic than hedonic well-being (which indicates, conversely, that hedonism was a better predictor of hedonic than eudaimonic well-being).

It is noteworthy that the word well-being was used in the instructions of the scale rather than happiness, which is a more common and familiar word for the participants. The reason for this is that using the word happiness may bias the participants towards weighting the hedonic options more than eudaimonic ones. For example, participants may give higher points to the option “experiencing happy feelings” if the word happiness is used in the instructions.

| Table S3  *Intercorrelations Between the Items of the Eudaimonism-Hedonism Scales* | | | | | | |
| --- | --- | --- | --- | --- | --- | --- |
|  | 1 | 2 | 3 | 4 | 5 | 6 |
| 1. Experiencing happy feelings | 1 |  |  |  |  |  |
| 1. Enjoying oneself | -.034 | 1 |  |  |  |  |
| 1. Absence of negative feelings | -.221^**^ | -.139^**^ | 1 |  |  |  |
| 1. Having a sense of purpose and direction in life | -.356^**^ | -.343^**^ | -.246^**^ | 1 |  |  |
| 1. Trying to actualize one’s potential and talents | -.390^**^ | -.220^**^ | -.170^**^ | -.018 | 1 |  |
| 1. Gaining a rich understanding of the meaning of life | -.405^**^ | -.307^**^ | -.085^**^ | -.007 | .038 | 1 |
| ^**^ Significant at .01 | | | | | | |


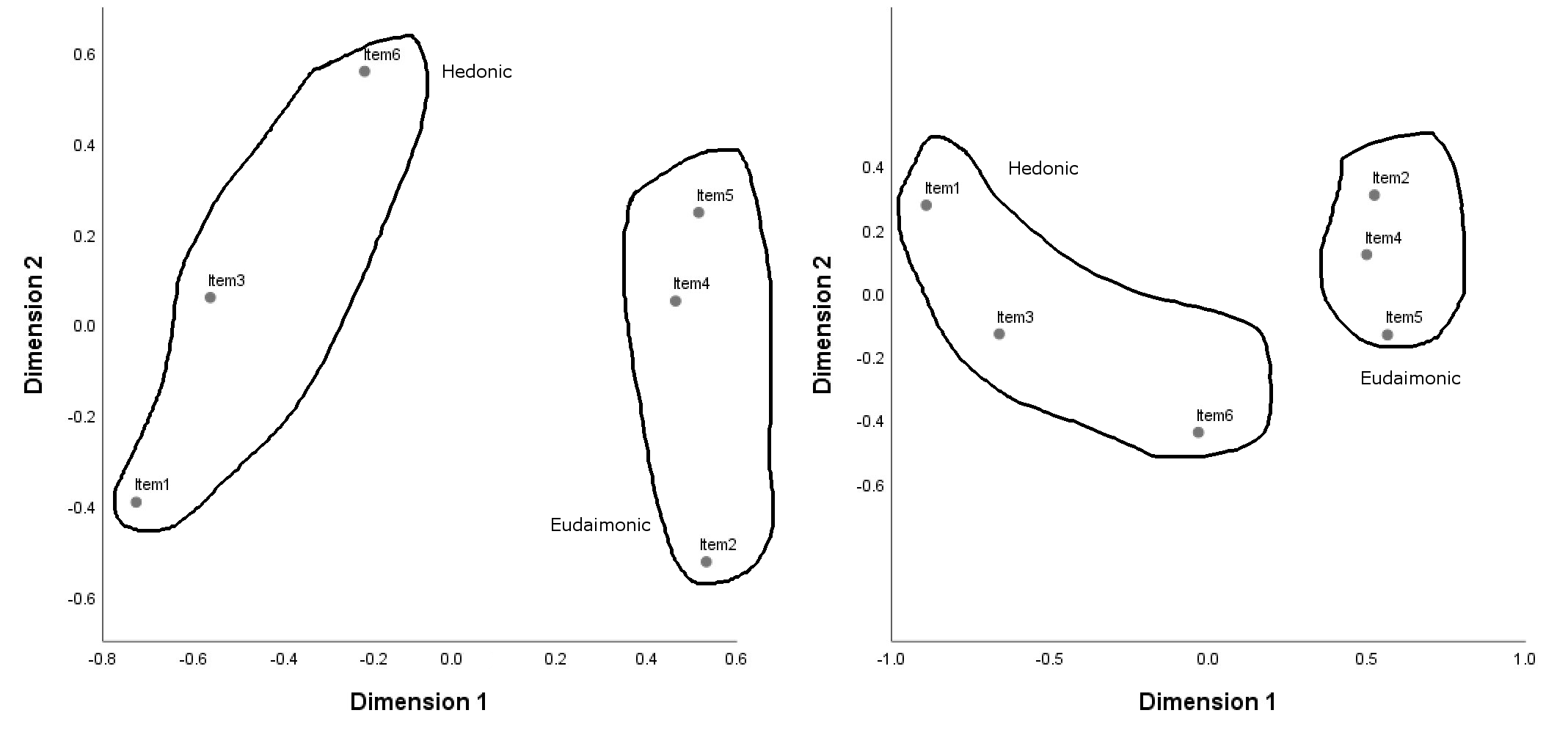


Figure S1

Multidirectional scaling plots for Canada (left) and Korea (right)


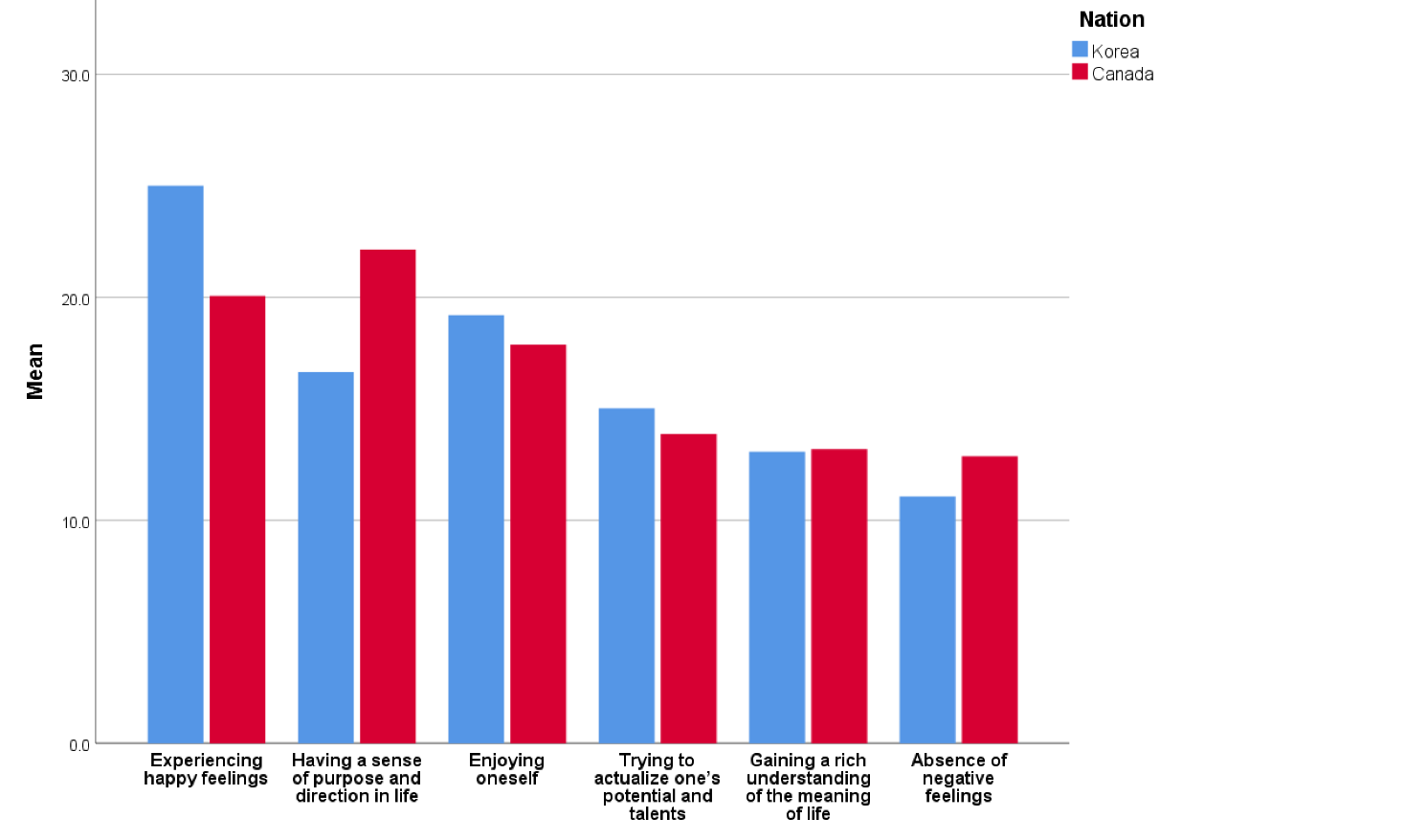


Figure S2

Points given to the six well-being components across nations

| Table S4  *Intercorrelations Between the Conceptions of Happiness* | | | | | | | | |
| --- | --- | --- | --- | --- | --- | --- | --- | --- |
|  |  | 1 | 2 | 3 | 4 | 5 | 6 | 7 |
| Korea | |  |  |  |  |  |  |  |
|  | 1. Eudaimonism | 1 |  |  |  |  |  |  |
|  | 1. Inclusive happiness | .114^***^ | 1 |  |  |  |  |  |
|  | 1. Externality of happiness | -.090^**^ | -.085^**^ | 1 |  |  |  |  |
|  | 1. Fear of happiness | .034 | -.004 | .446^***^ | 1 |  |  |  |
|  | 1. Transformative suffering | .150^***^ | .177^***^ | .008 | .331^***^ | 1 |  |  |
|  | 1. Fragility of happiness | -.006 | -.079^**^ | .158^***^ | .223^***^ | .286^***^ | 1 |  |
|  | 1. Valuing happiness | -.051 | .056 | .277^***^ | .271^***^ | .187^***^ | .186^***^ | 1 |
|  | 1. Inflexibility of happiness | -.090^**^ | -.020 | .334^***^ | .183^***^ | .001 | .012 | .135^***^ |
| Canada | |  |  |  |  |  |  |  |
|  | 1. Eudaimonism | 1 |  |  |  |  |  |  |
|  | 1. Inclusive happiness | .089^*^ | 1 |  |  |  |  |  |
|  | 1. Externality of happiness | -.111^**^ | .009 | 1 |  |  |  |  |
|  | 1. Fear of happiness | -.065 | -.008 | .573^***^ | 1 |  |  |  |
|  | 1. Transformative suffering | .137^***^ | .140^***^ | .089^*^ | .255^***^ | 1 |  |  |
|  | 1. Fragility of happiness | -.054 | -.055 | .334^***^ | .375^***^ | .254^***^ | 1 |  |
|  | 1. Valuing happiness | -.089^*^ | .189^***^ | .411^***^ | .383^***^ | .166^***^ | .310^***^ | 1 |
|  | 1. Inflexibility of happiness | -.086^*^ | .084^*^ | .438^***^ | .341^***^ | -.025 | .049 | .174^***^ |
|  | | | | | | | | |

| Table S5  *The Relationship Between age and Conceptions of Happiness* | | | | | | | | |
| --- | --- | --- | --- | --- | --- | --- | --- | --- |
|  | Eudaimonism | Inclusive | Externality | Fear | Transformative | Fragility | Valuing | Inflexibility |
| Korea | .165^***^ | .116^***^ | -.044 | .020 | .112^***^ | -.059^*^ | .065^*^ | .072^*^ |
| Canada | -.029 | -.023 | -.032 | -.125^**^ | -.156^***^ | -.087^*^ | -.175^***^ | .080^*^ |
|  | | | | | | | | |

| Table S6  *Significate Gender Differences in the Conceptions of Happiness* | | | | | | | | |
| --- | --- | --- | --- | --- | --- | --- | --- | --- |
|  | | *t* | *df* | *p* | 95% Confidence Interval  of the Difference | | Cohen’s *d* | Which gender  scored higher? |
|  | |  |  |  | Lower | Upper |  |  |
| Korea | |  |  |  |  |  |  |  |
|  | Fear | 2.571 | 1175 | .010 | .03817 | .28392 | 0.150 | Male |
|  | Valuing | 2.591 | 1175 | .010 | .02612 | .18917 | 0.151 | Male |
| Canada | |  |  |  |  |  |  |  |
|  | Inclusive | -2.835 | 658 | .005 | -.50716 | -.09213 | 0.229 | Female |
|  | Externality | 2.929 | 658 | .004 | .10253 | .51941 | 0.237 | Male |
|  | Fear | 3.001 | 658 | .003 | .11578 | .55393 | 0.239 | Male |
|  | Inflexibility | 3.222 | 658 | .001 | .12640 | .52079 | 0.257 | Male |
|  | | | | | | | | |

**References for the supplementary material**

Shin, J.-e., Suh, E. M., Eom, K., & Kim, H. S. (2018). What Does “Happiness” Prompt in Your Mind? Culture, Word Choice, and Experienced Happiness. [journal article]. *Journal of Happiness Studies, 19*(3), 649-662.

Howell, A. J., Passmore, H. A., & Holder, M. D. (2016). Implicit theories of well-being predict well-being and the endorsement of therapeutic lifestyle changes. *Journal of Happiness Studies*, *17*(6), 2347-2363.
