# Supplementary material for: Lay Conceptions of Happiness: Associations With Reported Well-Being, Personality Traits, and Materialism
Source: Front Psychol. 2019 Oct 18;10:2377. doi: 10.3389/fpsyg.2019.02377 (PMC6813919; doi:10.3389/fpsyg.2019.02377)
Supplement: Supplementary file 2 [file Data_Sheet_2.pdf]

```
REGRESSION
  /MISSING LISTWISE
  /STATISTICS COEFF OUTS CI(95) R ANOVA COLLIN TOL ZPP
  /CRITERIA=PIN(.05) POUT(.10)
  /NOORIGIN
  /DEPENDENT social_well_being
  /METHOD=ENTER eudaimonism inclusive externality fear transformative fragi
lity valuing
    inflexibility.
```

Regression

Notes

Output Created		20-MAY-2019 12:20:00
Comments		
Input	Data	C:\Users\1\Desktop\korea cnada study\merge korea canada\final.sav
	Active Dataset	DataSet1
	Filter	<none>
	Weight	<none>
	Split File	Nation
	N of Rows in Working Data File	1837
Missing Value Handling	Definition of Missing	User-defined missing values are treated as missing.
	Cases Used	Statistics are based on cases with no missing values for any variable used.
Syntax		REGRESSION /MISSING LISTWISE /STATISTICS COEFF OUTS CI(95) R ANOVA COLLIN TOL ZPP /CRITERIA=PIN(.05) POUT(.10) /NOORIGIN /DEPENDENT social_well_being /METHOD=ENTER eudaimonism inclusive externality fear transformative fragility valuing inflexibility.

Notes

Resources	Processor Time	00:00:00.05
	Elapsed Time	00:00:00.19
	Memory Required	13728 bytes
	Additional Memory Required for Residual Plots	0 bytes

Variables Entered/Removed[a]

Nation	Model	Variables Entered	Variables Removed	Method
Korea	1	inflexibility, transformative, eudaimonism, inclusive, valuing, fragility, externality, fear[b]	.	Enter
Canada	1	inflexibility, transformative, eudaimonism, inclusive, fragility, valuing, fear, externality[b]	.	Enter

a. Dependent Variable: social_well_being

b. All requested variables entered.

Model Summary

Nation	Model	R	R Square	Adjusted R Square	Std. Error of the Estimate
Korea	1	.399[a]	.159	.153	.87196
Canada	1	.422[b]	.178	.168	1.04754

a. Predictors: (Constant), inflexibility, transformative, eudaimonism, inclusive, valuing, fragility, externality, fear

b. Predictors: (Constant), inflexibility, transformative, eudaimonism, inclusive, fragility, valuing, fear, externality

ANOVA[a]

Nation	Model		Sum of Squares	df	Mean Square	F	Sig.
Korea	1	Regression	167.862	8	20.983	27.597	.000[b]
		Residual	888.046	1168	.760		
		Total	1055.907	1176			
Canada	1	Regression	154.979	8	19.372	17.654	.000[c]
		Residual	714.372	651	1.097		
		Total	869.352	659			

a. Dependent Variable: social_well_being

b. Predictors: (Constant), inflexibility, transformative, eudaimonism, inclusive, valuing, fragility, externality, fear

c. Predictors: (Constant), inflexibility, transformative, eudaimonism, inclusive, fragility, valuing, fear, externality

Coefficients[a]

Nation	Model		Unstandardized Coefficients		Standardized Coefficients	t
			B	Std. Error	Beta	
Korea	1	(Constant)	1.420	.230		6.174
		eudaimonism	.012	.005	.068	2.490
		inclusive	.202	.023	.240	8.658
		externality	-.115	.029	-.129	-3.979
		fear	-.084	.029	-.095	-2.932
		transformative	.097	.026	.116	3.774
		fragility	-.137	.026	-.153	-5.303
		valuing	.078	.038	.059	2.024
		inflexibility	-.016	.025	-.018	-.624
Canada	1	(Constant)	1.397	.277		5.038
		eudaimonism	.028	.008	.131	3.594
		inclusive	.136	.032	.156	4.189
		externality	-.069	.042	-.080	-1.661
		fear	-.203	.039	-.246	-5.259
		transformative	.070	.035	.077	1.993
		fragility	-.129	.036	-.146	-3.589
		valuing	.018	.048	.016	.377
		inflexibility	.116	.037	.126	3.112

Coefficients[a]

Nation	Model		Sig.	95.0% Confidence Interval for B Lower Bound	Upper Bound	Correlations Zero-order
Korea	1	(Constant)	.000	.968	1.871	
		eudaimonism	.013	.003	.022	.121
		inclusive	.000	.156	.248	.296
		externality	.000	-.172	-.058	-.211
		fear	.003	-.140	-.028	-.135
		transformative	.000	.046	.147	.103
		fragility	.000	-.187	-.086	-.170
		valuing	.043	.002	.153	-.002
		inflexibility	.533	-.066	.034	-.083
Canada	1	(Constant)	.000	.853	1.942	
		eudaimonism	.000	.013	.043	.176
		inclusive	.000	.072	.200	.202
		externality	.097	-.151	.013	-.214
		fear	.000	-.279	-.127	-.288
		transformative	.047	.001	.138	.009
		fragility	.000	-.199	-.058	-.249
		valuing	.706	-.077	.113	-.104
		inflexibility	.002	.043	.189	.003

Coefficients[a]

Nation	Model		Correlations: Partial	Correlations: Part	Collinearity Statistics: Tolerance	Collinearity Statistics: VIF
Korea	1	(Constant)				
		eudaimonism	.073	.067	.953	1.050
		inclusive	.246	.232	.935	1.069
		externality	-.116	-.107	.682	1.467
		fear	-.085	-.079	.679	1.473
		transformative	.110	.101	.761	1.314
		fragility	-.153	-.142	.864	1.158
		valuing	.059	.054	.862	1.161
		inflexibility	-.018	-.017	.878	1.138
Canada	1	(Constant)				
		eudaimonism	.139	.128	.951	1.051
		inclusive	.162	.149	.908	1.101
		externality	-.065	-.059	.548	1.826
		fear	-.202	-.187	.575	1.738
		transformative	.078	.071	.845	1.183
		fragility	-.139	-.128	.767	1.304
		valuing	.015	.013	.733	1.364
		inflexibility	.121	.111	.764	1.309

a. Dependent Variable: social_well_being

Collinearity Diagnostics[a]

Nation	Model	Dimension	Eigenvalue	Condition Index	Variance Proportions (Constant)	Variance Proportions eudaimonism
Korea	1	1	8.436	1.000	.00	.00
		2	.167	7.107	.00	.12
		3	.106	8.931	.00	.48
		4	.092	9.578	.00	.04
		5	.074	10.661	.00	.25
		6	.053	12.604	.00	.00
		7	.038	14.840	.01	.01
		8	.025	18.508	.04	.02
		9	.009	30.399	.94	.09
Canada	1	1	8.212	1.000	.00	.00
		2	.264	5.575	.00	.06
		3	.145	7.533	.00	.02
		4	.110	8.658	.00	.21
		5	.086	9.784	.00	.18
		6	.074	10.530	.00	.32
		7	.055	12.213	.01	.00
		8	.037	14.804	.01	.01
		9	.017	21.985	.96	.21

Collinearity Diagnostics[a]

Nation	Model	Dimension	Variance Proportions inclusive	externality	fear	transformative	fragility
Korea	1	1	.00	.00	.00	.00	.00
		2	.23	.11	.10	.00	.00
		3	.46	.01	.01	.01	.00
		4	.05	.05	.41	.10	.00
		5	.15	.15	.10	.18	.10
		6	.00	.34	.15	.00	.09
		7	.00	.31	.21	.71	.18
		8	.07	.02	.00	.00	.52
		9	.03	.00	.01	.00	.11
Canada	1	1	.00	.00	.00	.00	.00
		2	.08	.06	.19	.01	.00
		3	.11	.01	.12	.06	.03
		4	.61	.00	.10	.00	.00
		5	.01	.24	.45	.00	.10
		6	.05	.28	.01	.33	.03
		7	.00	.39	.08	.56	.21
		8	.13	.02	.00	.00	.48
		9	.01	.01	.05	.03	.14

Collinearity Diagnostics[a]

Nation	Model	Dimension	Variance Proportions valuing	inflexibility
Korea	1	1	.00	.00
		2	.00	.01
		3	.00	.02
		4	.00	.17
		5	.01	.01
		6	.01	.62
		7	.02	.01
		8	.41	.09
		9	.54	.07
Canada	1	1	.00	.00
		2	.00	.03
		3	.00	.45
		4	.00	.12
		5	.04	.08
		6	.00	.21
		7	.06	.01
		8	.60	.01
		9	.30	.09

a. Dependent Variable: social_well_being

```
 REGRESSION
  /MISSING LISTWISE
  /STATISTICS COEFF OUTS CI(95) R ANOVA COLLIN TOL ZPP
  /CRITERIA=PIN(.05) POUT(.10)
  /NOORIGIN
  /DEPENDENT psychological_well_being
  /METHOD=ENTER eudaimonism inclusive externality fear transformative fragi
lity valuing
    inflexibility.
```

Regression

Notes

Output Created		20-MAY-2019 12:20:08
Comments		
Input	Data	C:\Users\1\Desktop\korea cnada study\merge korea canada\final.sav
	Active Dataset	DataSet1
	Filter	<none>
	Weight	<none>
	Split File	Nation
	N of Rows in Working Data File	1837
Missing Value Handling	Definition of Missing	User-defined missing values are treated as missing.
	Cases Used	Statistics are based on cases with no missing values for any variable used.
Syntax		REGRESSION /MISSING LISTWISE /STATISTICS COEFF OUTS CI(95) R ANOVA COLLIN TOL ZPP /CRITERIA=PIN(.05) POUT(.10) /NOORIGIN /DEPENDENT psychological_well_being /METHOD=ENTER eudaimonism inclusive externality fear transformative fragility valuing inflexibility.
Resources	Processor Time	00:00:00.03
	Elapsed Time	00:00:00.04
	Memory Required	13728 bytes
	Additional Memory Required for Residual Plots	0 bytes

Variables Entered/Removed[a]

Nation	Model	Variables Entered	Variables Removed	Method
Korea	1	inflexibility, transformative, eudaimonism, inclusive, valuing, fragility, externality, fear[b]	.	Enter
Canada	1	inflexibility, transformative, eudaimonism, inclusive, fragility, valuing, fear, externality[b]	.	Enter

a. Dependent Variable: psychological_well_being

b. All requested variables entered.

Model Summary

Nation	Model	R	R Square	Adjusted R Square	Std. Error of the Estimate
Korea	1	.424[a]	.180	.174	.94339
Canada	1	.459[b]	.210	.201	1.13078

a. Predictors: (Constant), inflexibility, transformative, eudaimonism, inclusive, valuing, fragility, externality, fear

b. Predictors: (Constant), inflexibility, transformative, eudaimonism, inclusive, fragility, valuing, fear, externality

ANOVA[a]

Nation	Model		Sum of Squares	df	Mean Square	F	Sig.
Korea	1	Regression	228.060	8	28.507	32.032	.000[b]
		Residual	1039.498	1168	.890		
		Total	1267.558	1176			
Canada	1	Regression	221.756	8	27.720	21.678	.000[c]
		Residual	832.413	651	1.279		
		Total	1054.169	659			

a. Dependent Variable: psychological_well_being

b. Predictors: (Constant), inflexibility, transformative, eudaimonism, inclusive, valuing, fragility, externality, fear

c. Predictors: (Constant), inflexibility, transformative, eudaimonism, inclusive, fragility, valuing, fear, externality

Coefficients[a]

Nation	Model		Unstandardized Coefficients		Standardized Coefficients	t
			B	Std. Error	Beta	
Korea	1	(Constant)	1.947	.249		7.826
		eudaimonism	.015	.005	.077	2.847
		inclusive	.162	.025	.176	6.405
		externality	-.162	.031	-.166	-5.176
		fear	-.175	.031	-.182	-5.649
		transformative	.141	.028	.155	5.105
		fragility	-.152	.028	-.156	-5.464
		valuing	.143	.041	.098	3.441
		inflexibility	-5.964E-5	.027	.000	-.002
Canada	1	(Constant)	2.989	.299		9.984
		eudaimonism	.017	.008	.074	2.072
		inclusive	.172	.035	.180	4.915
		externality	-.126	.045	-.132	-2.800
		fear	-.244	.042	-.268	-5.843
		transformative	.084	.038	.085	2.240
		fragility	-.082	.039	-.084	-2.106
		valuing	-.080	.052	-.062	-1.523
		inflexibility	.062	.040	.062	1.550

Coefficients[a]

Nation	Model		Sig.	95.0% Confidence Interval for B Lower Bound	Upper Bound	Correlations Zero-order
Korea	1	(Constant)	.000	1.459	2.435	
		eudaimonism	.004	.005	.026	.125
		inclusive	.000	.112	.211	.244
		externality	.000	-.223	-.101	-.265
		fear	.000	-.236	-.114	-.211
		transformative	.000	.087	.196	.110
		fragility	.000	-.207	-.098	-.174
		valuing	.001	.061	.224	.009
		inflexibility	.998	-.054	.054	-.088
Canada	1	(Constant)	.000	2.401	3.577	
		eudaimonism	.039	.001	.034	.138
		inclusive	.000	.103	.241	.197
		externality	.005	-.214	-.038	-.311
		fear	.000	-.325	-.162	-.363
		transformative	.025	.010	.158	.007
		fragility	.036	-.158	-.006	-.237
		valuing	.128	-.182	.023	-.192
		inflexibility	.122	-.017	.141	-.096

Coefficients[a]

Nation	Model		Correlations Partial	Correlations Part	Collinearity Statistics Tolerance	Collinearity Statistics VIF
Korea	1	(Constant)				
		eudaimonism	.083	.075	.953	1.050
		inclusive	.184	.170	.935	1.069
		externality	-.150	-.137	.682	1.467
		fear	-.163	-.150	.679	1.473
		transformative	.148	.135	.761	1.314
		fragility	-.158	-.145	.864	1.158
		valuing	.100	.091	.862	1.161
		inflexibility	.000	.000	.878	1.138
Canada	1	(Constant)				
		eudaimonism	.081	.072	.951	1.051
		inclusive	.189	.171	.908	1.101
		externality	-.109	-.098	.548	1.826
		fear	-.223	-.203	.575	1.738
		transformative	.087	.078	.845	1.183
		fragility	-.082	-.073	.767	1.304
		valuing	-.060	-.053	.733	1.364
		inflexibility	.061	.054	.764	1.309

a. Dependent Variable: psychological_well_being

Collinearity Diagnostics[a]

Nation	Model	Dimension	Eigenvalue	Condition Index	Variance Proportions (Constant)	eudaimonism
Korea	1	1	8.436	1.000	.00	.00
		2	.167	7.107	.00	.12
		3	.106	8.931	.00	.48
		4	.092	9.578	.00	.04
		5	.074	10.661	.00	.25
		6	.053	12.604	.00	.00
		7	.038	14.840	.01	.01
		8	.025	18.508	.04	.02
		9	.009	30.399	.94	.09
Canada	1	1	8.212	1.000	.00	.00
		2	.264	5.575	.00	.06
		3	.145	7.533	.00	.02
		4	.110	8.658	.00	.21
		5	.086	9.784	.00	.18
		6	.074	10.530	.00	.32
		7	.055	12.213	.01	.00
		8	.037	14.804	.01	.01
		9	.017	21.985	.96	.21

Collinearity Diagnostics[a]

Nation	Model	Dimension	Variance Proportions inclusive	externality	fear	transformative	fragility
Korea	1	1	.00	.00	.00	.00	.00
		2	.23	.11	.10	.00	.00
		3	.46	.01	.01	.01	.00
		4	.05	.05	.41	.10	.00
		5	.15	.15	.10	.18	.10
		6	.00	.34	.15	.00	.09
		7	.00	.31	.21	.71	.18
		8	.07	.02	.00	.00	.52
		9	.03	.00	.01	.00	.11
Canada	1	1	.00	.00	.00	.00	.00
		2	.08	.06	.19	.01	.00
		3	.11	.01	.12	.06	.03
		4	.61	.00	.10	.00	.00
		5	.01	.24	.45	.00	.10
		6	.05	.28	.01	.33	.03
		7	.00	.39	.08	.56	.21
		8	.13	.02	.00	.00	.48
		9	.01	.01	.05	.03	.14

Collinearity Diagnostics[a]

Nation	Model	Dimension	Variance Proportions valuing	inflexibility
Korea	1	1	.00	.00
		2	.00	.01
		3	.00	.02
		4	.00	.17
		5	.01	.01
		6	.01	.62
		7	.02	.01
		8	.41	.09
		9	.54	.07
Canada	1	1	.00	.00
		2	.00	.03
		3	.00	.45
		4	.00	.12
		5	.04	.08
		6	.00	.21
		7	.06	.01
		8	.60	.01
		9	.30	.09

a. Dependent Variable: psychological_well_being

```
REGRESSION
  /MISSING LISTWISE
  /STATISTICS COEFF OUTS CI(95) R ANOVA COLLIN TOL ZPP
  /CRITERIA=PIN(.05) POUT(.10)
  /NOORIGIN
  /DEPENDENT life_satisfaction
  /METHOD=ENTER eudaimonism inclusive externality fear transformative fragi
lity valuing
    inflexibility.
```

Regression

Notes

Output Created		20-MAY-2019 12:20:15
Comments		
Input	Data	C:\Users\1\Desktop\korea cnada study\merge korea canada\final.sav
	Active Dataset	DataSet1
	Filter	<none>
	Weight	<none>
	Split File	Nation
	N of Rows in Working Data File	1837
Missing Value Handling	Definition of Missing	User-defined missing values are treated as missing.
	Cases Used	Statistics are based on cases with no missing values for any variable used.
Syntax		REGRESSION<br>/MISSING LISTWISE<br>/STATISTICS COEFF OUTS CI(95) R ANOVA COLLIN TOL ZPP<br>/CRITERIA=PIN(.05) POUT(.10)<br>/NOORIGIN<br>/DEPENDENT life_satisfaction<br>/METHOD=ENTER eudaimonism inclusive externality fear transformative fragility valuing<br>inflexibility.
Resources	Processor Time	00:00:00.03
	Elapsed Time	00:00:00.04
	Memory Required	13728 bytes
	Additional Memory Required for Residual Plots	0 bytes

Variables Entered/Removed[a]

Nation	Model	Variables Entered	Variables Removed	Method
Korea	1	inflexibility, transformative , eudaimonism, inclusive, valuing, fragility, externality, fear[b]	.	Enter
Canada	1	inflexibility, transformative , eudaimonism, inclusive, fragility, valuing, fear, externality[b]	.	Enter

a. Dependent Variable: life_satisfaction

b. All requested variables entered.

Model Summary

Nation	Model	R	R Square	Adjusted R Square	Std. Error of the Estimate
Korea	1	.423[a]	.179	.173	1.24190
Canada	1	.491[b]	.241	.231	1.36261

a. Predictors: (Constant), inflexibility, transformative, eudaimonism, inclusive, valuing, fragility, externality, fear

b. Predictors: (Constant), inflexibility, transformative, eudaimonism, inclusive, fragility, valuing, fear, externality

ANOVA[a]

Nation	Model		Sum of Squares	df	Mean Square	F	Sig.
Korea	1	Regression	391.542	8	48.943	31.733	.000[b]
		Residual	1801.418	1168	1.542		
		Total	2192.960	1176			
Canada	1	Regression	383.053	8	47.882	25.789	.000[c]
		Residual	1208.713	651	1.857		
		Total	1591.766	659			

a. Dependent Variable: life_satisfaction

b. Predictors: (Constant), inflexibility, transformative, eudaimonism, inclusive, valuing, fragility, externality, fear

c. Predictors: (Constant), inflexibility, transformative, eudaimonism, inclusive, fragility, valuing, fear, externality

Coefficients[a]

Nation	Model		Unstandardized Coefficients		Standardized Coefficients	t
			B	Std. Error	Beta	
Korea	1	(Constant)	5.046	.327		15.408
		eudaimonism	-.010	.007	-.039	-1.445
		inclusive	.200	.033	.165	6.014
		externality	-.251	.041	-.196	-6.090
		fear	-.167	.041	-.132	-4.087
		transformative	.197	.036	.164	5.400
		fragility	-.239	.037	-.185	-6.501
		valuing	-.095	.055	-.050	-1.739
		inflexibility	.052	.036	.041	1.446
Canada	1	(Constant)	5.027	.361		13.935
		eudaimonism	-.012	.010	-.043	-1.239
		inclusive	.192	.042	.163	4.552
		externality	-.201	.054	-.171	-3.711
		fear	-.321	.050	-.288	-6.394
		transformative	.130	.045	.106	2.853
		fragility	-.136	.047	-.114	-2.920
		valuing	-.143	.063	-.091	-2.274
		inflexibility	.209	.048	.169	4.325

Coefficients[a]

Nation	Model		Sig.	95.0% Confidence Interval for B Lower Bound	95.0% Confidence Interval for B Upper Bound	Correlations Zero-order
Korea	1	(Constant)	.000	4.403	5.688	
		eudaimonism	.149	-.024	.004	.017
		inclusive	.000	.135	.265	.218
		externality	.000	-.332	-.170	-.293
		fear	.000	-.247	-.087	-.214
		transformative	.000	.125	.268	.080
		fragility	.000	-.311	-.167	-.220
		valuing	.082	-.202	.012	-.127
		inflexibility	.148	-.019	.123	-.057
Canada	1	(Constant)	.000	4.319	5.736	
		eudaimonism	.216	-.032	.007	.023
		inclusive	.000	.109	.275	.178
		externality	.000	-.307	-.095	-.322
		fear	.000	-.420	-.223	-.377
		transformative	.004	.040	.219	-.014
		fragility	.004	-.228	-.045	-.278
		valuing	.023	-.267	-.020	-.225
		inflexibility	.000	.114	.304	-.011

Coefficients[a]

Nation	Model		Correlations: Partial	Correlations: Part	Collinearity Statistics: Tolerance	Collinearity Statistics: VIF
Korea	1	(Constant)				
		eudaimonism	-.042	-.038	.953	1.050
		inclusive	.173	.160	.935	1.069
		externality	-.175	-.161	.682	1.467
		fear	-.119	-.108	.679	1.473
		transformative	.156	.143	.761	1.314
		fragility	-.187	-.172	.864	1.158
		valuing	-.051	-.046	.862	1.161
		inflexibility	.042	.038	.878	1.138
Canada	1	(Constant)				
		eudaimonism	-.048	-.042	.951	1.051
		inclusive	.176	.155	.908	1.101
		externality	-.144	-.127	.548	1.826
		fear	-.243	-.218	.575	1.738
		transformative	.111	.097	.845	1.183
		fragility	-.114	-.100	.767	1.304
		valuing	-.089	-.078	.733	1.364
		inflexibility	.167	.148	.764	1.309

a. Dependent Variable: life_satisfaction

Collinearity Diagnostics[a]

					Variance Proportions	
Nation	Model	Dimension	Eigenvalue	Condition Index	(Constant)	eudaimonism
Korea	1	1	8.436	1.000	.00	.00
		2	.167	7.107	.00	.12
		3	.106	8.931	.00	.48
		4	.092	9.578	.00	.04
		5	.074	10.661	.00	.25
		6	.053	12.604	.00	.00
		7	.038	14.840	.01	.01
		8	.025	18.508	.04	.02
		9	.009	30.399	.94	.09
Canada	1	1	8.212	1.000	.00	.00
		2	.264	5.575	.00	.06
		3	.145	7.533	.00	.02
		4	.110	8.658	.00	.21
		5	.086	9.784	.00	.18
		6	.074	10.530	.00	.32
		7	.055	12.213	.01	.00
		8	.037	14.804	.01	.01
		9	.017	21.985	.96	.21

Collinearity Diagnostics[a]

Nation	Model	Dimension	Variance Proportions inclusive	externality	fear	transformative	fragility
Korea	1	1	.00	.00	.00	.00	.00
		2	.23	.11	.10	.00	.00
		3	.46	.01	.01	.01	.00
		4	.05	.05	.41	.10	.00
		5	.15	.15	.10	.18	.10
		6	.00	.34	.15	.00	.09
		7	.00	.31	.21	.71	.18
		8	.07	.02	.00	.00	.52
		9	.03	.00	.01	.00	.11
Canada	1	1	.00	.00	.00	.00	.00
		2	.08	.06	.19	.01	.00
		3	.11	.01	.12	.06	.03
		4	.61	.00	.10	.00	.00
		5	.01	.24	.45	.00	.10
		6	.05	.28	.01	.33	.03
		7	.00	.39	.08	.56	.21
		8	.13	.02	.00	.00	.48
		9	.01	.01	.05	.03	.14

Collinearity Diagnostics[a]

Nation	Model	Dimension	Variance Proportions valuing	inflexibility
Korea	1	1	.00	.00
		2	.00	.01
		3	.00	.02
		4	.00	.17
		5	.01	.01
		6	.01	.62
		7	.02	.01
		8	.41	.09
		9	.54	.07
Canada	1	1	.00	.00
		2	.00	.03
		3	.00	.45
		4	.00	.12
		5	.04	.08
		6	.00	.21
		7	.06	.01
		8	.60	.01
		9	.30	.09

a. Dependent Variable: life_satisfaction

```
REGRESSION
  /MISSING LISTWISE
  /STATISTICS COEFF OUTS CI(95) R ANOVA COLLIN TOL ZPP
  /CRITERIA=PIN(.05) POUT(.10)
  /NOORIGIN
  /DEPENDENT negative_affect
  /METHOD=ENTER eudaimonism inclusive externality fear transformative fragi
lity valuing
    inflexibility.
```

Regression

Notes

Output Created		20-MAY-2019 12:20:20
Comments		
Input	Data	C:\Users\1\Desktop\korea cnada study\merge korea canada\final.sav
	Active Dataset	DataSet1
	Filter	<none>
	Weight	<none>
	Split File	Nation
	N of Rows in Working Data File	1837
Missing Value Handling	Definition of Missing	User-defined missing values are treated as missing.
	Cases Used	Statistics are based on cases with no missing values for any variable used.
Syntax		REGRESSION /MISSING LISTWISE /STATISTICS COEFF OUTS CI(95) R ANOVA COLLIN TOL ZPP /CRITERIA=PIN(.05) POUT(.10) /NOORIGIN /DEPENDENT negative_affect /METHOD=ENTER eudaimonism inclusive externality fear transformative fragility valuing inflexibility.
Resources	Processor Time	00:00:00.03
	Elapsed Time	00:00:00.03
	Memory Required	13728 bytes
	Additional Memory Required for Residual Plots	0 bytes

Variables Entered/Removed[a]

Nation	Model	Variables Entered	Variables Removed	Method
Korea	1	inflexibility, transformative, eudaimonism, inclusive, valuing, fragility, externality, fear[b]	.	Enter
Canada	1	inflexibility, transformative, eudaimonism, inclusive, fragility, valuing, fear, externality[b]	.	Enter

a. Dependent Variable: negative_affect

b. All requested variables entered.

Model Summary

Nation	Model	R	R Square	Adjusted R Square	Std. Error of the Estimate
Korea	1	.473[a]	.224	.219	.68994
Canada	1	.537[b]	.288	.280	.74793

a. Predictors: (Constant), inflexibility, transformative, eudaimonism, inclusive, valuing, fragility, externality, fear

b. Predictors: (Constant), inflexibility, transformative, eudaimonism, inclusive, fragility, valuing, fear, externality

ANOVA[a]

Nation	Model		Sum of Squares	df	Mean Square	F	Sig.
Korea	1	Regression	160.447	8	20.056	42.133	.000[b]
		Residual	555.985	1168	.476		
		Total	716.432	1176			
Canada	1	Regression	147.579	8	18.447	32.977	.000[c]
		Residual	364.171	651	.559		
		Total	511.750	659			

a. Dependent Variable: negative_affect

b. Predictors: (Constant), inflexibility, transformative, eudaimonism, inclusive, valuing, fragility, externality, fear

c. Predictors: (Constant), inflexibility, transformative, eudaimonism, inclusive, fragility, valuing, fear, externality

Coefficients[a]

			Unstandardized Coefficients		Standardized Coefficients	
Nation	Model		B	Std. Error	Beta	t
Korea	1	(Constant)	.157	.182		.864
		eudaimonism	.003	.004	.019	.722
		inclusive	-.033	.018	-.048	-1.806
		externality	.176	.023	.240	7.674
		fear	.119	.023	.164	5.245
		transformative	-.033	.020	-.048	-1.635
		fragility	.092	.020	.126	4.528
		valuing	.212	.030	.194	7.002
		inflexibility	-.048	.020	-.066	-2.399
Canada	1	(Constant)	.500	.198		2.526
		eudaimonism	.002	.005	.012	.367
		inclusive	.003	.023	.005	.141
		externality	.107	.030	.160	3.587
		fear	.157	.028	.248	5.699
		transformative	-.008	.025	-.012	-.332
		fragility	.047	.026	.070	1.846
		valuing	.245	.035	.273	7.079
		inflexibility	-.113	.027	-.162	-4.271

Coefficients[a]

Nation	Model		Sig.	95.0% Confidence Interval for B		Correlations
				Lower Bound	Upper Bound	Zero-order
Korea	1	(Constant)	.388	-.200	.514	
		eudaimonism	.471	-.005	.011	-.014
		inclusive	.071	-.070	.003	-.073
		externality	.000	.131	.221	.366
		fear	.000	.074	.163	.324
		transformative	.102	-.073	.007	.074
		fragility	.000	.052	.132	.225
		valuing	.000	.153	.272	.307
		inflexibility	.017	-.088	-.009	.071
Canada	1	(Constant)	.012	.111	.889	
		eudaimonism	.714	-.009	.013	-.037
		inclusive	.888	-.042	.049	.038
		externality	.000	.048	.165	.365
		fear	.000	.103	.211	.412
		transformative	.740	-.057	.041	.135
		fragility	.065	-.003	.098	.289
		valuing	.000	.177	.313	.426
		inflexibility	.000	-.166	-.061	.044

Coefficients[a]

Nation	Model		Correlations Partial	Correlations Part	Collinearity Statistics Tolerance	Collinearity Statistics VIF
Korea	1	(Constant)				
		eudaimonism	.021	.019	.953	1.050
		inclusive	-.053	-.047	.935	1.069
		externality	.219	.198	.682	1.467
		fear	.152	.135	.679	1.473
		transformative	-.048	-.042	.761	1.314
		fragility	.131	.117	.864	1.158
		valuing	.201	.180	.862	1.161
		inflexibility	-.070	-.062	.878	1.138
Canada	1	(Constant)				
		eudaimonism	.014	.012	.951	1.051
		inclusive	.006	.005	.908	1.101
		externality	.139	.119	.548	1.826
		fear	.218	.188	.575	1.738
		transformative	-.013	-.011	.845	1.183
		fragility	.072	.061	.767	1.304
		valuing	.267	.234	.733	1.364
		inflexibility	-.165	-.141	.764	1.309

a. Dependent Variable: negative_affect

Collinearity Diagnostics[a]

Nation	Model	Dimension	Eigenvalue	Condition Index	Variance Proportions (Constant)	Variance Proportions eudaimonism
Korea	1	1	8.436	1.000	.00	.00
		2	.167	7.107	.00	.12
		3	.106	8.931	.00	.48
		4	.092	9.578	.00	.04
		5	.074	10.661	.00	.25
		6	.053	12.604	.00	.00
		7	.038	14.840	.01	.01
		8	.025	18.508	.04	.02
		9	.009	30.399	.94	.09
Canada	1	1	8.212	1.000	.00	.00
		2	.264	5.575	.00	.06
		3	.145	7.533	.00	.02
		4	.110	8.658	.00	.21
		5	.086	9.784	.00	.18
		6	.074	10.530	.00	.32
		7	.055	12.213	.01	.00
		8	.037	14.804	.01	.01
		9	.017	21.985	.96	.21

Collinearity Diagnostics[a]

Nation	Model	Dimension	Variance Proportions: inclusive	externality	fear	transformative	fragility
Korea	1	1	.00	.00	.00	.00	.00
		2	.23	.11	.10	.00	.00
		3	.46	.01	.01	.01	.00
		4	.05	.05	.41	.10	.00
		5	.15	.15	.10	.18	.10
		6	.00	.34	.15	.00	.09
		7	.00	.31	.21	.71	.18
		8	.07	.02	.00	.00	.52
		9	.03	.00	.01	.00	.11
Canada	1	1	.00	.00	.00	.00	.00
		2	.08	.06	.19	.01	.00
		3	.11	.01	.12	.06	.03
		4	.61	.00	.10	.00	.00
		5	.01	.24	.45	.00	.10
		6	.05	.28	.01	.33	.03
		7	.00	.39	.08	.56	.21
		8	.13	.02	.00	.00	.48
		9	.01	.01	.05	.03	.14

Collinearity Diagnostics[a]

Nation	Model	Dimension	Variance Proportions valuing	inflexibility
Korea	1	1	.00	.00
		2	.00	.01
		3	.00	.02
		4	.00	.17
		5	.01	.01
		6	.01	.62
		7	.02	.01
		8	.41	.09
		9	.54	.07
Canada	1	1	.00	.00
		2	.00	.03
		3	.00	.45
		4	.00	.12
		5	.04	.08
		6	.00	.21
		7	.06	.01
		8	.60	.01
		9	.30	.09

a. Dependent Variable: negative_affect

```
REGRESSION
  /MISSING LISTWISE
  /STATISTICS COEFF OUTS CI(95) R ANOVA COLLIN TOL ZPP
  /CRITERIA=PIN(.05) POUT(.10)
  /NOORIGIN
  /DEPENDENT positive_affect
  /METHOD=ENTER eudaimonism inclusive externality fear transformative fragi
lity valuing
    inflexibility.
```

Regression

Notes

Output Created		20-MAY-2019 12:20:28
Comments		
Input	Data	C:\Users\1\Desktop\korea cnada study\merge korea canada\final.sav
	Active Dataset	DataSet1
	Filter	<none>
	Weight	<none>
	Split File	Nation
	N of Rows in Working Data File	1837
Missing Value Handling	Definition of Missing	User-defined missing values are treated as missing.
	Cases Used	Statistics are based on cases with no missing values for any variable used.
Syntax		REGRESSION /MISSING LISTWISE /STATISTICS COEFF OUTS CI(95) R ANOVA COLLIN TOL ZPP /CRITERIA=PIN(.05) POUT(.10) /NOORIGIN /DEPENDENT positive_affect /METHOD=ENTER eudaimonism inclusive externality fear transformative fragility valuing inflexibility.
Resources	Processor Time	00:00:00.02
	Elapsed Time	00:00:00.03
	Memory Required	13728 bytes
	Additional Memory Required for Residual Plots	0 bytes

Variables Entered/Removed[a]

Nation	Model	Variables Entered	Variables Removed	Method
Korea	1	inflexibility, transformative, eudaimonism, inclusive, valuing, fragility, externality, fear[b]	.	Enter
Canada	1	inflexibility, transformative, eudaimonism, inclusive, fragility, valuing, fear, externality[b]	.	Enter

a. Dependent Variable: positive_affect

b. All requested variables entered.

Model Summary

Nation	Model	R	R Square	Adjusted R Square	Std. Error of the Estimate
Korea	1	.432[a]	.186	.181	.67877
Canada	1	.551[b]	.304	.295	.72388

a. Predictors: (Constant), inflexibility, transformative, eudaimonism, inclusive, valuing, fragility, externality, fear

b. Predictors: (Constant), inflexibility, transformative, eudaimonism, inclusive, fragility, valuing, fear, externality

ANOVA[a]

Nation	Model		Sum of Squares	df	Mean Square	F	Sig.
Korea	1	Regression	123.311	8	15.414	33.456	.000[b]
		Residual	538.127	1168	.461		
		Total	661.438	1176			
Canada	1	Regression	148.972	8	18.621	35.537	.000[c]
		Residual	341.122	651	.524		
		Total	490.093	659			

a. Dependent Variable: positive_affect

b. Predictors: (Constant), inflexibility, transformative, eudaimonism, inclusive, valuing, fragility, externality, fear

c. Predictors: (Constant), inflexibility, transformative, eudaimonism, inclusive, fragility, valuing, fear, externality

Coefficients[a]

Nation	Model		Unstandardized Coefficients		Standardized Coefficients	t
			B	Std. Error	Beta	
Korea	1	(Constant)	3.569	.179		19.942
		eudaimonism	-.009	.004	-.061	-2.258
		inclusive	.117	.018	.176	6.447
		externality	-.132	.023	-.187	-5.853
		fear	-.126	.022	-.181	-5.636
		transformative	.114	.020	.174	5.736
		fragility	-.110	.020	-.156	-5.479
		valuing	-.024	.030	-.023	-.820
		inflexibility	.015	.020	.021	.739
Canada	1	(Constant)	3.522	.192		18.377
		eudaimonism	.000	.005	.003	.083
		inclusive	.131	.022	.200	5.820
		externality	-.138	.029	-.212	-4.797
		fear	-.201	.027	-.324	-7.516
		transformative	.066	.024	.097	2.718
		fragility	-.058	.025	-.088	-2.361
		valuing	-.082	.033	-.094	-2.461
		inflexibility	.125	.026	.182	4.865

Coefficients[a]

Nation	Model		Sig.	95.0% Confidence Interval for B Lower Bound	Upper Bound	Correlations Zero-order
Korea	1	(Constant)	.000	3.218	3.920	
		eudaimonism	.024	-.016	-.001	-.004
		inclusive	.000	.082	.153	.227
		externality	.000	-.176	-.088	-.300
		fear	.000	-.170	-.082	-.246
		transformative	.000	.075	.153	.085
		fragility	.000	-.149	-.071	-.193
		valuing	.413	-.083	.034	-.105
		inflexibility	.460	-.024	.053	-.078
Canada	1	(Constant)	.000	3.146	3.898	
		eudaimonism	.934	-.010	.011	.076
		inclusive	.000	.087	.175	.217
		externality	.000	-.194	-.081	-.376
		fear	.000	-.253	-.148	-.430
		transformative	.007	.018	.113	-.019
		fragility	.019	-.107	-.010	-.287
		valuing	.014	-.148	-.017	-.247
		inflexibility	.000	.075	.175	-.028

Coefficients[a]

Nation	Model		Correlations Partial	Correlations Part	Collinearity Statistics Tolerance	Collinearity Statistics VIF
Korea	1	(Constant)				
		eudaimonism	-.066	-.060	.953	1.050
		inclusive	.185	.170	.935	1.069
		externality	-.169	-.154	.682	1.467
		fear	-.163	-.149	.679	1.473
		transformative	.166	.151	.761	1.314
		fragility	-.158	-.145	.864	1.158
		valuing	-.024	-.022	.862	1.161
		inflexibility	.022	.019	.878	1.138
Canada	1	(Constant)				
		eudaimonism	.003	.003	.951	1.051
		inclusive	.222	.190	.908	1.101
		externality	-.185	-.157	.548	1.826
		fear	-.283	-.246	.575	1.738
		transformative	.106	.089	.845	1.183
		fragility	-.092	-.077	.767	1.304
		valuing	-.096	-.080	.733	1.364
		inflexibility	.187	.159	.764	1.309

a. Dependent Variable: positive_affect

Collinearity Diagnostics[a]

Nation	Model	Dimension	Eigenvalue	Condition Index	Variance Proportions (Constant)	eudaimonism
Korea	1	1	8.436	1.000	.00	.00
		2	.167	7.107	.00	.12
		3	.106	8.931	.00	.48
		4	.092	9.578	.00	.04
		5	.074	10.661	.00	.25
		6	.053	12.604	.00	.00
		7	.038	14.840	.01	.01
		8	.025	18.508	.04	.02
		9	.009	30.399	.94	.09
Canada	1	1	8.212	1.000	.00	.00
		2	.264	5.575	.00	.06
		3	.145	7.533	.00	.02
		4	.110	8.658	.00	.21
		5	.086	9.784	.00	.18
		6	.074	10.530	.00	.32
		7	.055	12.213	.01	.00
		8	.037	14.804	.01	.01
		9	.017	21.985	.96	.21

Collinearity Diagnostics[a]

Nation	Model	Dimension	Variance Proportions inclusive	externality	fear	transformative	fragility
Korea	1	1	.00	.00	.00	.00	.00
		2	.23	.11	.10	.00	.00
		3	.46	.01	.01	.01	.00
		4	.05	.05	.41	.10	.00
		5	.15	.15	.10	.18	.10
		6	.00	.34	.15	.00	.09
		7	.00	.31	.21	.71	.18
		8	.07	.02	.00	.00	.52
		9	.03	.00	.01	.00	.11
Canada	1	1	.00	.00	.00	.00	.00
		2	.08	.06	.19	.01	.00
		3	.11	.01	.12	.06	.03
		4	.61	.00	.10	.00	.00
		5	.01	.24	.45	.00	.10
		6	.05	.28	.01	.33	.03
		7	.00	.39	.08	.56	.21
		8	.13	.02	.00	.00	.48
		9	.01	.01	.05	.03	.14

Collinearity Diagnostics[a]

Nation	Model	Dimension	Variance Proportions valuing	Variance Proportions inflexibility
Korea	1	1	.00	.00
		2	.00	.01
		3	.00	.02
		4	.00	.17
		5	.01	.01
		6	.01	.62
		7	.02	.01
		8	.41	.09
		9	.54	.07
Canada	1	1	.00	.00
		2	.00	.03
		3	.00	.45
		4	.00	.12
		5	.04	.08
		6	.00	.21
		7	.06	.01
		8	.60	.01
		9	.30	.09

a. Dependent Variable: positive_affect

```
REGRESSION
  /MISSING LISTWISE
  /STATISTICS COEFF OUTS CI(95) R ANOVA COLLIN TOL ZPP
  /CRITERIA=PIN(.05) POUT(.10)
  /NOORIGIN
  /DEPENDENT eudaimonism
  /METHOD=ENTER extraversion agreeable conscientiousness neuroticism openne
ss.
```

Regression

Notes

Output Created		20-MAY-2019 12:20:59
Comments		
Input	Data	C:\Users\1\Desktop\korea cnada study\merge korea canada\final.sav
	Active Dataset	DataSet1
	Filter	<none>
	Weight	<none>
	Split File	Nation
	N of Rows in Working Data File	1837
Missing Value Handling	Definition of Missing	User-defined missing values are treated as missing.
	Cases Used	Statistics are based on cases with no missing values for any variable used.
Syntax		REGRESSION /MISSING LISTWISE /STATISTICS COEFF OUTS CI(95) R ANOVA COLLIN TOL ZPP /CRITERIA=PIN(.05) POUT(.10) /NOORIGIN /DEPENDENT eudaimonism /METHOD=ENTER extraversion agreeable conscientiousness neuroticism openness.
Resources	Processor Time	00:00:00.02
	Elapsed Time	00:00:00.04
	Memory Required	11408 bytes
	Additional Memory Required for Residual Plots	0 bytes

Variables Entered/Removed[a]

Nation	Model	Variables Entered	Variables Removed	Method
Korea	1	openness, neuroticism, agreeable, conscientiousness, extraversion[b]	.	Enter
Canada	1	openness, conscientiousness, extraversion, agreeable, neuroticism[b]	.	Enter

a. Dependent Variable: eudaimonism

b. All requested variables entered.

Model Summary

Nation	Model	R	R Square	Adjusted R Square	Std. Error of the Estimate
Korea	1	.157[a]	.025	.020	5.20151
Canada	1	.268[b]	.072	.065	5.25456

a. Predictors: (Constant), openness, neuroticism, agreeable, conscientiousness, extraversion

b. Predictors: (Constant), openness, conscientiousness, extraversion, agreeable, neuroticism

ANOVA[a]

Nation	Model		Sum of Squares	df	Mean Square	F	Sig.
Korea	1	Regression	796.497	5	159.299	5.888	.000[b]
		Residual	31682.273	1171	27.056		
		Total	32478.769	1176			
Canada	1	Regression	1401.843	5	280.369	10.154	.000[c]
		Residual	18057.194	654	27.610		
		Total	19459.037	659			

a. Dependent Variable: eudaimonism

b. Predictors: (Constant), openness, neuroticism, agreeable, conscientiousness, extraversion

c. Predictors: (Constant), openness, conscientiousness, extraversion, agreeable, neuroticism

Coefficients[a]

Nation	Model		Unstandardized Coefficients B	Unstandardized Coefficients Std. Error	Standardized Coefficients Beta	t
Korea	1	(Constant)	13.833	1.460		9.475
		extraversion	.346	.247	.047	1.404
		agreeable	-.428	.277	-.052	-1.544
		conscientiousness	.603	.228	.080	2.646
		neuroticism	-.706	.228	-.092	-3.097
		openness	.395	.240	.050	1.646
Canada	1	(Constant)	9.686	1.834		5.280
		extraversion	-.265	.225	-.048	-1.179
		agreeable	1.070	.273	.160	3.915
		conscientiousness	-.119	.261	-.019	-.455
		neuroticism	-.288	.244	-.049	-1.183
		openness	1.289	.273	.184	4.721

Coefficients[a]

Nation	Model		Sig.	95.0% Confidence Interval for B Lower Bound	95.0% Confidence Interval for B Upper Bound	Correlations Zero-order
Korea	1	(Constant)	.000	10.969	16.698	
		extraversion	.160	-.137	.830	.044
		agreeable	.123	-.971	.116	-.005
		conscientiousness	.008	.156	1.050	.109
		neuroticism	.002	-1.153	-.259	-.110
		openness	.100	-.076	.865	.069
Canada	1	(Constant)	.000	6.084	13.288	
		extraversion	.239	-.706	.176	.037
		agreeable	.000	.533	1.607	.194
		conscientiousness	.649	-.632	.394	.043
		neuroticism	.237	-.766	.190	-.074
		openness	.000	.753	1.825	.217

Coefficients[a]

Nation	Model		Correlations: Partial	Correlations: Part	Collinearity Statistics: Tolerance	Collinearity Statistics: VIF
Korea	1	(Constant)				
		extraversion	.041	.041	.739	1.353
		agreeable	-.045	-.045	.744	1.344
		conscientiousness	.077	.076	.914	1.094
		neuroticism	-.090	-.089	.953	1.049
		openness	.048	.048	.918	1.089
Canada	1	(Constant)				
		extraversion	-.046	-.044	.865	1.156
		agreeable	.151	.147	.847	1.180
		conscientiousness	-.018	-.017	.851	1.175
		neuroticism	-.046	-.045	.834	1.198
		openness	.182	.178	.934	1.070

a. Dependent Variable: eudaimonism

Collinearity Diagnostics[a]

Nation	Model	Dimension	Eigenvalue	Condition Index	Variance Proportions: (Constant)	Variance Proportions: extraversion
Korea	1	1	5.830	1.000	.00	.00
		2	.064	9.551	.00	.12
		3	.047	11.186	.01	.38
		4	.028	14.317	.00	.01
		5	.023	16.050	.00	.48
		6	.009	26.051	.99	.00
Canada	1	1	5.709	1.000	.00	.00
		2	.137	6.465	.00	.27
		3	.072	8.933	.00	.64
		4	.041	11.775	.00	.03
		5	.031	13.511	.00	.03
		6	.010	23.848	1.00	.03

Collinearity Diagnostics[a]

Nation	Model	Dimension	Variance Proportions agreeable	conscientiousness	neuroticism	openness
Korea	1	1	.00	.00	.00	.00
		2	.02	.02	.57	.00
		3	.03	.26	.05	.08
		4	.00	.36	.01	.77
		5	.82	.05	.02	.03
		6	.13	.31	.35	.12
Canada	1	1	.00	.00	.00	.00
		2	.00	.01	.33	.00
		3	.02	.18	.17	.02
		4	.02	.37	.06	.59
		5	.86	.12	.00	.23
		6	.09	.32	.43	.15

a. Dependent Variable: eudaimonism

```
REGRESSION
  /MISSING LISTWISE
  /STATISTICS COEFF OUTS CI(95) R ANOVA COLLIN TOL ZPP
  /CRITERIA=PIN(.05) POUT(.10)
  /NOORIGIN
  /DEPENDENT inclusive
  /METHOD=ENTER extraversion agreeable conscientiousness neuroticism openne
ss.
```

Regression

Notes

Output Created		20-MAY-2019 12:21:07
Comments		
Input	Data	C:\Users\1\Desktop\korea cnada study\merge korea canada\final.sav
	Active Dataset	DataSet1
	Filter	<none>
	Weight	<none>
	Split File	Nation
	N of Rows in Working Data File	1837
Missing Value Handling	Definition of Missing	User-defined missing values are treated as missing.
	Cases Used	Statistics are based on cases with no missing values for any variable used.
Syntax		REGRESSION /MISSING LISTWISE /STATISTICS COEFF OUTS CI(95) R ANOVA COLLIN TOL ZPP /CRITERIA=PIN(.05) POUT(.10) /NOORIGIN /DEPENDENT inclusive /METHOD=ENTER extraversion agreeable conscientiousness neuroticism openness.
Resources	Processor Time	00:00:00.02
	Elapsed Time	00:00:00.02
	Memory Required	11408 bytes
	Additional Memory Required for Residual Plots	0 bytes

Variables Entered/Removed[a]

Nation	Model	Variables Entered	Variables Removed	Method
Korea	1	openness, neuroticism, agreeable, conscientiousness, extraversion[b]	.	Enter
Canada	1	openness, conscientiousness, extraversion, agreeable, neuroticism[b]	.	Enter

a. Dependent Variable: inclusive

b. All requested variables entered.

Model Summary

Nation	Model	R	R Square	Adjusted R Square	Std. Error of the Estimate
Korea	1	.149[a]	.022	.018	1.11637
Canada	1	.240[b]	.058	.050	1.28504

a. Predictors: (Constant), openness, neuroticism, agreeable, conscientiousness, extraversion

b. Predictors: (Constant), openness, conscientiousness, extraversion, agreeable, neuroticism

ANOVA[a]

Nation	Model		Sum of Squares	df	Mean Square	F	Sig.
Korea	1	Regression	33.001	5	6.600	5.296	.000[b]
		Residual	1459.408	1171	1.246		
		Total	1492.410	1176			
Canada	1	Regression	65.955	5	13.191	7.988	.000[c]
		Residual	1079.967	654	1.651		
		Total	1145.922	659			

a. Dependent Variable: inclusive

b. Predictors: (Constant), openness, neuroticism, agreeable, conscientiousness, extraversion

c. Predictors: (Constant), openness, conscientiousness, extraversion, agreeable, neuroticism

Coefficients[a]

Nation	Model		Unstandardized Coefficients B	Unstandardized Coefficients Std. Error	Standardized Coefficients Beta	t
Korea	1	(Constant)	2.543	.313		8.116
		extraversion	.031	.053	.020	.589
		agreeable	.165	.059	.093	2.776
		conscientiousness	.024	.049	.015	.493
		neuroticism	-.144	.049	-.087	-2.943
		openness	.037	.051	.022	.726
Canada	1	(Constant)	1.754	.449		3.909
		extraversion	.200	.055	.149	3.645
		agreeable	.166	.067	.102	2.482
		conscientiousness	-.100	.064	-.064	-1.564
		neuroticism	.030	.060	.021	.496
		openness	.175	.067	.103	2.619

Coefficients[a]

Nation	Model		Sig.	95.0% Confidence Interval for B Lower Bound	95.0% Confidence Interval for B Upper Bound	Correlations Zero-order
Korea	1	(Constant)	.000	1.928	3.158	
		extraversion	.556	-.073	.135	.074
		agreeable	.006	.048	.282	.112
		conscientiousness	.622	-.072	.120	.051
		neuroticism	.003	-.240	-.048	-.095
		openness	.468	-.064	.138	.052
Canada	1	(Constant)	.000	.873	2.635	
		extraversion	.000	.092	.308	.179
		agreeable	.013	.035	.297	.151
		conscientiousness	.118	-.225	.025	-.017
		neuroticism	.620	-.087	.146	-.023
		openness	.009	.044	.306	.144

Coefficients[a]

Nation	Model		Correlations Partial	Correlations Part	Collinearity Statistics Tolerance	Collinearity Statistics VIF
Korea	1	(Constant)				
		extraversion	.017	.017	.739	1.353
		agreeable	.081	.080	.744	1.344
		conscientiousness	.014	.014	.914	1.094
		neuroticism	-.086	-.085	.953	1.049
		openness	.021	.021	.918	1.089
Canada	1	(Constant)				
		extraversion	.141	.138	.865	1.156
		agreeable	.097	.094	.847	1.180
		conscientiousness	-.061	-.059	.851	1.175
		neuroticism	.019	.019	.834	1.198
		openness	.102	.099	.934	1.070

a. Dependent Variable: inclusive

Collinearity Diagnostics[a]

Nation	Model	Dimension	Eigenvalue	Condition Index	Variance Proportions (Constant)	Variance Proportions extraversion
Korea	1	1	5.830	1.000	.00	.00
		2	.064	9.551	.00	.12
		3	.047	11.186	.01	.38
		4	.028	14.317	.00	.01
		5	.023	16.050	.00	.48
		6	.009	26.051	.99	.00
Canada	1	1	5.709	1.000	.00	.00
		2	.137	6.465	.00	.27
		3	.072	8.933	.00	.64
		4	.041	11.775	.00	.03
		5	.031	13.511	.00	.03
		6	.010	23.848	1.00	.03

Collinearity Diagnostics[a]

Nation	Model	Dimension	Variance Proportions agreeable	conscientiousness	neuroticism	openness
Korea	1	1	.00	.00	.00	.00
		2	.02	.02	.57	.00
		3	.03	.26	.05	.08
		4	.00	.36	.01	.77
		5	.82	.05	.02	.03
		6	.13	.31	.35	.12
Canada	1	1	.00	.00	.00	.00
		2	.00	.01	.33	.00
		3	.02	.18	.17	.02
		4	.02	.37	.06	.59
		5	.86	.12	.00	.23
		6	.09	.32	.43	.15

a. Dependent Variable: inclusive

```
REGRESSION
  /MISSING LISTWISE
  /STATISTICS COEFF OUTS CI(95) R ANOVA COLLIN TOL ZPP
  /CRITERIA=PIN(.05) POUT(.10)
  /NOORIGIN
  /DEPENDENT externality
  /METHOD=ENTER extraversion agreeable conscientiousness neuroticism openne
ss.
```

Regression

Notes

Output Created		20-MAY-2019 12:21:13
Comments		
Input	Data	C:\Users\1\Desktop\korea cnada study\merge korea canada\final.sav
	Active Dataset	DataSet1
	Filter	<none>
	Weight	<none>
	Split File	Nation
	N of Rows in Working Data File	1837
Missing Value Handling	Definition of Missing	User-defined missing values are treated as missing.
	Cases Used	Statistics are based on cases with no missing values for any variable used.
Syntax		REGRESSION<br>/MISSING LISTWISE<br>/STATISTICS COEFF OUTS CI(95) R ANOVA COLLIN TOL ZPP<br>/CRITERIA=PIN(.05) POUT(.10)<br>/NOORIGIN<br>/DEPENDENT externality<br>/METHOD=ENTER extraversion agreeable conscientiousness neuroticism openness.
Resources	Processor Time	00:00:00.02
	Elapsed Time	00:00:00.07
	Memory Required	11408 bytes
	Additional Memory Required for Residual Plots	0 bytes

Variables Entered/Removed[a]

Nation	Model	Variables Entered	Variables Removed	Method
Korea	1	openness, neuroticism, agreeable, conscientiousness, extraversion[b]	.	Enter
Canada	1	openness, conscientiousness, extraversion, agreeable, neuroticism[b]	.	Enter

a. Dependent Variable: externality

b. All requested variables entered.

Model Summary

Nation	Model	R	R Square	Adjusted R Square	Std. Error of the Estimate
Korea	1	.288[a]	.083	.079	1.02161
Canada	1	.392[b]	.154	.147	1.22382

a. Predictors: (Constant), openness, neuroticism, agreeable, conscientiousness, extraversion

b. Predictors: (Constant), openness, conscientiousness, extraversion, agreeable, neuroticism

ANOVA[a]

Nation	Model		Sum of Squares	df	Mean Square	F	Sig.
Korea	1	Regression	110.330	5	22.066	21.142	.000[b]
		Residual	1222.161	1171	1.044		
		Total	1332.491	1176			
Canada	1	Regression	177.622	5	35.524	23.719	.000[c]
		Residual	979.518	654	1.498		
		Total	1157.141	659			

a. Dependent Variable: externality

b. Predictors: (Constant), openness, neuroticism, agreeable, conscientiousness, extraversion

c. Predictors: (Constant), openness, conscientiousness, extraversion, agreeable, neuroticism

Coefficients[a]

Nation	Model		Unstandardized Coefficients B	Unstandardized Coefficients Std. Error	Standardized Coefficients Beta	t
Korea	1	(Constant)	3.197	.287		11.150
		extraversion	-.078	.048	-.053	-1.616
		agreeable	-.129	.054	-.077	-2.376
		conscientiousness	-.047	.045	-.031	-1.054
		neuroticism	.358	.045	.230	8.006
		openness	-.110	.047	-.068	-2.326
Canada	1	(Constant)	4.632	.427		10.841
		extraversion	-.228	.052	-.169	-4.361
		agreeable	-.213	.064	-.131	-3.339
		conscientiousness	-.140	.061	-.090	-2.297
		neuroticism	.244	.057	.170	4.310
		openness	-.115	.064	-.067	-1.809

Coefficients[a]

Nation	Model		Sig.	95.0% Confidence Interval for B Lower Bound	95.0% Confidence Interval for B Upper Bound	Correlations Zero-order
Korea	1	(Constant)	.000	2.634	3.760	
		extraversion	.106	-.173	.017	-.113
		agreeable	.018	-.236	-.023	-.129
		conscientiousness	.292	-.135	.041	-.108
		neuroticism	.000	.271	.446	.244
		openness	.020	-.202	-.017	-.113
Canada	1	(Constant)	.000	3.793	5.471	
		extraversion	.000	-.331	-.125	-.275
		agreeable	.001	-.338	-.088	-.246
		conscientiousness	.022	-.259	-.020	-.210
		neuroticism	.000	.133	.356	.273
		openness	.071	-.240	.010	-.147

Coefficients[a]

Nation	Model		Correlations Partial	Correlations Part	Collinearity Statistics Tolerance	Collinearity Statistics VIF
Korea	1	(Constant)				
		extraversion	-.047	-.045	.739	1.353
		agreeable	-.069	-.066	.744	1.344
		conscientiousness	-.031	-.030	.914	1.094
		neuroticism	.228	.224	.953	1.049
		openness	-.068	-.065	.918	1.089
Canada	1	(Constant)				
		extraversion	-.168	-.157	.865	1.156
		agreeable	-.129	-.120	.847	1.180
		conscientiousness	-.089	-.083	.851	1.175
		neuroticism	.166	.155	.834	1.198
		openness	-.071	-.065	.934	1.070

a. Dependent Variable: externality

Collinearity Diagnostics[a]

Nation	Model	Dimension	Eigenvalue	Condition Index	Variance Proportions (Constant)	Variance Proportions extraversion
Korea	1	1	5.830	1.000	.00	.00
		2	.064	9.551	.00	.12
		3	.047	11.186	.01	.38
		4	.028	14.317	.00	.01
		5	.023	16.050	.00	.48
		6	.009	26.051	.99	.00
Canada	1	1	5.709	1.000	.00	.00
		2	.137	6.465	.00	.27
		3	.072	8.933	.00	.64
		4	.041	11.775	.00	.03
		5	.031	13.511	.00	.03
		6	.010	23.848	1.00	.03

Collinearity Diagnostics[a]

Nation	Model	Dimension	Variance Proportions agreeable	conscientiousness	neuroticism	openness
Korea	1	1	.00	.00	.00	.00
		2	.02	.02	.57	.00
		3	.03	.26	.05	.08
		4	.00	.36	.01	.77
		5	.82	.05	.02	.03
		6	.13	.31	.35	.12
Canada	1	1	.00	.00	.00	.00
		2	.00	.01	.33	.00
		3	.02	.18	.17	.02
		4	.02	.37	.06	.59
		5	.86	.12	.00	.23
		6	.09	.32	.43	.15

a. Dependent Variable: externality

```
REGRESSION
  /MISSING LISTWISE
  /STATISTICS COEFF OUTS CI(95) R ANOVA COLLIN TOL ZPP
  /CRITERIA=PIN(.05) POUT(.10)
  /NOORIGIN
  /DEPENDENT fear
  /METHOD=ENTER extraversion agreeable conscientiousness neuroticism openne
ss.
```

Regression

Notes

Output Created		20-MAY-2019 12:21:20
Comments		
Input	Data	C:\Users\1\Desktop\korea cnada study\merge korea canada\final.sav
	Active Dataset	DataSet1
	Filter	<none>
	Weight	<none>
	Split File	Nation
	N of Rows in Working Data File	1837
Missing Value Handling	Definition of Missing	User-defined missing values are treated as missing.
	Cases Used	Statistics are based on cases with no missing values for any variable used.
Syntax		REGRESSION /MISSING LISTWISE /STATISTICS COEFF OUTS CI(95) R ANOVA COLLIN TOL ZPP /CRITERIA=PIN(.05) POUT(.10) /NOORIGIN /DEPENDENT fear /METHOD=ENTER extraversion agreeable conscientiousness neuroticism openness.
Resources	Processor Time	00:00:00.02
	Elapsed Time	00:00:00.03
	Memory Required	11408 bytes
	Additional Memory Required for Residual Plots	0 bytes

Variables Entered/Removed[a]

Nation	Model	Variables Entered	Variables Removed	Method
Korea	1	openness, neuroticism, agreeable, conscientiousness, extraversion[b]	.	Enter
Canada	1	openness, conscientiousness, extraversion, agreeable, neuroticism[b]	.	Enter

a. Dependent Variable: fear

b. All requested variables entered.

Model Summary

Nation	Model	R	R Square	Adjusted R Square	Std. Error of the Estimate
Korea	1	.227[a]	.052	.047	1.05078
Canada	1	.446[b]	.199	.193	1.25186

a. Predictors: (Constant), openness, neuroticism, agreeable, conscientiousness, extraversion

b. Predictors: (Constant), openness, conscientiousness, extraversion, agreeable, neuroticism

ANOVA[a]

Nation	Model		Sum of Squares	df	Mean Square	F	Sig.
Korea	1	Regression	70.208	5	14.042	12.717	.000[b]
		Residual	1292.937	1171	1.104		
		Total	1363.146	1176			
Canada	1	Regression	254.135	5	50.827	32.433	.000[c]
		Residual	1024.921	654	1.567		
		Total	1279.056	659			

a. Dependent Variable: fear

b. Predictors: (Constant), openness, neuroticism, agreeable, conscientiousness, extraversion

c. Predictors: (Constant), openness, conscientiousness, extraversion, agreeable, neuroticism

Coefficients[a]

Nation	Model		Unstandardized Coefficients B	Unstandardized Coefficients Std. Error	Standardized Coefficients Beta	t
Korea	1	(Constant)	3.419	.295		11.591
		extraversion	-.048	.050	-.032	-.969
		agreeable	-.179	.056	-.106	-3.201
		conscientiousness	-.085	.046	-.055	-1.843
		neuroticism	.223	.046	.141	4.840
		openness	-.078	.048	-.048	-1.603
Canada	1	(Constant)	4.005	.437		9.163
		extraversion	-.179	.054	-.126	-3.338
		agreeable	-.326	.065	-.190	-5.003
		conscientiousness	-.181	.062	-.111	-2.915
		neuroticism	.351	.058	.232	6.058
		openness	-.038	.065	-.021	-.579

Coefficients[a]

Nation	Model		Sig.	95.0% Confidence Interval for B Lower Bound	95.0% Confidence Interval for B Upper Bound	Correlations Zero-order
Korea	1	(Constant)	.000	2.840	3.997	
		extraversion	.333	-.146	.049	-.104
		agreeable	.001	-.289	-.069	-.143
		conscientiousness	.066	-.175	.005	-.111
		neuroticism	.000	.132	.313	.160
		openness	.109	-.173	.017	-.094
Canada	1	(Constant)	.000	3.147	4.863	
		extraversion	.001	-.284	-.074	-.261
		agreeable	.000	-.454	-.198	-.298
		conscientiousness	.004	-.303	-.059	-.255
		neuroticism	.000	.238	.465	.339
		openness	.563	-.165	.090	-.114

Coefficients[a]

Nation	Model		Correlations Partial	Correlations Part	Collinearity Statistics Tolerance	Collinearity Statistics VIF
Korea	1	(Constant)				
		extraversion	-.028	-.028	.739	1.353
		agreeable	-.093	-.091	.744	1.344
		conscientiousness	-.054	-.052	.914	1.094
		neuroticism	.140	.138	.953	1.049
		openness	-.047	-.046	.918	1.089
Canada	1	(Constant)				
		extraversion	-.129	-.117	.865	1.156
		agreeable	-.192	-.175	.847	1.180
		conscientiousness	-.113	-.102	.851	1.175
		neuroticism	.231	.212	.834	1.198
		openness	-.023	-.020	.934	1.070

a. Dependent Variable: fear

Collinearity Diagnostics[a]

Nation	Model	Dimension	Eigenvalue	Condition Index	Variance Proportions (Constant)	Variance Proportions extraversion
Korea	1	1	5.830	1.000	.00	.00
		2	.064	9.551	.00	.12
		3	.047	11.186	.01	.38
		4	.028	14.317	.00	.01
		5	.023	16.050	.00	.48
		6	.009	26.051	.99	.00
Canada	1	1	5.709	1.000	.00	.00
		2	.137	6.465	.00	.27
		3	.072	8.933	.00	.64
		4	.041	11.775	.00	.03
		5	.031	13.511	.00	.03
		6	.010	23.848	1.00	.03

Collinearity Diagnostics[a]

Nation	Model	Dimension	Variance Proportions agreeable	conscientiousness	neuroticism	openness
Korea	1	1	.00	.00	.00	.00
		2	.02	.02	.57	.00
		3	.03	.26	.05	.08
		4	.00	.36	.01	.77
		5	.82	.05	.02	.03
		6	.13	.31	.35	.12
Canada	1	1	.00	.00	.00	.00
		2	.00	.01	.33	.00
		3	.02	.18	.17	.02
		4	.02	.37	.06	.59
		5	.86	.12	.00	.23
		6	.09	.32	.43	.15

a. Dependent Variable: fear

```
REGRESSION
  /MISSING LISTWISE
  /STATISTICS COEFF OUTS CI(95) R ANOVA COLLIN TOL ZPP
  /CRITERIA=PIN(.05) POUT(.10)
  /NOORIGIN
  /DEPENDENT transformative
  /METHOD=ENTER extraversion agreeable conscientiousness neuroticism openne
ss.
```

Regression

Notes

Output Created		20-MAY-2019 12:21:27
Comments		
Input	Data	C:\Users\1\Desktop\korea cnada study\merge korea canada\final.sav
	Active Dataset	DataSet1
	Filter	<none>
	Weight	<none>
	Split File	Nation
	N of Rows in Working Data File	1837
Missing Value Handling	Definition of Missing	User-defined missing values are treated as missing.
	Cases Used	Statistics are based on cases with no missing values for any variable used.
Syntax		REGRESSION<br>/MISSING LISTWISE<br>/STATISTICS COEFF OUTS CI(95) R ANOVA COLLIN TOL ZPP<br>/CRITERIA=PIN(.05) POUT(.10)<br>/NOORIGIN<br>/DEPENDENT transformative<br>/METHOD=ENTER extraversion agreeable conscientiousness neuroticism openness.
Resources	Processor Time	00:00:00.03
	Elapsed Time	00:00:00.05
	Memory Required	11408 bytes
	Additional Memory Required for Residual Plots	0 bytes

Variables Entered/Removed[a]

Nation	Model	Variables Entered	Variables Removed	Method
Korea	1	openness, neuroticism, agreeable, conscientious ness, extraversion[b]	.	Enter
Canada	1	openness, conscientious ness, extraversion, agreeable, neuroticism[b]	.	Enter

a. Dependent Variable: transformative

b. All requested variables entered.

Model Summary

Nation	Model	R	R Square	Adjusted R Square	Std. Error of the Estimate
Korea	1	.117[a]	.014	.009	1.13353
Canada	1	.180[b]	.032	.025	1.25511

a. Predictors: (Constant), openness, neuroticism, agreeable, conscientiousness, extraversion

b. Predictors: (Constant), openness, conscientiousness, extraversion, agreeable, neuroticism

ANOVA[a]

Nation	Model		Sum of Squares	df	Mean Square	F	Sig.
Korea	1	Regression	20.726	5	4.145	3.226	.007[b]
		Residual	1504.606	1171	1.285		
		Total	1525.332	1176			
Canada	1	Regression	34.451	5	6.890	4.374	.001[c]
		Residual	1030.254	654	1.575		
		Total	1064.704	659			

a. Dependent Variable: transformative

b. Predictors: (Constant), openness, neuroticism, agreeable, conscientiousness, extraversion

c. Predictors: (Constant), openness, conscientiousness, extraversion, agreeable, neuroticism

Coefficients[a]

Nation	Model		Unstandardized Coefficients B	Unstandardized Coefficients Std. Error	Standardized Coefficients Beta	t
Korea	1	(Constant)	3.180	.318		9.995
		extraversion	-.032	.054	-.020	-.596
		agreeable	.174	.060	.097	2.880
		conscientiousness	.094	.050	.058	1.896
		neuroticism	.091	.050	.055	1.840
		openness	-.083	.052	-.048	-1.580
Canada	1	(Constant)	3.504	.438		7.997
		extraversion	-.010	.054	-.008	-.193
		agreeable	.198	.065	.127	3.028
		conscientiousness	-.184	.062	-.123	-2.950
		neuroticism	.075	.058	.055	1.296
		openness	.072	.065	.044	1.105

Coefficients[a]

Nation	Model		Sig.	95.0% Confidence Interval for B Lower Bound	95.0% Confidence Interval for B Upper Bound	Correlations Zero-order
Korea	1	(Constant)	.000	2.556	3.804	
		extraversion	.551	-.137	.073	.024
		agreeable	.004	.055	.292	.082
		conscientiousness	.058	-.003	.192	.046
		neuroticism	.066	-.006	.189	.042
		openness	.114	-.185	.020	-.025
Canada	1	(Constant)	.000	2.644	4.364	
		extraversion	.847	-.116	.095	.002
		agreeable	.003	.070	.326	.097
		conscientiousness	.003	-.306	-.062	-.111
		neuroticism	.195	-.039	.190	.073
		openness	.270	-.056	.200	.058

Coefficients[a]

Nation	Model		Correlations Partial	Part	Collinearity Statistics Tolerance	VIF
Korea	1	(Constant)				
		extraversion	-.017	-.017	.739	1.353
		agreeable	.084	.084	.744	1.344
		conscientiousness	.055	.055	.914	1.094
		neuroticism	.054	.053	.953	1.049
		openness	-.046	-.046	.918	1.089
Canada	1	(Constant)				
		extraversion	-.008	-.007	.865	1.156
		agreeable	.118	.116	.847	1.180
		conscientiousness	-.115	-.113	.851	1.175
		neuroticism	.051	.050	.834	1.198
		openness	.043	.043	.934	1.070

a. Dependent Variable: transformative

Collinearity Diagnostics[a]

Nation	Model	Dimension	Eigenvalue	Condition Index	Variance Proportions (Constant)	extraversion
Korea	1	1	5.830	1.000	.00	.00
		2	.064	9.551	.00	.12
		3	.047	11.186	.01	.38
		4	.028	14.317	.00	.01
		5	.023	16.050	.00	.48
		6	.009	26.051	.99	.00
Canada	1	1	5.709	1.000	.00	.00
		2	.137	6.465	.00	.27
		3	.072	8.933	.00	.64
		4	.041	11.775	.00	.03
		5	.031	13.511	.00	.03
		6	.010	23.848	1.00	.03

Collinearity Diagnostics[a]

Nation	Model	Dimension	Variance Proportions agreeable	conscientiousness	neuroticism	openness
Korea	1	1	.00	.00	.00	.00
		2	.02	.02	.57	.00
		3	.03	.26	.05	.08
		4	.00	.36	.01	.77
		5	.82	.05	.02	.03
		6	.13	.31	.35	.12
Canada	1	1	.00	.00	.00	.00
		2	.00	.01	.33	.00
		3	.02	.18	.17	.02
		4	.02	.37	.06	.59
		5	.86	.12	.00	.23
		6	.09	.32	.43	.15

a. Dependent Variable: transformative

```
REGRESSION
  /MISSING LISTWISE
  /STATISTICS COEFF OUTS CI(95) R ANOVA COLLIN TOL ZPP
  /CRITERIA=PIN(.05) POUT(.10)
  /NOORIGIN
  /DEPENDENT fragility
  /METHOD=ENTER extraversion agreeable conscientiousness neuroticism openne
ss.
```

Regression

Notes

Output Created		20-MAY-2019 12:21:34
Comments		
Input	Data	C:\Users\1\Desktop\korea cnada study\merge korea canada\final.sav
	Active Dataset	DataSet1
	Filter	<none>
	Weight	<none>
	Split File	Nation
	N of Rows in Working Data File	1837
Missing Value Handling	Definition of Missing	User-defined missing values are treated as missing.
	Cases Used	Statistics are based on cases with no missing values for any variable used.
Syntax		REGRESSION /MISSING LISTWISE /STATISTICS COEFF OUTS CI(95) R ANOVA COLLIN TOL ZPP /CRITERIA=PIN(.05) POUT(.10) /NOORIGIN /DEPENDENT fragility /METHOD=ENTER extraversion agreeable conscientiousness neuroticism openness.
Resources	Processor Time	00:00:00.05
	Elapsed Time	00:00:00.04
	Memory Required	11408 bytes
	Additional Memory Required for Residual Plots	0 bytes

Variables Entered/Removed[a]

Nation	Model	Variables Entered	Variables Removed	Method
Korea	1	openness, neuroticism, agreeable, conscientiousness, extraversion[b]	.	Enter
Canada	1	openness, conscientiousness, extraversion, agreeable, neuroticism[b]	.	Enter

a. Dependent Variable: fragility

b. All requested variables entered.

Model Summary

Nation	Model	R	R Square	Adjusted R Square	Std. Error of the Estimate
Korea	1	.180[a]	.032	.028	1.04611
Canada	1	.329[b]	.108	.101	1.23220

a. Predictors: (Constant), openness, neuroticism, agreeable, conscientiousness, extraversion

b. Predictors: (Constant), openness, conscientiousness, extraversion, agreeable, neuroticism

ANOVA[a]

Nation	Model		Sum of Squares	df	Mean Square	F	Sig.
Korea	1	Regression	42.694	5	8.539	7.803	.000[b]
		Residual	1281.467	1171	1.094		
		Total	1324.162	1176			
Canada	1	Regression	120.284	5	24.057	15.844	.000[c]
		Residual	992.986	654	1.518		
		Total	1113.271	659			

a. Dependent Variable: fragility

b. Predictors: (Constant), openness, neuroticism, agreeable, conscientiousness, extraversion

c. Predictors: (Constant), openness, conscientiousness, extraversion, agreeable, neuroticism

Coefficients[a]

Nation	Model		Unstandardized Coefficients B	Unstandardized Coefficients Std. Error	Standardized Coefficients Beta	t
Korea	1	(Constant)	4.160	.294		14.169
		extraversion	-.154	.050	-.104	-3.114
		agreeable	.044	.056	.026	.787
		conscientiousness	.053	.046	.034	1.146
		neuroticism	.247	.046	.158	5.381
		openness	.060	.048	.037	1.248
Canada	1	(Constant)	4.505	.430		10.472
		extraversion	-.189	.053	-.142	-3.580
		agreeable	.012	.064	.008	.192
		conscientiousness	-.017	.061	-.011	-.272
		neuroticism	.354	.057	.250	6.190
		openness	-.072	.064	-.043	-1.127

Coefficients[a]

Nation	Model		Sig.	95.0% Confidence Interval for B Lower Bound	95.0% Confidence Interval for B Upper Bound	Correlations Zero-order
Korea	1	(Constant)	.000	3.584	4.736	
		extraversion	.002	-.252	-.057	-.081
		agreeable	.432	-.066	.153	-.020
		conscientiousness	.252	-.037	.142	-.002
		neuroticism	.000	.157	.337	.150
		openness	.212	-.034	.155	.018
Canada	1	(Constant)	.000	3.660	5.350	
		extraversion	.000	-.292	-.085	-.214
		agreeable	.848	-.114	.138	-.090
		conscientiousness	.786	-.137	.104	-.121
		neuroticism	.000	.241	.466	.293
		openness	.260	-.198	.054	-.086

Coefficients[a]

Nation	Model		Correlations: Partial	Correlations: Part	Collinearity Statistics: Tolerance	Collinearity Statistics: VIF
Korea	1	(Constant)				
		extraversion	-.091	-.090	.739	1.353
		agreeable	.023	.023	.744	1.344
		conscientiousness	.033	.033	.914	1.094
		neuroticism	.155	.155	.953	1.049
		openness	.036	.036	.918	1.089
Canada	1	(Constant)				
		extraversion	-.139	-.132	.865	1.156
		agreeable	.008	.007	.847	1.180
		conscientiousness	-.011	-.010	.851	1.175
		neuroticism	.235	.229	.834	1.198
		openness	-.044	-.042	.934	1.070

a. Dependent Variable: fragility

Collinearity Diagnostics[a]

Nation	Model	Dimension	Eigenvalue	Condition Index	Variance Proportions: (Constant)	Variance Proportions: extraversion
Korea	1	1	5.830	1.000	.00	.00
		2	.064	9.551	.00	.12
		3	.047	11.186	.01	.38
		4	.028	14.317	.00	.01
		5	.023	16.050	.00	.48
		6	.009	26.051	.99	.00
Canada	1	1	5.709	1.000	.00	.00
		2	.137	6.465	.00	.27
		3	.072	8.933	.00	.64
		4	.041	11.775	.00	.03
		5	.031	13.511	.00	.03
		6	.010	23.848	1.00	.03

Collinearity Diagnostics[a]

Nation	Model	Dimension	Variance Proportions agreeable	conscientiousness	neuroticism	openness
Korea	1	1	.00	.00	.00	.00
		2	.02	.02	.57	.00
		3	.03	.26	.05	.08
		4	.00	.36	.01	.77
		5	.82	.05	.02	.03
		6	.13	.31	.35	.12
Canada	1	1	.00	.00	.00	.00
		2	.00	.01	.33	.00
		3	.02	.18	.17	.02
		4	.02	.37	.06	.59
		5	.86	.12	.00	.23
		6	.09	.32	.43	.15

a. Dependent Variable: fragility

```
REGRESSION
  /MISSING LISTWISE
  /STATISTICS COEFF OUTS CI(95) R ANOVA COLLIN TOL ZPP
  /CRITERIA=PIN(.05) POUT(.10)
  /NOORIGIN
  /DEPENDENT valuing
  /METHOD=ENTER extraversion agreeable conscientiousness neuroticism openne
ss.
```

Regression

Notes

Output Created		20-MAY-2019 12:21:41
Comments		
Input	Data	C:\Users\1\Desktop\korea cnada study\merge korea canada\final.sav
	Active Dataset	DataSet1
	Filter	<none>
	Weight	<none>
	Split File	Nation
	N of Rows in Working Data File	1837
Missing Value Handling	Definition of Missing	User-defined missing values are treated as missing.
	Cases Used	Statistics are based on cases with no missing values for any variable used.
Syntax		REGRESSION<br>/MISSING LISTWISE<br>/STATISTICS COEFF OUTS CI(95) R ANOVA COLLIN TOL ZPP<br>/CRITERIA=PIN(.05) POUT(.10)<br>/NOORIGIN<br>/DEPENDENT valuing<br>/METHOD=ENTER extraversion agreeable conscientiousness neuroticism openness.
Resources	Processor Time	00:00:00.02
	Elapsed Time	00:00:00.03
	Memory Required	11408 bytes
	Additional Memory Required for Residual Plots	0 bytes

Variables Entered/Removed[a]

Nation	Model	Variables Entered	Variables Removed	Method
Korea	1	openness, neuroticism, agreeable, conscientiousness, extraversion[b]	.	Enter
Canada	1	openness, conscientiousness, extraversion, agreeable, neuroticism[b]	.	Enter

a. Dependent Variable: valuing

b. All requested variables entered.

Model Summary

Nation	Model	R	R Square	Adjusted R Square	Std. Error of the Estimate
Korea	1	.262[a]	.069	.065	.69078
Canada	1	.388[b]	.151	.144	.91045

a. Predictors: (Constant), openness, neuroticism, agreeable, conscientiousness, extraversion

b. Predictors: (Constant), openness, conscientiousness, extraversion, agreeable, neuroticism

ANOVA[a]

Nation	Model		Sum of Squares	df	Mean Square	F	Sig.
Korea	1	Regression	41.279	5	8.256	17.301	.000[b]
		Residual	558.774	1171	.477		
		Total	600.053	1176			
Canada	1	Regression	96.365	5	19.273	23.251	.000[c]
		Residual	542.119	654	.829		
		Total	638.484	659			

a. Dependent Variable: valuing

b. Predictors: (Constant), openness, neuroticism, agreeable, conscientiousness, extraversion

c. Predictors: (Constant), openness, conscientiousness, extraversion, agreeable, neuroticism

Coefficients[a]

Nation	Model		Unstandardized Coefficients B	Unstandardized Coefficients Std. Error	Standardized Coefficients Beta	t
Korea	1	(Constant)	3.677	.194		18.963
		extraversion	.019	.033	.019	.566
		agreeable	-.013	.037	-.012	-.354
		conscientiousness	.082	.030	.080	2.726
		neuroticism	.278	.030	.265	9.181
		openness	-.030	.032	-.028	-.944
Canada	1	(Constant)	3.804	.318		11.966
		extraversion	.037	.039	.037	.947
		agreeable	-.084	.047	-.069	-1.774
		conscientiousness	-.071	.045	-.062	-1.579
		neuroticism	.367	.042	.343	8.703
		openness	-.062	.047	-.049	-1.316

Coefficients[a]

Nation	Model		Sig.	95.0% Confidence Interval for B Lower Bound	95.0% Confidence Interval for B Upper Bound	Correlations Zero-order
Korea	1	(Constant)	.000	3.296	4.057	
		extraversion	.572	-.046	.083	.013
		agreeable	.723	-.085	.059	-.008
		conscientiousness	.007	.023	.142	.019
		neuroticism	.000	.218	.337	.250
		openness	.345	-.093	.032	-.026
Canada	1	(Constant)	.000	3.179	4.428	
		extraversion	.344	-.040	.113	-.090
		agreeable	.077	-.177	.009	-.146
		conscientiousness	.115	-.160	.017	-.194
		neuroticism	.000	.284	.450	.372
		openness	.189	-.155	.031	-.093

Coefficients[a]

Nation	Model		Correlations Partial	Correlations Part	Collinearity Statistics Tolerance	Collinearity Statistics VIF
Korea	1	(Constant)				
		extraversion	.017	.016	.739	1.353
		agreeable	-.010	-.010	.744	1.344
		conscientiousness	.079	.077	.914	1.094
		neuroticism	.259	.259	.953	1.049
		openness	-.028	-.027	.918	1.089
Canada	1	(Constant)				
		extraversion	.037	.034	.865	1.156
		agreeable	-.069	-.064	.847	1.180
		conscientiousness	-.062	-.057	.851	1.175
		neuroticism	.322	.314	.834	1.198
		openness	-.051	-.047	.934	1.070

a. Dependent Variable: valuing

Collinearity Diagnostics[a]

Nation	Model	Dimension	Eigenvalue	Condition Index	Variance Proportions (Constant)	Variance Proportions extraversion
Korea	1	1	5.830	1.000	.00	.00
		2	.064	9.551	.00	.12
		3	.047	11.186	.01	.38
		4	.028	14.317	.00	.01
		5	.023	16.050	.00	.48
		6	.009	26.051	.99	.00
Canada	1	1	5.709	1.000	.00	.00
		2	.137	6.465	.00	.27
		3	.072	8.933	.00	.64
		4	.041	11.775	.00	.03
		5	.031	13.511	.00	.03
		6	.010	23.848	1.00	.03

Collinearity Diagnostics[a]

Nation	Model	Dimension	Variance Proportions agreeable	conscientiousness	neuroticism	openness
Korea	1	1	.00	.00	.00	.00
		2	.02	.02	.57	.00
		3	.03	.26	.05	.08
		4	.00	.36	.01	.77
		5	.82	.05	.02	.03
		6	.13	.31	.35	.12
Canada	1	1	.00	.00	.00	.00
		2	.00	.01	.33	.00
		3	.02	.18	.17	.02
		4	.02	.37	.06	.59
		5	.86	.12	.00	.23
		6	.09	.32	.43	.15

a. Dependent Variable: valuing

```
REGRESSION
  /MISSING LISTWISE
  /STATISTICS COEFF OUTS CI(95) R ANOVA COLLIN TOL ZPP
  /CRITERIA=PIN(.05) POUT(.10)
  /NOORIGIN
  /DEPENDENT inflexibility
  /METHOD=ENTER extraversion agreeable conscientiousness neuroticism openne
ss.
```

Regression

Notes

Output Created		20-MAY-2019 12:21:47
Comments		
Input	Data	C:\Users\1\Desktop\korea cnada study\merge korea canada\final.sav
	Active Dataset	DataSet1
	Filter	<none>
	Weight	<none>
	Split File	Nation
	N of Rows in Working Data File	1837
Missing Value Handling	Definition of Missing	User-defined missing values are treated as missing.
	Cases Used	Statistics are based on cases with no missing values for any variable used.
Syntax		REGRESSION /MISSING LISTWISE /STATISTICS COEFF OUTS CI(95) R ANOVA COLLIN TOL ZPP /CRITERIA=PIN(.05) POUT(.10) /NOORIGIN /DEPENDENT inflexibility /METHOD=ENTER extraversion agreeable conscientiousness neuroticism openness.
Resources	Processor Time	00:00:00.02
	Elapsed Time	00:00:00.03
	Memory Required	11408 bytes
	Additional Memory Required for Residual Plots	0 bytes

Variables Entered/Removed[a]

Nation	Model	Variables Entered	Variables Removed	Method
Korea	1	openness, neuroticism, agreeable, conscientiousness, extraversion[b]	.	Enter
Canada	1	openness, conscientiousness, extraversion, agreeable, neuroticism[b]	.	Enter

a. Dependent Variable: inflexibility

b. All requested variables entered.

Model Summary

Nation	Model	R	R Square	Adjusted R Square	Std. Error of the Estimate
Korea	1	.148[a]	.022	.018	1.05836
Canada	1	.267[b]	.071	.064	1.21450

a. Predictors: (Constant), openness, neuroticism, agreeable, conscientiousness, extraversion

b. Predictors: (Constant), openness, conscientiousness, extraversion, agreeable, neuroticism

ANOVA[a]

Nation	Model		Sum of Squares	df	Mean Square	F	Sig.
Korea	1	Regression	29.538	5	5.908	5.274	.000[b]
		Residual	1311.668	1171	1.120		
		Total	1341.206	1176			
Canada	1	Regression	73.825	5	14.765	10.010	.000[c]
		Residual	964.658	654	1.475		
		Total	1038.483	659			

a. Dependent Variable: inflexibility

b. Predictors: (Constant), openness, neuroticism, agreeable, conscientiousness, extraversion

c. Predictors: (Constant), openness, conscientiousness, extraversion, agreeable, neuroticism

Coefficients[a]

Nation	Model		Unstandardized Coefficients B	Unstandardized Coefficients Std. Error	Standardized Coefficients Beta	t
Korea	1	(Constant)	4.788	.297		16.120
		extraversion	-.027	.050	-.018	-.530
		agreeable	-.224	.056	-.133	-3.978
		conscientiousness	-.026	.046	-.017	-.568
		neuroticism	-.013	.046	-.008	-.275
		openness	-.016	.049	-.010	-.326
Canada	1	(Constant)	5.408	.424		12.755
		extraversion	-.015	.052	-.012	-.291
		agreeable	-.334	.063	-.216	-5.281
		conscientiousness	-.116	.060	-.078	-1.917
		neuroticism	-.144	.056	-.106	-2.565
		openness	-.113	.063	-.070	-1.785

Coefficients[a]

Nation	Model		Sig.	95.0% Confidence Interval for B Lower Bound	95.0% Confidence Interval for B Upper Bound	Correlations Zero-order
Korea	1	(Constant)	.000	4.206	5.371	
		extraversion	.596	-.125	.072	-.088
		agreeable	.000	-.335	-.114	-.146
		conscientiousness	.570	-.117	.065	-.035
		neuroticism	.783	-.104	.078	.001
		openness	.744	-.112	.080	-.043
Canada	1	(Constant)	.000	4.576	6.241	
		extraversion	.771	-.117	.087	-.070
		agreeable	.000	-.458	-.210	-.235
		conscientiousness	.056	-.234	.003	-.099
		neuroticism	.011	-.255	-.034	-.031
		openness	.075	-.237	.011	-.120

Coefficients[a]

Nation	Model		Correlations: Partial	Correlations: Part	Collinearity Statistics: Tolerance	Collinearity Statistics: VIF
Korea	1	(Constant)				
		extraversion	-.015	-.015	.739	1.353
		agreeable	-.115	-.115	.744	1.344
		conscientiousness	-.017	-.016	.914	1.094
		neuroticism	-.008	-.008	.953	1.049
		openness	-.010	-.009	.918	1.089
Canada	1	(Constant)				
		extraversion	-.011	-.011	.865	1.156
		agreeable	-.202	-.199	.847	1.180
		conscientiousness	-.075	-.072	.851	1.175
		neuroticism	-.100	-.097	.834	1.198
		openness	-.070	-.067	.934	1.070

a. Dependent Variable: inflexibility

Collinearity Diagnostics[a]

Nation	Model	Dimension	Eigenvalue	Condition Index	Variance Proportions: (Constant)	Variance Proportions: extraversion
Korea	1	1	5.830	1.000	.00	.00
		2	.064	9.551	.00	.12
		3	.047	11.186	.01	.38
		4	.028	14.317	.00	.01
		5	.023	16.050	.00	.48
		6	.009	26.051	.99	.00
Canada	1	1	5.709	1.000	.00	.00
		2	.137	6.465	.00	.27
		3	.072	8.933	.00	.64
		4	.041	11.775	.00	.03
		5	.031	13.511	.00	.03
		6	.010	23.848	1.00	.03

Collinearity Diagnostics[a]

Nation	Model	Dimension	Variance Proportions agreeable	conscientiousness	neuroticism	openness
Korea	1	1	.00	.00	.00	.00
		2	.02	.02	.57	.00
		3	.03	.26	.05	.08
		4	.00	.36	.01	.77
		5	.82	.05	.02	.03
		6	.13	.31	.35	.12
Canada	1	1	.00	.00	.00	.00
		2	.00	.01	.33	.00
		3	.02	.18	.17	.02
		4	.02	.37	.06	.59
		5	.86	.12	.00	.23
		6	.09	.32	.43	.15

a. Dependent Variable: inflexibility

```
REGRESSION
  /MISSING LISTWISE
  /STATISTICS COEFF OUTS CI(95) R ANOVA COLLIN TOL CHANGE ZPP
  /CRITERIA=PIN(.05) POUT(.10)
  /NOORIGIN
  /DEPENDENT social_well_being
  /METHOD=ENTER age sex extraversion agreeable conscientiousness neuroticis
m openness
  /METHOD=ENTER eudaimonism inclusive externality fear transformative fragi
lity valuing
    inflexibility.
```

Regression

Notes

Output Created		20-MAY-2019 12:22:47
Comments		
Input	Data	C:\Users\1\Desktop\korea cnada study\merge korea canada\final.sav
	Active Dataset	DataSet1
	Filter	<none>
	Weight	<none>
	Split File	Nation
	N of Rows in Working Data File	1837
Missing Value Handling	Definition of Missing	User-defined missing values are treated as missing.
	Cases Used	Statistics are based on cases with no missing values for any variable used.
Syntax		REGRESSION /MISSING LISTWISE /STATISTICS COEFF OUTS CI(95) R ANOVA COLLIN TOL CHANGE ZPP /CRITERIA=PIN(.05) POUT(.10) /NOORIGIN /DEPENDENT social_well_being /METHOD=ENTER age sex extraversion agreeable conscientiousness neuroticism openness /METHOD=ENTER eudaimonism inclusive externality fear transformative fragility valuing inflexibility.
Resources	Processor Time	00:00:00.03
	Elapsed Time	00:00:00.03
	Memory Required	21520 bytes
	Additional Memory Required for Residual Plots	0 bytes

Variables Entered/Removed[a]

Nation	Model	Variables Entered	Variables Removed	Method
Korea	1	openness, age, sex, extraversion, neuroticism, conscientiousness, agreeable[b]	.	Enter
	2	inflexibility, transformative, inclusive, eudaimonism, fragility, valuing, fear, externality[b]	.	Enter
Canada	1	openness, sex, conscientiousness, extraversion, age, agreeable, neuroticism[b]	.	Enter
	2	transformative, inclusive, eudaimonism, inflexibility, fragility, valuing, fear, externality[b]	.	Enter

a. Dependent Variable: social_well_being

b. All requested variables entered.

Model Summary

Nation	Model	R	R Square	Adjusted R Square	Std. Error of the Estimate	Change ... R Square Change
Korea	1	.423[a]	.179	.174	.86110	.179
	2	.519[b]	.269	.260	.81532	.090
Canada	1	.493[c]	.243	.235	1.00483	.243
	2	.547[d]	.299	.283	.97253	.057

Model Summary

Nation	Model	Change Statistics F Change	df1	df2	Sig. F Change
Korea	1	36.431	7	1169	.000
	2	17.872	8	1161	.000
Canada	1	29.859	7	652	.000
	2	6.503	8	644	.000

a. Predictors: (Constant), openness, age, sex, extraversion, neuroticism, conscientiousness, agreeable

b. Predictors: (Constant), openness, age, sex, extraversion, neuroticism, conscientiousness, agreeable, inflexibility, transformative, inclusive, eudaimonism, fragility, valuing, fear, externality

c. Predictors: (Constant), openness, sex, conscientiousness, extraversion, age, agreeable, neuroticism

d. Predictors: (Constant), openness, sex, conscientiousness, extraversion, age, agreeable, neuroticism, transformative, inclusive, eudaimonism, inflexibility, fragility, valuing, fear, externality

ANOVA[a]

Nation	Model		Sum of Squares	df	Mean Square	F	Sig.
Korea	1	Regression	189.095	7	27.014	36.431	.000[b]
		Residual	866.812	1169	.741		
		Total	1055.907	1176			
	2	Regression	284.136	15	18.942	28.496	.000[c]
		Residual	771.772	1161	.665		
		Total	1055.907	1176			
Canada	1	Regression	211.038	7	30.148	29.859	.000[d]
		Residual	658.313	652	1.010		
		Total	869.352	659			
	2	Regression	260.245	15	17.350	18.344	.000[e]
		Residual	609.107	644	.946		
		Total	869.352	659			

a. Dependent Variable: social_well_being

b. Predictors: (Constant), openness, age, sex, extraversion, neuroticism, conscientiousness, agreeable

c. Predictors: (Constant), openness, age, sex, extraversion, neuroticism, conscientiousness, agreeable, inflexibility, transformative, inclusive, eudaimonism, fragility, valuing, fear, externality

d. Predictors: (Constant), openness, sex, conscientiousness, extraversion, age, agreeable, neuroticism

e. Predictors: (Constant), openness, sex, conscientiousness, extraversion, age, agreeable, neuroticism, transformative, inclusive, eudaimonism, inflexibility, fragility, valuing, fear, externality

Coefficients[a]

Nation	Model		Unstandardized Coefficients B	Unstandardized Coefficients Std. Error	Standardized Coefficients Beta	t
Korea	1	(Constant)	.361	.266		1.355
		age	.005	.002	.062	2.242
		sex	-.107	.052	-.056	-2.066
		extraversion	.316	.041	.239	7.738
		agreeable	.180	.046	.120	3.883
		conscientiousness	.052	.039	.038	1.353
		neuroticism	-.272	.039	-.195	-7.007
		openness	.100	.040	.070	2.504
	2	(Constant)	.360	.314		1.146
		age	.001	.002	.011	.415
		sex	-.131	.050	-.069	-2.619
		extraversion	.284	.039	.214	7.285
		agreeable	.121	.045	.081	2.697
		conscientiousness	.041	.037	.030	1.106
		neuroticism	-.216	.039	-.156	-5.506
		openness	.088	.038	.061	2.315
		eudaimonism	.008	.005	.042	1.601
		inclusive	.178	.022	.212	8.055
		externality	-.062	.028	-.069	-2.235
		fear	-.053	.027	-.061	-1.961
		transformative	.082	.024	.098	3.376
		fragility	-.112	.024	-.125	-4.597
		valuing	.083	.037	.063	2.235
		inflexibility	-.015	.024	-.017	-.627
Canada	1	(Constant)	.804	.398		2.017
		age	.003	.003	.041	1.124
		sex	-.087	.087	-.037	-1.003
		extraversion	.197	.043	.168	4.589
		agreeable	.242	.055	.172	4.425
		conscientiousness	-.013	.050	-.009	-.253
		neuroticism	-.370	.049	-.297	-7.606
		openness	.139	.053	.094	2.641
	2	(Constant)	.437	.471		.927
		age	.004	.003	.054	1.498
		sex	-.140	.085	-.059	-1.653
		extraversion	.139	.043	.118	3.210
		agreeable	.198	.056	.140	3.560

Coefficients[a]

Nation	Model		Sig.	95.0% Confidence Interval for B Lower Bound	Upper Bound	Correlations Zero-order
Korea	1	(Constant)	.176	-.162	.883	
		age	.025	.001	.009	.087
		sex	.039	-.209	-.005	-.077
		extraversion	.000	.236	.396	.315
		agreeable	.000	.089	.271	.253
		conscientiousness	.176	-.023	.128	.154
		neuroticism	.000	-.348	-.196	-.238
		openness	.012	.022	.178	.163
	2	(Constant)	.252	-.257	.977	
		age	.678	-.003	.005	.087
		sex	.009	-.229	-.033	-.077
		extraversion	.000	.207	.360	.315
		agreeable	.007	.033	.208	.253
		conscientiousness	.269	-.031	.113	.154
		neuroticism	.000	-.293	-.139	-.238
		openness	.021	.013	.163	.163
		eudaimonism	.110	-.002	.017	.121
		inclusive	.000	.135	.221	.296
		externality	.026	-.116	-.008	-.211
		fear	.050	-.107	.000	-.135
		transformative	.001	.034	.129	.103
		fragility	.000	-.160	-.064	-.170
		valuing	.026	.010	.156	-.002
		inflexibility	.531	-.062	.032	-.083
Canada	1	(Constant)	.044	.021	1.586	
		age	.261	-.002	.008	.173
		sex	.316	-.257	.083	-.037
		extraversion	.000	.113	.282	.310
		agreeable	.000	.135	.350	.290
		conscientiousness	.800	-.111	.086	.174
		neuroticism	.000	-.466	-.275	-.394
		openness	.008	.036	.242	.184
	2	(Constant)	.354	-.489	1.362	
		age	.135	-.001	.009	.173
		sex	.099	-.307	.026	-.037
		extraversion	.001	.054	.224	.310
		agreeable	.000	.089	.307	.290

Coefficients[a]

Nation	Model		Correlations Partial	Correlations Part	Collinearity Statistics Tolerance	Collinearity Statistics VIF
Korea	1	(Constant)				
		age	.065	.059	.920	1.086
		sex	-.060	-.055	.941	1.063
		extraversion	.221	.205	.739	1.354
		agreeable	.113	.103	.730	1.370
		conscientiousness	.040	.036	.876	1.142
		neuroticism	-.201	-.186	.903	1.108
		openness	.073	.066	.908	1.101
	2	(Constant)				
		age	.012	.010	.871	1.148
		sex	-.077	-.066	.908	1.101
		extraversion	.209	.183	.729	1.372
		agreeable	.079	.068	.702	1.424
		conscientiousness	.032	.028	.861	1.161
		neuroticism	-.160	-.138	.789	1.267
		openness	.068	.058	.894	1.118
		eudaimonism	.047	.040	.917	1.090
		inclusive	.230	.202	.911	1.097
		externality	-.065	-.056	.655	1.526
		fear	-.057	-.049	.656	1.526
		transformative	.099	.085	.742	1.347
		fragility	-.134	-.115	.845	1.183
		valuing	.065	.056	.803	1.246
		inflexibility	-.018	-.016	.853	1.172
Canada	1	(Constant)				
		age	.044	.038	.862	1.159
		sex	-.039	-.034	.876	1.142
		extraversion	.177	.156	.864	1.157
		agreeable	.171	.151	.772	1.296
		conscientiousness	-.010	-.009	.848	1.180
		neuroticism	-.285	-.259	.764	1.310
		openness	.103	.090	.922	1.085
	2	(Constant)				
		age	.059	.049	.829	1.206
		sex	-.065	-.055	.852	1.174
		extraversion	.125	.106	.801	1.249
		agreeable	.139	.117	.701	1.426

Coefficients[a]

Nation	Model		Unstandardized Coefficients B	Unstandardized Coefficients Std. Error	Standardized Coefficients Beta	t
		conscientiousness	-.005	.049	-.004	-.097
		neuroticism	-.314	.052	-.251	-6.082
		openness	.095	.052	.064	1.815
		eudaimonism	.021	.007	.098	2.838
		inclusive	.092	.031	.105	2.950
		externality	-.029	.039	-.033	-.728
		fear	-.112	.037	-.136	-2.994
		transformative	.038	.033	.042	1.156
		fragility	-.090	.034	-.102	-2.671
		valuing	.116	.047	.099	2.479
		inflexibility	.074	.036	.081	2.078

Coefficients[a]

Nation	Model		Sig.	95.0% Confidence Interval for B Lower Bound	95.0% Confidence Interval for B Upper Bound	Correlations Zero-order
		conscientiousness	.923	-.101	.092	.174
		neuroticism	.000	-.415	-.212	-.394
		openness	.070	-.008	.197	.184
		eudaimonism	.005	.006	.035	.176
		inclusive	.003	.031	.153	.202
		externality	.467	-.106	.049	-.214
		fear	.003	-.185	-.039	-.288
		transformative	.248	-.027	.103	.009
		fragility	.008	-.156	-.024	-.249
		valuing	.013	.024	.208	-.104
		inflexibility	.038	.004	.144	.003

Coefficients[a]

Nation	Model		Correlations: Partial	Correlations: Part	Collinearity Statistics: Tolerance	Collinearity Statistics: VIF
		conscientiousness	-.004	-.003	.826	1.211
		neuroticism	-.233	-.201	.637	1.569
		openness	.071	.060	.876	1.142
		eudaimonism	.111	.094	.904	1.106
		inclusive	.115	.097	.856	1.168
		externality	-.029	-.024	.529	1.889
		fear	-.117	-.099	.530	1.888
		transformative	.046	.038	.807	1.238
		fragility	-.105	-.088	.746	1.341
		valuing	.097	.082	.676	1.480
		inflexibility	.082	.069	.713	1.402

a. Dependent Variable: social_well_being

Excluded Variables[a]

Nation	Model		Beta In	t	Sig.	Partial Correlation	Collinearity . Tolerance
Korea	1	eudaimonism	.073[b]	2.686	.007	.078	.956
		inclusive	.246[b]	9.429	.000	.266	.961
		externality	-.114[b]	-4.141	.000	-.120	.917
		fear	-.060[b]	-2.205	.028	-.064	.937
		transformative	.092[b]	3.426	.001	.100	.972
		fragility	-.120[b]	-4.486	.000	-.130	.966
		valuing	.041[b]	1.467	.143	.043	.906
		inflexibility	-.048[b]	-1.794	.073	-.052	.973
Canada	1	eudaimonism	.105[c]	2.979	.003	.116	.925
		inclusive	.139[c]	3.992	.000	.155	.936
		externality	-.043[c]	-1.140	.255	-.045	.832
		fear	-.105[c]	-2.753	.006	-.107	.790
		transformative	.017[c]	.487	.626	.019	.948
		fragility	-.114[c]	-3.175	.002	-.123	.889
		valuing	.072[c]	1.930	.054	.075	.842
		inflexibility	.053[c]	1.482	.139	.058	.918

Excluded Variables[a]

Nation	Model		Collinearity Statistics	
			VIF	Minimum Tolerance
Korea	1	eudaimonism	1.045	.729
		inclusive	1.040	.725
		externality	1.090	.726
		fear	1.067	.725
		transformative	1.029	.723
		fragility	1.035	.730
		valuing	1.104	.730
		inflexibility	1.028	.721
Canada	1	eudaimonism	1.081	.754
		inclusive	1.068	.764
		externality	1.202	.735
		fear	1.266	.720
		transformative	1.055	.759
		fragility	1.125	.722
		valuing	1.188	.693
		inflexibility	1.089	.744

a. Dependent Variable: social_well_being

b. Predictors in the Model: (Constant), openness, age, sex, extraversion, neuroticism, conscientiousness, agreeable

c. Predictors in the Model: (Constant), openness, sex, conscientiousness, extraversion, age, agreeable,

Collinearity Diagnostics[a]

Nation	Model	Dimension	Eigenvalue	Condition Index	Variance Proportions (Constant)	age	sex
Korea	1	1	7.661	1.000	.00	.00	.00
		2	.105	8.547	.00	.22	.46
		3	.080	9.769	.00	.39	.22
		4	.057	11.572	.00	.00	.24
		5	.040	13.817	.00	.18	.02
		6	.027	16.936	.00	.08	.01
		7	.022	18.558	.00	.00	.02
		8	.007	32.008	.99	.12	.03
	2	1	15.013	1.000	.00	.00	.00
		2	.194	8.799	.00	.01	.01
		3	.132	10.681	.00	.03	.20
		4	.109	11.763	.00	.02	.02
		5	.095	12.593	.00	.12	.17
		6	.087	13.142	.00	.05	.23
		7	.075	14.174	.00	.33	.11
		8	.057	16.243	.00	.15	.02
		9	.054	16.706	.00	.06	.11
		10	.044	18.477	.00	.00	.00
		11	.041	19.092	.00	.05	.05
		12	.030	22.306	.00	.03	.00
		13	.026	23.802	.00	.09	.00
		14	.022	26.408	.00	.00	.01
		15	.017	29.438	.00	.02	.03
		16	.005	54.024	.99	.04	.04
Canada	1	1	7.555	1.000	.00	.00	.00
		2	.149	7.123	.00	.05	.04
		3	.095	8.928	.00	.34	.06
		4	.070	10.412	.00	.01	.51
		5	.055	11.677	.00	.34	.16
		6	.040	13.690	.00	.04	.01
		7	.028	16.433	.00	.03	.16
		8	.008	30.436	1.00	.20	.05
	2	1	14.609	1.000	.00	.00	.00
		2	.396	6.072	.00	.00	.00
		3	.173	9.178	.00	.04	.02
		4	.131	10.545	.00	.07	.00
		5	.107	11.702	.00	.00	.11

Collinearity Diagnostics[a]

Nation	Model	Dimension	Variance Proportions extraversion	agreeable	conscientiousness	neuroticism
Korea	1	1	.00	.00	.00	.00
		2	.00	.00	.02	.04
		3	.13	.03	.00	.00
		4	.09	.02	.00	.56
		5	.32	.01	.20	.08
		6	.00	.00	.56	.02
		7	.46	.83	.05	.01
		8	.00	.11	.17	.30
	2	1	.00	.00	.00	.00
		2	.01	.00	.00	.00
		3	.01	.01	.00	.02
		4	.00	.00	.01	.00
		5	.00	.00	.01	.01
		6	.06	.02	.00	.00
		7	.04	.00	.00	.00
		8	.12	.01	.00	.02
		9	.05	.00	.00	.17
		10	.01	.01	.01	.24
		11	.14	.00	.14	.19
		12	.09	.00	.15	.04
		13	.01	.00	.49	.01
		14	.46	.81	.03	.00
		15	.00	.04	.07	.21
		16	.00	.08	.09	.07
Canada	1	1	.00	.00	.00	.00
		2	.16	.00	.01	.25
		3	.45	.00	.02	.00
		4	.21	.02	.02	.26
		5	.11	.01	.16	.02
		6	.03	.03	.54	.06
		7	.02	.92	.02	.02
		8	.03	.02	.22	.40
	2	1	.00	.00	.00	.00
		2	.03	.00	.00	.00
		3	.02	.00	.00	.05
		4	.01	.00	.02	.00
		5	.02	.00	.00	.10

Collinearity Diagnostics[a]

Nation	Model	Dimension	Variance Proportions openness	eudaimonism	inclusive	externality	fear
Korea	1	1	.00				
		2	.00				
		3	.02				
		4	.00				
		5	.22				
		6	.58				
		7	.03				
		8	.14				
	2	1	.00	.00	.00	.00	.00
		2	.00	.04	.07	.10	.14
		3	.00	.14	.08	.01	.09
		4	.00	.21	.68	.00	.01
		5	.00	.12	.00	.02	.07
		6	.01	.22	.01	.07	.03
		7	.01	.20	.06	.10	.00
		8	.00	.01	.00	.03	.23
		9	.00	.00	.02	.10	.11
		10	.02	.00	.00	.49	.22
		11	.14	.01	.00	.00	.07
		12	.11	.01	.02	.02	.00
		13	.58	.00	.00	.03	.01
		14	.03	.00	.01	.01	.01
		15	.00	.00	.02	.01	.01
		16	.08	.03	.01	.00	.00
Canada	1	1	.00				
		2	.00				
		3	.00				
		4	.00				
		5	.23				
		6	.40				
		7	.18				
		8	.17				
	2	1	.00	.00	.00	.00	.00
		2	.00	.01	.01	.04	.12
		3	.00	.01	.01	.01	.01
		4	.00	.01	.65	.00	.00
		5	.00	.15	.00	.04	.24

Collinearity Diagnostics[a]

Nation	Model	Dimension	Variance Proportions transformative	fragility	valuing	inflexibility
Korea	1	1				
		2				
		3				
		4				
		5				
		6				
		7				
		8				
	2	1	.00	.00	.00	.00
		2	.00	.00	.00	.01
		3	.03	.00	.00	.00
		4	.00	.00	.00	.00
		5	.05	.00	.00	.16
		6	.06	.01	.00	.03
		7	.10	.01	.00	.00
		8	.12	.08	.00	.22
		9	.02	.04	.01	.39
		10	.25	.01	.00	.05
		11	.12	.10	.00	.03
		12	.19	.63	.00	.01
		13	.03	.00	.02	.00
		14	.02	.02	.01	.00
		15	.00	.03	.84	.02
		16	.00	.05	.11	.07
Canada	1	1				
		2				
		3				
		4				
		5				
		6				
		7				
		8				
	2	1	.00	.00	.00	.00
		2	.00	.00	.00	.02
		3	.03	.01	.00	.31
		4	.00	.01	.00	.00
		5	.10	.00	.01	.01

Collinearity Diagnostics[a]

Nation	Model	Dimension	Eigenvalue	Condition Index	Variance Proportions (Constant)	age	sex
		6	.097	12.289	.00	.00	.00
		7	.086	13.039	.00	.21	.09
		8	.073	14.174	.00	.01	.07
		9	.070	14.491	.00	.10	.10
		10	.061	15.481	.00	.00	.33
		11	.051	16.871	.00	.35	.02
		12	.044	18.168	.00	.00	.03
		13	.038	19.549	.00	.00	.01
		14	.031	21.685	.00	.01	.03
		15	.026	23.599	.00	.05	.12
		16	.006	50.079	1.00	.15	.06

Collinearity Diagnostics[a]

Nation	Model	Dimension	Variance Proportions extraversion	agreeable	conscientiousness	neuroticism
		6	.38	.00	.00	.00
		7	.13	.00	.01	.01
		8	.17	.00	.00	.06
		9	.06	.00	.00	.01
		10	.01	.01	.00	.19
		11	.04	.00	.35	.05
		12	.05	.01	.00	.08
		13	.00	.01	.28	.03
		14	.03	.00	.10	.27
		15	.03	.94	.04	.00
		16	.03	.02	.20	.14

Collinearity Diagnostics[a]

Nation	Model	Dimension	Variance Proportions openness	eudaimonism	inclusive	externality	fear
		6	.00	.40	.01	.01	.08
		7	.00	.02	.20	.06	.00
		8	.01	.17	.00	.06	.02
		9	.00	.00	.03	.33	.44
		10	.01	.00	.00	.36	.05
		11	.08	.05	.00	.00	.02
		12	.22	.12	.02	.03	.00
		13	.39	.03	.00	.00	.00
		14	.07	.00	.07	.05	.00
		15	.10	.01	.00	.01	.02
		16	.12	.03	.00	.00	.00

Collinearity Diagnostics[a]

Nation	Model	Dimension	Variance Proportions transformative	fragility	valuing	inflexibility
		6	.00	.00	.00	.06
		7	.00	.02	.00	.28
		8	.38	.03	.00	.08
		9	.16	.03	.01	.00
		10	.00	.06	.02	.14
		11	.08	.03	.00	.00
		12	.13	.55	.00	.00
		13	.05	.21	.12	.00
		14	.01	.02	.75	.00
		15	.03	.00	.01	.03
		16	.02	.02	.06	.06

a. Dependent Variable: social_well_being

```
REGRESSION
 /MISSING LISTWISE
 /STATISTICS COEFF OUTS CI(95) R ANOVA COLLIN TOL CHANGE ZPP
 /CRITERIA=PIN(.05) POUT(.10)
 /NOORIGIN
 /DEPENDENT psychological_well_being
 /METHOD=ENTER age sex extraversion agreeable conscientiousness neuroticis
```

```
m openness
  /METHOD=ENTER eudaimonism inclusive externality fear transformative fragi
lity valuing
    inflexibility.
```

Regression

Notes

Output Created		20-MAY-2019 12:22:55
Comments		
Input	Data	C:\Users\1\Desktop\korea cnada study\merge korea canada\final.sav
	Active Dataset	DataSet1
	Filter	<none>
	Weight	<none>
	Split File	Nation
	N of Rows in Working Data File	1837
Missing Value Handling	Definition of Missing	User-defined missing values are treated as missing.
	Cases Used	Statistics are based on cases with no missing values for any variable used.
Syntax		REGRESSION /MISSING LISTWISE /STATISTICS COEFF OUTS CI(95) R ANOVA COLLIN TOL CHANGE ZPP /CRITERIA=PIN(.05) POUT(.10) /NOORIGIN /DEPENDENT psychological_well_being /METHOD=ENTER age sex extraversion agreeable conscientiousness neuroticism openness /METHOD=ENTER eudaimonism inclusive externality fear transformative fragility valuing inflexibility.
Resources	Processor Time	00:00:00.08
	Elapsed Time	00:00:00.05

Notes

	Memory Required	21520 bytes
	Additional Memory Required for Residual Plots	0 bytes

Variables Entered/Removed[a]

Nation	Model	Variables Entered	Variables Removed	Method
Korea	1	openness, age, sex, extraversion, neuroticism, conscientiousness, agreeable[b]	.	Enter
	2	inflexibility, transformative, inclusive, eudaimonism, fragility, valuing, fear, externality[b]	.	Enter
Canada	1	openness, sex, conscientiousness, extraversion, age, agreeable, neuroticism[b]	.	Enter
	2	transformative, inclusive, eudaimonism, inflexibility, fragility, valuing, fear, externality[b]	.	Enter

a. Dependent Variable: psychological_well_being

b. All requested variables entered.

Model Summary

Nation	Model	R	R Square	Adjusted R Square	Std. Error of the Estimate	Change ... R Square Change
Korea	1	.535[a]	.286	.282	.87991	.286
	2	.609[b]	.371	.363	.82853	.085
Canada	1	.600[c]	.360	.354	1.01684	.360
	2	.629[d]	.396	.382	.99446	.035

Model Summary

Nation	Model	Change Statistics F Change	df1	df2	Sig. F Change
Korea	1	66.880	7	1169	.000
	2	19.686	8	1161	.000
Canada	1	52.506	7	652	.000
	2	4.709	8	644	.000

a. Predictors: (Constant), openness, age, sex, extraversion, neuroticism, conscientiousness, agreeable

b. Predictors: (Constant), openness, age, sex, extraversion, neuroticism, conscientiousness, agreeable, inflexibility, transformative, inclusive, eudaimonism, fragility, valuing, fear, externality

c. Predictors: (Constant), openness, sex, conscientiousness, extraversion, age, agreeable, neuroticism

d. Predictors: (Constant), openness, sex, conscientiousness, extraversion, age, agreeable, neuroticism, transformative, inclusive, eudaimonism, inflexibility, fragility, valuing, fear, externality

ANOVA[a]

Nation	Model		Sum of Squares	df	Mean Square	F	Sig.
Korea	1	Regression	362.469	7	51.781	66.880	.000[b]
		Residual	905.089	1169	.774		
		Total	1267.558	1176			
	2	Regression	470.576	15	31.372	45.701	.000[c]
		Residual	796.982	1161	.686		
		Total	1267.558	1176			
Canada	1	Regression	380.026	7	54.289	52.506	.000[d]
		Residual	674.143	652	1.034		
		Total	1054.169	659			
	2	Regression	417.285	15	27.819	28.130	.000[e]
		Residual	636.884	644	.989		
		Total	1054.169	659			

a. Dependent Variable: psychological_well_being

b. Predictors: (Constant), openness, age, sex, extraversion, neuroticism, conscientiousness, agreeable

c. Predictors: (Constant), openness, age, sex, extraversion, neuroticism, conscientiousness, agreeable, inflexibility, transformative, inclusive, eudaimonism, fragility, valuing, fear, externality

d. Predictors: (Constant), openness, sex, conscientiousness, extraversion, age, agreeable, neuroticism

e. Predictors: (Constant), openness, sex, conscientiousness, extraversion, age, agreeable, neuroticism, transformative, inclusive, eudaimonism, inflexibility, fragility, valuing, fear, externality

Coefficients[a]

Nation	Model		Unstandardized Coefficients B	Unstandardized Coefficients Std. Error	Standardized Coefficients Beta	t
Korea	1	(Constant)	-.238	.272		-.875
		age	.003	.002	.029	1.132
		sex	.079	.053	.038	1.489
		extraversion	.378	.042	.260	9.050
		agreeable	.155	.047	.095	3.270
		conscientiousness	.221	.039	.148	5.610
		neuroticism	-.381	.040	-.250	-9.621
		openness	.260	.041	.165	6.382
	2	(Constant)	-.238	.320		-.744
		age	-.002	.002	-.021	-.828
		sex	.070	.051	.034	1.372
		extraversion	.339	.040	.233	8.565
		agreeable	.086	.045	.052	1.890
		conscientiousness	.196	.037	.132	5.247
		neuroticism	-.337	.040	-.221	-8.439
		openness	.251	.039	.160	6.485
		eudaimonism	.007	.005	.035	1.447
		inclusive	.121	.022	.132	5.402
		externality	-.092	.028	-.094	-3.271
		fear	-.113	.028	-.117	-4.075
		transformative	.125	.025	.137	5.067
		fragility	-.132	.025	-.135	-5.335
		valuing	.173	.038	.119	4.574
		inflexibility	-.001	.024	-.001	-.036
Canada	1	(Constant)	1.056	.403		2.618
		age	.002	.003	.023	.682
		sex	-.026	.088	-.010	-.291
		extraversion	.283	.043	.219	6.514
		agreeable	.306	.055	.197	5.519
		conscientiousness	.144	.051	.097	2.848
		neuroticism	-.459	.049	-.334	-9.307
		openness	.113	.053	.069	2.120
	2	(Constant)	1.228	.482		2.548
		age	.003	.003	.038	1.129
		sex	-.100	.087	-.038	-1.149
		extraversion	.224	.044	.173	5.069
		agreeable	.237	.057	.152	4.170

Coefficients[a]

Nation	Model		Sig.	95.0% Confidence Interval for B Lower Bound	Upper Bound	Correlations Zero-order
Korea	1	(Constant)	.382	-.772	.296	
		age	.258	-.002	.007	.077
		sex	.137	-.025	.183	-.006
		extraversion	.000	.296	.459	.366
		agreeable	.001	.062	.248	.286
		conscientiousness	.000	.144	.298	.280
		neuroticism	.000	-.459	-.303	-.296
		openness	.000	.180	.340	.278
	2	(Constant)	.457	-.865	.389	
		age	.408	-.006	.002	.077
		sex	.170	-.030	.169	-.006
		extraversion	.000	.261	.416	.366
		agreeable	.059	-.003	.175	.286
		conscientiousness	.000	.123	.270	.280
		neuroticism	.000	-.415	-.258	-.296
		openness	.000	.175	.327	.278
		eudaimonism	.148	-.002	.016	.125
		inclusive	.000	.077	.165	.244
		externality	.001	-.147	-.037	-.265
		fear	.000	-.167	-.059	-.211
		transformative	.000	.076	.173	.110
		fragility	.000	-.181	-.084	-.174
		valuing	.000	.099	.247	.009
		inflexibility	.971	-.049	.047	-.088
Canada	1	(Constant)	.009	.264	1.848	
		age	.496	-.003	.007	.190
		sex	.771	-.197	.146	-.007
		extraversion	.000	.198	.369	.390
		agreeable	.000	.197	.415	.358
		conscientiousness	.005	.045	.244	.301
		neuroticism	.000	-.555	-.362	-.473
		openness	.034	.008	.217	.186
	2	(Constant)	.011	.282	2.174	
		age	.259	-.002	.008	.190
		sex	.251	-.270	.071	-.007
		extraversion	.000	.137	.311	.390
		agreeable	.000	.125	.349	.358

Coefficients[a]

Nation	Model		Correlations Partial	Correlations Part	Collinearity Statistics Tolerance	Collinearity Statistics VIF
Korea	1	(Constant)				
		age	.033	.028	.920	1.086
		sex	.043	.037	.941	1.063
		extraversion	.256	.224	.739	1.354
		agreeable	.095	.081	.730	1.370
		conscientiousness	.162	.139	.876	1.142
		neuroticism	-.271	-.238	.903	1.108
		openness	.183	.158	.908	1.101
	2	(Constant)				
		age	-.024	-.019	.871	1.148
		sex	.040	.032	.908	1.101
		extraversion	.244	.199	.729	1.372
		agreeable	.055	.044	.702	1.424
		conscientiousness	.152	.122	.861	1.161
		neuroticism	-.240	-.196	.789	1.267
		openness	.187	.151	.894	1.118
		eudaimonism	.042	.034	.917	1.090
		inclusive	.157	.126	.911	1.097
		externality	-.096	-.076	.655	1.526
		fear	-.119	-.095	.656	1.526
		transformative	.147	.118	.742	1.347
		fragility	-.155	-.124	.845	1.183
		valuing	.133	.106	.803	1.246
		inflexibility	-.001	-.001	.853	1.172
Canada	1	(Constant)				
		age	.027	.021	.862	1.159
		sex	-.011	-.009	.876	1.142
		extraversion	.247	.204	.864	1.157
		agreeable	.211	.173	.772	1.296
		conscientiousness	.111	.089	.848	1.180
		neuroticism	-.342	-.291	.764	1.310
		openness	.083	.066	.922	1.085
	2	(Constant)				
		age	.044	.035	.829	1.206
		sex	-.045	-.035	.852	1.174
		extraversion	.196	.155	.801	1.249
		agreeable	.162	.128	.701	1.426

Coefficients[a]

Nation	Model		Unstandardized Coefficients B	Unstandardized Coefficients Std. Error	Standardized Coefficients Beta	t
		conscientiousness	.138	.050	.093	2.750
		neuroticism	-.401	.053	-.292	-7.605
		openness	.068	.053	.042	1.269
		eudaimonism	.010	.007	.041	1.275
		inclusive	.113	.032	.117	3.546
		externality	-.060	.040	-.063	-1.490
		fear	-.110	.038	-.121	-2.881
		transformative	.045	.034	.046	1.339
		fragility	-.030	.035	-.031	-.880
		valuing	.046	.048	.036	.968
		inflexibility	.008	.037	.008	.228

Coefficients[a]

Nation	Model		Sig.	95.0% Confidence Interval for B Lower Bound	95.0% Confidence Interval for B Upper Bound	Correlations Zero-order
		conscientiousness	.006	.039	.236	.301
		neuroticism	.000	-.505	-.298	-.473
		openness	.205	-.037	.173	.186
		eudaimonism	.203	-.005	.024	.138
		inclusive	.000	.050	.175	.197
		externality	.137	-.139	.019	-.311
		fear	.004	-.185	-.035	-.363
		transformative	.181	-.021	.112	.007
		fragility	.379	-.098	.037	-.237
		valuing	.334	-.048	.140	-.192
		inflexibility	.819	-.063	.080	-.096

Coefficients[a]

Nation	Model		Correlations Partial	Correlations Part	Collinearity Statistics Tolerance	Collinearity Statistics VIF
		conscientiousness	.108	.084	.826	1.211
		neuroticism	-.287	-.233	.637	1.569
		openness	.050	.039	.876	1.142
		eudaimonism	.050	.039	.904	1.106
		inclusive	.138	.109	.856	1.168
		externality	-.059	-.046	.529	1.889
		fear	-.113	-.088	.530	1.888
		transformative	.053	.041	.807	1.238
		fragility	-.035	-.027	.746	1.341
		valuing	.038	.030	.676	1.480
		inflexibility	.009	.007	.713	1.402

a. Dependent Variable: psychological_well_being

Excluded Variables[a]

Nation	Model		Beta In	t	Sig.	Partial Correlation	Collinearity . Tolerance
Korea	1	eudaimonism	.058[b]	2.308	.021	.067	.956
		inclusive	.176[b]	7.122	.000	.204	.961
		externality	-.140[b]	-5.483	.000	-.158	.917
		fear	-.102[b]	-4.038	.000	-.117	.937
		transformative	.103[b]	4.148	.000	.120	.972
		fragility	-.119[b]	-4.787	.000	-.139	.966
		valuing	.078[b]	3.032	.002	.088	.906
		inflexibility	-.040[b]	-1.605	.109	-.047	.973
Canada	1	eudaimonism	.053[c]	1.640	.102	.064	.925
		inclusive	.122[c]	3.798	.000	.147	.936
		externality	-.097[c]	-2.852	.004	-.111	.832
		fear	-.126[c]	-3.600	.000	-.140	.790
		transformative	.024[c]	.744	.457	.029	.948
		fragility	-.062[c]	-1.860	.063	-.073	.889
		valuing	.011[c]	.314	.754	.012	.842
		inflexibility	-.032[c]	-.994	.321	-.039	.918

Excluded Variables[a]

Nation	Model		Collinearity Statistics VIF	Minimum Tolerance
Korea	1	eudaimonism	1.045	.729
		inclusive	1.040	.725
		externality	1.090	.726
		fear	1.067	.725
		transformative	1.029	.723
		fragility	1.035	.730
		valuing	1.104	.730
		inflexibility	1.028	.721
Canada	1	eudaimonism	1.081	.754
		inclusive	1.068	.764
		externality	1.202	.735
		fear	1.266	.720
		transformative	1.055	.759
		fragility	1.125	.722
		valuing	1.188	.693
		inflexibility	1.089	.744

a. Dependent Variable: psychological_well_being

b. Predictors in the Model: (Constant), openness, age, sex, extraversion, neuroticism, conscientiousness, agreeable

c. Predictors in the Model: (Constant), openness, sex, conscientiousness, extraversion, age, agreeable,

Collinearity Diagnostics[a]

Nation	Model	Dimension	Eigenvalue	Condition Index	Variance Proportions (Constant)	age	sex
Korea	1	1	7.661	1.000	.00	.00	.00
		2	.105	8.547	.00	.22	.46
		3	.080	9.769	.00	.39	.22
		4	.057	11.572	.00	.00	.24
		5	.040	13.817	.00	.18	.02
		6	.027	16.936	.00	.08	.01
		7	.022	18.558	.00	.00	.02
		8	.007	32.008	.99	.12	.03
	2	1	15.013	1.000	.00	.00	.00
		2	.194	8.799	.00	.01	.01
		3	.132	10.681	.00	.03	.20
		4	.109	11.763	.00	.02	.02
		5	.095	12.593	.00	.12	.17
		6	.087	13.142	.00	.05	.23
		7	.075	14.174	.00	.33	.11
		8	.057	16.243	.00	.15	.02
		9	.054	16.706	.00	.06	.11
		10	.044	18.477	.00	.00	.00
		11	.041	19.092	.00	.05	.05
		12	.030	22.306	.00	.03	.00
		13	.026	23.802	.00	.09	.00
		14	.022	26.408	.00	.00	.01
		15	.017	29.438	.00	.02	.03
		16	.005	54.024	.99	.04	.04
Canada	1	1	7.555	1.000	.00	.00	.00
		2	.149	7.123	.00	.05	.04
		3	.095	8.928	.00	.34	.06
		4	.070	10.412	.00	.01	.51
		5	.055	11.677	.00	.34	.16
		6	.040	13.690	.00	.04	.01
		7	.028	16.433	.00	.03	.16
		8	.008	30.436	1.00	.20	.05
	2	1	14.609	1.000	.00	.00	.00
		2	.396	6.072	.00	.00	.00
		3	.173	9.178	.00	.04	.02
		4	.131	10.545	.00	.07	.00
		5	.107	11.702	.00	.00	.11

Collinearity Diagnostics[a]

Nation	Model	Dimension	Variance Proportions extraversion	agreeable	conscientiousness	neuroticism
Korea	1	1	.00	.00	.00	.00
		2	.00	.00	.02	.04
		3	.13	.03	.00	.00
		4	.09	.02	.00	.56
		5	.32	.01	.20	.08
		6	.00	.00	.56	.02
		7	.46	.83	.05	.01
		8	.00	.11	.17	.30
	2	1	.00	.00	.00	.00
		2	.01	.00	.00	.00
		3	.01	.01	.00	.02
		4	.00	.00	.01	.00
		5	.00	.00	.01	.01
		6	.06	.02	.00	.00
		7	.04	.00	.00	.00
		8	.12	.01	.00	.02
		9	.05	.00	.00	.17
		10	.01	.01	.01	.24
		11	.14	.00	.14	.19
		12	.09	.00	.15	.04
		13	.01	.00	.49	.01
		14	.46	.81	.03	.00
		15	.00	.04	.07	.21
		16	.00	.08	.09	.07
Canada	1	1	.00	.00	.00	.00
		2	.16	.00	.01	.25
		3	.45	.00	.02	.00
		4	.21	.02	.02	.26
		5	.11	.01	.16	.02
		6	.03	.03	.54	.06
		7	.02	.92	.02	.02
		8	.03	.02	.22	.40
	2	1	.00	.00	.00	.00
		2	.03	.00	.00	.00
		3	.02	.00	.00	.05
		4	.01	.00	.02	.00
		5	.02	.00	.00	.10

Collinearity Diagnostics[a]

Nation	Model	Dimension	Variance Proportions openness	eudaimonism	inclusive	externality	fear
Korea	1	1	.00				
		2	.00				
		3	.02				
		4	.00				
		5	.22				
		6	.58				
		7	.03				
		8	.14				
	2	1	.00	.00	.00	.00	.00
		2	.00	.04	.07	.10	.14
		3	.00	.14	.08	.01	.09
		4	.00	.21	.68	.00	.01
		5	.00	.12	.00	.02	.07
		6	.01	.22	.01	.07	.03
		7	.01	.20	.06	.10	.00
		8	.00	.01	.00	.03	.23
		9	.00	.00	.02	.10	.11
		10	.02	.00	.00	.49	.22
		11	.14	.01	.00	.00	.07
		12	.11	.01	.02	.02	.00
		13	.58	.00	.00	.03	.01
		14	.03	.00	.01	.01	.01
		15	.00	.00	.02	.01	.01
		16	.08	.03	.01	.00	.00
Canada	1	1	.00				
		2	.00				
		3	.00				
		4	.00				
		5	.23				
		6	.40				
		7	.18				
		8	.17				
	2	1	.00	.00	.00	.00	.00
		2	.00	.01	.01	.04	.12
		3	.00	.01	.01	.01	.01
		4	.00	.01	.65	.00	.00
		5	.00	.15	.00	.04	.24

Collinearity Diagnostics[a]

Nation	Model	Dimension	Variance Proportions transformative	fragility	valuing	inflexibility
Korea	1	1				
		2				
		3				
		4				
		5				
		6				
		7				
		8				
	2	1	.00	.00	.00	.00
		2	.00	.00	.00	.01
		3	.03	.00	.00	.00
		4	.00	.00	.00	.00
		5	.05	.00	.00	.16
		6	.06	.01	.00	.03
		7	.10	.01	.00	.00
		8	.12	.08	.00	.22
		9	.02	.04	.01	.39
		10	.25	.01	.00	.05
		11	.12	.10	.00	.03
		12	.19	.63	.00	.01
		13	.03	.00	.02	.00
		14	.02	.02	.01	.00
		15	.00	.03	.84	.02
		16	.00	.05	.11	.07
Canada	1	1				
		2				
		3				
		4				
		5				
		6				
		7				
		8				
	2	1	.00	.00	.00	.00
		2	.00	.00	.00	.02
		3	.03	.01	.00	.31
		4	.00	.01	.00	.00
		5	.10	.00	.01	.01

Collinearity Diagnostics[a]

Nation	Model	Dimension	Eigenvalue	Condition Index	Variance Proportions (Constant)	age	sex
		6	.097	12.289	.00	.00	.00
		7	.086	13.039	.00	.21	.09
		8	.073	14.174	.00	.01	.07
		9	.070	14.491	.00	.10	.10
		10	.061	15.481	.00	.00	.33
		11	.051	16.871	.00	.35	.02
		12	.044	18.168	.00	.00	.03
		13	.038	19.549	.00	.00	.01
		14	.031	21.685	.00	.01	.03
		15	.026	23.599	.00	.05	.12
		16	.006	50.079	1.00	.15	.06

Collinearity Diagnostics[a]

Nation	Model	Dimension	Variance Proportions extraversion	agreeable	conscientiousness	neuroticism
		6	.38	.00	.00	.00
		7	.13	.00	.01	.01
		8	.17	.00	.00	.06
		9	.06	.00	.00	.01
		10	.01	.01	.00	.19
		11	.04	.00	.35	.05
		12	.05	.01	.00	.08
		13	.00	.01	.28	.03
		14	.03	.00	.10	.27
		15	.03	.94	.04	.00
		16	.03	.02	.20	.14

Collinearity Diagnostics[a]

Nation	Model	Dimension	Variance Proportions openness	eudaimonism	inclusive	externality	fear
		6	.00	.40	.01	.01	.08
		7	.00	.02	.20	.06	.00
		8	.01	.17	.00	.06	.02
		9	.00	.00	.03	.33	.44
		10	.01	.00	.00	.36	.05
		11	.08	.05	.00	.00	.02
		12	.22	.12	.02	.03	.00
		13	.39	.03	.00	.00	.00
		14	.07	.00	.07	.05	.00
		15	.10	.01	.00	.01	.02
		16	.12	.03	.00	.00	.00

Collinearity Diagnostics[a]

Nation	Model	Dimension	Variance Proportions transformative	fragility	valuing	inflexibility
		6	.00	.00	.00	.06
		7	.00	.02	.00	.28
		8	.38	.03	.00	.08
		9	.16	.03	.01	.00
		10	.00	.06	.02	.14
		11	.08	.03	.00	.00
		12	.13	.55	.00	.00
		13	.05	.21	.12	.00
		14	.01	.02	.75	.00
		15	.03	.00	.01	.03
		16	.02	.02	.06	.06

a. Dependent Variable: psychological_well_being

```
REGRESSION
 /MISSING LISTWISE
 /STATISTICS COEFF OUTS CI(95) R ANOVA COLLIN TOL CHANGE ZPP
 /CRITERIA=PIN(.05) POUT(.10)
 /NOORIGIN
 /DEPENDENT life_satisfaction
 /METHOD=ENTER age sex extraversion agreeable conscientiousness neuroticis
```

```
m openness
  /METHOD=ENTER eudaimonism inclusive externality fear transformative fragi
lity valuing
    inflexibility.
```

Regression

Notes

Output Created		20-MAY-2019 12:23:01
Comments		
Input	Data	C:\Users\1\Desktop\korea cnada study\merge korea canada\final.sav
	Active Dataset	DataSet1
	Filter	<none>
	Weight	<none>
	Split File	Nation
	N of Rows in Working Data File	1837
Missing Value Handling	Definition of Missing	User-defined missing values are treated as missing.
	Cases Used	Statistics are based on cases with no missing values for any variable used.
Syntax		REGRESSION<br>/MISSING LISTWISE<br>/STATISTICS COEFF OUTS CI(95) R ANOVA COLLIN TOL CHANGE ZPP<br>/CRITERIA=PIN(.05) POUT(.10)<br>/NOORIGIN<br>/DEPENDENT life_satisfaction<br>/METHOD=ENTER age sex extraversion agreeable conscientiousness neuroticism openness<br>/METHOD=ENTER eudaimonism inclusive externality fear transformative fragility valuing<br>inflexibility.
Resources	Processor Time	00:00:00.06
	Elapsed Time	00:00:00.05

Notes

	Memory Required	21520 bytes
	Additional Memory Required for Residual Plots	0 bytes

Variables Entered/Removed[a]

Nation	Model	Variables Entered	Variables Removed	Method
Korea	1	openness, age, sex, extraversion, neuroticism, conscientiousness, agreeable[b]	.	Enter
	2	inflexibility, transformative, inclusive, eudaimonism, fragility, valuing, fear, externality[b]	.	Enter
Canada	1	openness, sex, conscientiousness, extraversion, age, agreeable, neuroticism[b]	.	Enter
	2	transformative, inclusive, eudaimonism, inflexibility, fragility, valuing, fear, externality[b]	.	Enter

a. Dependent Variable: life_satisfaction

b. All requested variables entered.

Model Summary

Nation	Model	R	R Square	Adjusted R Square	Std. Error of the Estimate	Change ... R Square Change
Korea	1	.402[a]	.162	.157	1.25414	.162
	2	.518[b]	.268	.259	1.17551	.107
Canada	1	.586[c]	.343	.336	1.26618	.343
	2	.635[d]	.403	.389	1.21503	.059

Model Summary

		Change Statistics			
Nation	Model	F Change	df1	df2	Sig. F Change
Korea	1	32.176	7	1169	.000
	2	21.205	8	1161	.000
Canada	1	48.694	7	652	.000
	2	8.006	8	644	.000

a. Predictors: (Constant), openness, age, sex, extraversion, neuroticism, conscientiousness, agreeable

b. Predictors: (Constant), openness, age, sex, extraversion, neuroticism, conscientiousness, agreeable, inflexibility, transformative, inclusive, eudaimonism, fragility, valuing, fear, externality

c. Predictors: (Constant), openness, sex, conscientiousness, extraversion, age, agreeable, neuroticism

d. Predictors: (Constant), openness, sex, conscientiousness, extraversion, age, agreeable, neuroticism, transformative, inclusive, eudaimonism, inflexibility, fragility, valuing, fear, externality

ANOVA[a]

Nation	Model		Sum of Squares	df	Mean Square	F	Sig.
Korea	1	Regression	354.265	7	50.609	32.176	.000[b]
		Residual	1838.695	1169	1.573		
		Total	2192.960	1176			
	2	Regression	588.675	15	39.245	28.401	.000[c]
		Residual	1604.285	1161	1.382		
		Total	2192.960	1176			
Canada	1	Regression	546.471	7	78.067	48.694	.000[d]
		Residual	1045.295	652	1.603		
		Total	1591.766	659			
	2	Regression	641.025	15	42.735	28.947	.000[e]
		Residual	950.741	644	1.476		
		Total	1591.766	659			

a. Dependent Variable: life_satisfaction

b. Predictors: (Constant), openness, age, sex, extraversion, neuroticism, conscientiousness, agreeable

c. Predictors: (Constant), openness, age, sex, extraversion, neuroticism, conscientiousness, agreeable, inflexibility, transformative, inclusive, eudaimonism, fragility, valuing, fear, externality

d. Predictors: (Constant), openness, sex, conscientiousness, extraversion, age, agreeable, neuroticism

e. Predictors: (Constant), openness, sex, conscientiousness, extraversion, age, agreeable, neuroticism, transformative, inclusive, eudaimonism, inflexibility, fragility, valuing, fear, externality

Coefficients[a]

Nation	Model		Unstandardized Coefficients B	Unstandardized Coefficients Std. Error	Standardized Coefficients Beta	t
Korea	1	(Constant)	2.431	.388		6.270
		age	-.007	.003	-.061	-2.183
		sex	.196	.075	.072	2.598
		extraversion	.378	.059	.198	6.357
		agreeable	.155	.067	.072	2.304
		conscientiousness	.193	.056	.099	3.447
		neuroticism	-.507	.056	-.253	-8.981
		openness	.102	.058	.049	1.757
	2	(Constant)	3.631	.453		8.009
		age	-.011	.003	-.094	-3.489
		sex	.145	.072	.053	2.022
		extraversion	.331	.056	.173	5.897
		agreeable	.066	.064	.031	1.024
		conscientiousness	.191	.053	.097	3.601
		neuroticism	-.348	.057	-.174	-6.145
		openness	.091	.055	.044	1.662
		eudaimonism	-.014	.007	-.054	-2.064
		inclusive	.169	.032	.140	5.305
		externality	-.200	.040	-.156	-5.036
		fear	-.109	.039	-.086	-2.773
		transformative	.185	.035	.154	5.295
		fragility	-.222	.035	-.172	-6.313
		valuing	-.044	.054	-.023	-.822
		inflexibility	.061	.035	.048	1.760
Canada	1	(Constant)	4.243	.502		8.450
		age	.002	.003	.019	.543
		sex	.224	.109	.070	2.053
		extraversion	.215	.054	.135	3.965
		agreeable	.205	.069	.108	2.976
		conscientiousness	.095	.063	.052	1.509
		neuroticism	-.804	.061	-.476	-13.101
		openness	-.047	.066	-.024	-.717
	2	(Constant)	4.796	.589		8.146
		age	.003	.003	.026	.773
		sex	.105	.106	.033	.989
		extraversion	.106	.054	.067	1.971
		agreeable	.157	.069	.082	2.253

Coefficients[a]

Nation	Model		Sig.	95.0% Confidence Interval for B Lower Bound	Upper Bound	Correlations Zero-order
Korea	1	(Constant)	.000	1.670	3.192	
		age	.029	-.013	-.001	-.015
		sex	.009	.048	.344	.037
		extraversion	.000	.261	.495	.267
		agreeable	.021	.023	.288	.214
		conscientiousness	.001	.083	.304	.178
		neuroticism	.000	-.618	-.396	-.258
		openness	.079	-.012	.216	.138
	2	(Constant)	.000	2.742	4.521	
		age	.001	-.017	-.005	-.015
		sex	.043	.004	.287	.037
		extraversion	.000	.221	.441	.267
		agreeable	.306	-.060	.192	.214
		conscientiousness	.000	.087	.295	.178
		neuroticism	.000	-.459	-.237	-.258
		openness	.097	-.016	.199	.138
		eudaimonism	.039	-.027	-.001	.017
		inclusive	.000	.107	.232	.218
		externality	.000	-.278	-.122	-.293
		fear	.006	-.186	-.032	-.214
		transformative	.000	.116	.254	.080
		fragility	.000	-.291	-.153	-.220
		valuing	.411	-.149	.061	-.127
		inflexibility	.079	-.007	.129	-.057
Canada	1	(Constant)	.000	3.257	5.229	
		age	.587	-.005	.009	.192
		sex	.041	.010	.438	.028
		extraversion	.000	.108	.321	.299
		agreeable	.003	.070	.341	.257
		conscientiousness	.132	-.029	.219	.261
		neuroticism	.000	-.924	-.683	-.542
		openness	.474	-.178	.083	.067
	2	(Constant)	.000	3.640	5.953	
		age	.440	-.004	.009	.192
		sex	.323	-.103	.313	.028
		extraversion	.049	.000	.212	.299
		agreeable	.025	.020	.293	.257

Coefficients[a]

Nation	Model		Correlations Partial	Correlations Part	Collinearity Statistics Tolerance	Collinearity Statistics VIF
Korea	1	(Constant)				
		age	-.064	-.058	.920	1.086
		sex	.076	.070	.941	1.063
		extraversion	.183	.170	.739	1.354
		agreeable	.067	.062	.730	1.370
		conscientiousness	.100	.092	.876	1.142
		neuroticism	-.254	-.241	.903	1.108
		openness	.051	.047	.908	1.101
	2	(Constant)				
		age	-.102	-.088	.871	1.148
		sex	.059	.051	.908	1.101
		extraversion	.171	.148	.729	1.372
		agreeable	.030	.026	.702	1.424
		conscientiousness	.105	.090	.861	1.161
		neuroticism	-.177	-.154	.789	1.267
		openness	.049	.042	.894	1.118
		eudaimonism	-.060	-.052	.917	1.090
		inclusive	.154	.133	.911	1.097
		externality	-.146	-.126	.655	1.526
		fear	-.081	-.070	.656	1.526
		transformative	.154	.133	.742	1.347
		fragility	-.182	-.158	.845	1.183
		valuing	-.024	-.021	.803	1.246
		inflexibility	.052	.044	.853	1.172
Canada	1	(Constant)				
		age	.021	.017	.862	1.159
		sex	.080	.065	.876	1.142
		extraversion	.153	.126	.864	1.157
		agreeable	.116	.094	.772	1.296
		conscientiousness	.059	.048	.848	1.180
		neuroticism	-.456	-.416	.764	1.310
		openness	-.028	-.023	.922	1.085
	2	(Constant)				
		age	.030	.024	.829	1.206
		sex	.039	.030	.852	1.174
		extraversion	.077	.060	.801	1.249
		agreeable	.088	.069	.701	1.426

Coefficients[a]

Nation	Model		Unstandardized Coefficients B	Unstandardized Coefficients Std. Error	Standardized Coefficients Beta	t
		conscientiousness	.086	.061	.047	1.397
		neuroticism	-.680	.064	-.403	-10.558
		openness	-.074	.065	-.037	-1.140
		eudaimonism	-.016	.009	-.057	-1.786
		inclusive	.150	.039	.127	3.865
		externality	-.140	.049	-.119	-2.850
		fear	-.172	.047	-.154	-3.684
		transformative	.087	.041	.071	2.093
		fragility	-.074	.042	-.062	-1.745
		valuing	.037	.058	.023	.630
		inflexibility	.115	.045	.093	2.570

Coefficients[a]

Nation	Model		Sig.	95.0% Confidence Interval for B Lower Bound	95.0% Confidence Interval for B Upper Bound	Correlations Zero-order
		conscientiousness	.163	-.035	.206	.261
		neuroticism	.000	-.807	-.554	-.542
		openness	.255	-.202	.054	.067
		eudaimonism	.075	-.034	.002	.023
		inclusive	.000	.074	.226	.178
		externality	.005	-.236	-.044	-.322
		fear	.000	-.264	-.080	-.377
		transformative	.037	.005	.168	-.014
		fragility	.081	-.156	.009	-.278
		valuing	.529	-.078	.152	-.225
		inflexibility	.010	.027	.202	-.011

Coefficients[a]

Nation	Model		Correlations Partial	Correlations Part	Collinearity Statistics Tolerance	Collinearity Statistics VIF
		conscientiousness	.055	.043	.826	1.211
		neuroticism	-.384	-.322	.637	1.569
		openness	-.045	-.035	.876	1.142
		eudaimonism	-.070	-.054	.904	1.106
		inclusive	.151	.118	.856	1.168
		externality	-.112	-.087	.529	1.889
		fear	-.144	-.112	.530	1.888
		transformative	.082	.064	.807	1.238
		fragility	-.069	-.053	.746	1.341
		valuing	.025	.019	.676	1.480
		inflexibility	.101	.078	.713	1.402

a. Dependent Variable: life_satisfaction

Excluded Variables[a]

Nation	Model		Beta In	t	Sig.	Partial Correlation	Collinearity . Tolerance
Korea	1	eudaimonism	-.022[b]	-.802	.423	-.023	.956
		inclusive	.173[b]	6.442	.000	.185	.961
		externality	-.206[b]	-7.539	.000	-.215	.917
		fear	-.128[b]	-4.679	.000	-.136	.937
		transformative	.086[b]	3.164	.002	.092	.972
		fragility	-.176[b]	-6.584	.000	-.189	.966
		valuing	-.063[b]	-2.227	.026	-.065	.906
		inflexibility	-.017[b]	-.611	.542	-.018	.973
Canada	1	eudaimonism	-.040[c]	-1.220	.223	-.048	.925
		inclusive	.133[c]	4.088	.000	.158	.936
		externality	-.135[c]	-3.916	.000	-.152	.832
		fear	-.162[c]	-4.605	.000	-.178	.790
		transformative	.017[c]	.527	.599	.021	.948
		fragility	-.105[c]	-3.128	.002	-.122	.889
		valuing	-.010[c]	-.286	.775	-.011	.842
		inflexibility	.020[c]	.607	.544	.024	.918

Excluded Variables[a]

Nation	Model		Collinearity Statistics VIF	Minimum Tolerance
Korea	1	eudaimonism	1.045	.729
		inclusive	1.040	.725
		externality	1.090	.726
		fear	1.067	.725
		transformative	1.029	.723
		fragility	1.035	.730
		valuing	1.104	.730
		inflexibility	1.028	.721
Canada	1	eudaimonism	1.081	.754
		inclusive	1.068	.764
		externality	1.202	.735
		fear	1.266	.720
		transformative	1.055	.759
		fragility	1.125	.722
		valuing	1.188	.693
		inflexibility	1.089	.744

a. Dependent Variable: life_satisfaction

b. Predictors in the Model: (Constant), openness, age, sex, extraversion, neuroticism, conscientiousness, agreeable

c. Predictors in the Model: (Constant), openness, sex, conscientiousness, extraversion, age, agreeable,

Collinearity Diagnostics[a]

Nation	Model	Dimension	Eigenvalue	Condition Index	Variance Proportions (Constant)	age	sex
Korea	1	1	7.661	1.000	.00	.00	.00
		2	.105	8.547	.00	.22	.46
		3	.080	9.769	.00	.39	.22
		4	.057	11.572	.00	.00	.24
		5	.040	13.817	.00	.18	.02
		6	.027	16.936	.00	.08	.01
		7	.022	18.558	.00	.00	.02
		8	.007	32.008	.99	.12	.03
	2	1	15.013	1.000	.00	.00	.00
		2	.194	8.799	.00	.01	.01
		3	.132	10.681	.00	.03	.20
		4	.109	11.763	.00	.02	.02
		5	.095	12.593	.00	.12	.17
		6	.087	13.142	.00	.05	.23
		7	.075	14.174	.00	.33	.11
		8	.057	16.243	.00	.15	.02
		9	.054	16.706	.00	.06	.11
		10	.044	18.477	.00	.00	.00
		11	.041	19.092	.00	.05	.05
		12	.030	22.306	.00	.03	.00
		13	.026	23.802	.00	.09	.00
		14	.022	26.408	.00	.00	.01
		15	.017	29.438	.00	.02	.03
		16	.005	54.024	.99	.04	.04
Canada	1	1	7.555	1.000	.00	.00	.00
		2	.149	7.123	.00	.05	.04
		3	.095	8.928	.00	.34	.06
		4	.070	10.412	.00	.01	.51
		5	.055	11.677	.00	.34	.16
		6	.040	13.690	.00	.04	.01
		7	.028	16.433	.00	.03	.16
		8	.008	30.436	1.00	.20	.05
	2	1	14.609	1.000	.00	.00	.00
		2	.396	6.072	.00	.00	.00
		3	.173	9.178	.00	.04	.02
		4	.131	10.545	.00	.07	.00
		5	.107	11.702	.00	.00	.11

Collinearity Diagnostics[a]

Nation	Model	Dimension	Variance Proportions extraversion	agreeable	conscientiousness	neuroticism
Korea	1	1	.00	.00	.00	.00
		2	.00	.00	.02	.04
		3	.13	.03	.00	.00
		4	.09	.02	.00	.56
		5	.32	.01	.20	.08
		6	.00	.00	.56	.02
		7	.46	.83	.05	.01
		8	.00	.11	.17	.30
	2	1	.00	.00	.00	.00
		2	.01	.00	.00	.00
		3	.01	.01	.00	.02
		4	.00	.00	.01	.00
		5	.00	.00	.01	.01
		6	.06	.02	.00	.00
		7	.04	.00	.00	.00
		8	.12	.01	.00	.02
		9	.05	.00	.00	.17
		10	.01	.01	.01	.24
		11	.14	.00	.14	.19
		12	.09	.00	.15	.04
		13	.01	.00	.49	.01
		14	.46	.81	.03	.00
		15	.00	.04	.07	.21
		16	.00	.08	.09	.07
Canada	1	1	.00	.00	.00	.00
		2	.16	.00	.01	.25
		3	.45	.00	.02	.00
		4	.21	.02	.02	.26
		5	.11	.01	.16	.02
		6	.03	.03	.54	.06
		7	.02	.92	.02	.02
		8	.03	.02	.22	.40
	2	1	.00	.00	.00	.00
		2	.03	.00	.00	.00
		3	.02	.00	.00	.05
		4	.01	.00	.02	.00
		5	.02	.00	.00	.10

Collinearity Diagnostics[a]

Nation	Model	Dimension	Variance Proportions: openness	eudaimonism	inclusive	externality	fear
Korea	1	1	.00				
		2	.00				
		3	.02				
		4	.00				
		5	.22				
		6	.58				
		7	.03				
		8	.14				
	2	1	.00	.00	.00	.00	.00
		2	.00	.04	.07	.10	.14
		3	.00	.14	.08	.01	.09
		4	.00	.21	.68	.00	.01
		5	.00	.12	.00	.02	.07
		6	.01	.22	.01	.07	.03
		7	.01	.20	.06	.10	.00
		8	.00	.01	.00	.03	.23
		9	.00	.00	.02	.10	.11
		10	.02	.00	.00	.49	.22
		11	.14	.01	.00	.00	.07
		12	.11	.01	.02	.02	.00
		13	.58	.00	.00	.03	.01
		14	.03	.00	.01	.01	.01
		15	.00	.00	.02	.01	.01
		16	.08	.03	.01	.00	.00
Canada	1	1	.00				
		2	.00				
		3	.00				
		4	.00				
		5	.23				
		6	.40				
		7	.18				
		8	.17				
	2	1	.00	.00	.00	.00	.00
		2	.00	.01	.01	.04	.12
		3	.00	.01	.01	.01	.01
		4	.00	.01	.65	.00	.00
		5	.00	.15	.00	.04	.24

Collinearity Diagnostics[a]

Nation	Model	Dimension	Variance Proportions: transformative	fragility	valuing	inflexibility
Korea	1	1				
		2				
		3				
		4				
		5				
		6				
		7				
		8				
	2	1	.00	.00	.00	.00
		2	.00	.00	.00	.01
		3	.03	.00	.00	.00
		4	.00	.00	.00	.00
		5	.05	.00	.00	.16
		6	.06	.01	.00	.03
		7	.10	.01	.00	.00
		8	.12	.08	.00	.22
		9	.02	.04	.01	.39
		10	.25	.01	.00	.05
		11	.12	.10	.00	.03
		12	.19	.63	.00	.01
		13	.03	.00	.02	.00
		14	.02	.02	.01	.00
		15	.00	.03	.84	.02
		16	.00	.05	.11	.07
Canada	1	1				
		2				
		3				
		4				
		5				
		6				
		7				
		8				
	2	1	.00	.00	.00	.00
		2	.00	.00	.00	.02
		3	.03	.01	.00	.31
		4	.00	.01	.00	.00
		5	.10	.00	.01	.01

Collinearity Diagnostics[a]

Nation	Model	Dimension	Eigenvalue	Condition Index	Variance Proportions (Constant)	age	sex
		6	.097	12.289	.00	.00	.00
		7	.086	13.039	.00	.21	.09
		8	.073	14.174	.00	.01	.07
		9	.070	14.491	.00	.10	.10
		10	.061	15.481	.00	.00	.33
		11	.051	16.871	.00	.35	.02
		12	.044	18.168	.00	.00	.03
		13	.038	19.549	.00	.00	.01
		14	.031	21.685	.00	.01	.03
		15	.026	23.599	.00	.05	.12
		16	.006	50.079	1.00	.15	.06

Collinearity Diagnostics[a]

Nation	Model	Dimension	Variance Proportions extraversion	agreeable	conscientiousn ess	neuroticism
		6	.38	.00	.00	.00
		7	.13	.00	.01	.01
		8	.17	.00	.00	.06
		9	.06	.00	.00	.01
		10	.01	.01	.00	.19
		11	.04	.00	.35	.05
		12	.05	.01	.00	.08
		13	.00	.01	.28	.03
		14	.03	.00	.10	.27
		15	.03	.94	.04	.00
		16	.03	.02	.20	.14

Collinearity Diagnostics[a]

Nation	Model	Dimension	Variance Proportions openness	eudaimonism	inclusive	externality	fear
		6	.00	.40	.01	.01	.08
		7	.00	.02	.20	.06	.00
		8	.01	.17	.00	.06	.02
		9	.00	.00	.03	.33	.44
		10	.01	.00	.00	.36	.05
		11	.08	.05	.00	.00	.02
		12	.22	.12	.02	.03	.00
		13	.39	.03	.00	.00	.00
		14	.07	.00	.07	.05	.00
		15	.10	.01	.00	.01	.02
		16	.12	.03	.00	.00	.00

Collinearity Diagnostics[a]

Nation	Model	Dimension	Variance Proportions transformative	fragility	valuing	inflexibility
		6	.00	.00	.00	.06
		7	.00	.02	.00	.28
		8	.38	.03	.00	.08
		9	.16	.03	.01	.00
		10	.00	.06	.02	.14
		11	.08	.03	.00	.00
		12	.13	.55	.00	.00
		13	.05	.21	.12	.00
		14	.01	.02	.75	.00
		15	.03	.00	.01	.03
		16	.02	.02	.06	.06

a. Dependent Variable: life_satisfaction

```
REGRESSION
 /MISSING LISTWISE
 /STATISTICS COEFF OUTS CI(95) R ANOVA COLLIN TOL CHANGE ZPP
 /CRITERIA=PIN(.05) POUT(.10)
 /NOORIGIN
 /DEPENDENT negative_affect
 /METHOD=ENTER age sex extraversion agreeable conscientiousness neuroticis
```

```
m openness
  /METHOD=ENTER eudaimonism inclusive externality fear transformative fragi
lity valuing
    inflexibility.
```

Regression

Notes

Output Created		20-MAY-2019 12:23:08
Comments		
Input	Data	C:\Users\1\Desktop\korea cnada study\merge korea canada\final.sav
	Active Dataset	DataSet1
	Filter	<none>
	Weight	<none>
	Split File	Nation
	N of Rows in Working Data File	1837
Missing Value Handling	Definition of Missing	User-defined missing values are treated as missing.
	Cases Used	Statistics are based on cases with no missing values for any variable used.
Syntax		REGRESSION /MISSING LISTWISE /STATISTICS COEFF OUTS CI(95) R ANOVA COLLIN TOL CHANGE ZPP /CRITERIA=PIN(.05) POUT(.10) /NOORIGIN /DEPENDENT negative_affect /METHOD=ENTER age sex extraversion agreeable conscientiousness neuroticism openness /METHOD=ENTER eudaimonism inclusive externality fear transformative fragility valuing inflexibility.
Resources	Processor Time	00:00:00.06
	Elapsed Time	00:00:00.04

Notes

	Memory Required	21520 bytes
	Additional Memory Required for Residual Plots	0 bytes

Variables Entered/Removed[a]

Nation	Model	Variables Entered	Variables Removed	Method
Korea	1	openness, age, sex, extraversion, neuroticism, conscientiousness, agreeable[b]	.	Enter
	2	inflexibility, transformative, inclusive, eudaimonism, fragility, valuing, fear, externality[b]	.	Enter
Canada	1	openness, sex, conscientiousness, extraversion, age, agreeable, neuroticism[b]	.	Enter
	2	transformative, inclusive, eudaimonism, inflexibility, fragility, valuing, fear, externality[b]	.	Enter

a. Dependent Variable: negative_affect

b. All requested variables entered.

Model Summary

Nation	Model	R	R Square	Adjusted R Square	Std. Error of the Estimate	Change ... R Square Change
Korea	1	.501[a]	.251	.247	.67731	.251
	2	.601[b]	.362	.353	.62759	.110
Canada	1	.737[c]	.543	.538	.59867	.543
	2	.770[d]	.594	.584	.56835	.050

Model Summary

		Change Statistics			
Nation	Model	F Change	df1	df2	Sig. F Change
Korea	1	56.101	7	1169	.000
	2	25.071	8	1161	.000
Canada	1	110.835	7	652	.000
	2	9.929	8	644	.000

a. Predictors: (Constant), openness, age, sex, extraversion, neuroticism, conscientiousness, agreeable

b. Predictors: (Constant), openness, age, sex, extraversion, neuroticism, conscientiousness, agreeable, inflexibility, transformative, inclusive, eudaimonism, fragility, valuing, fear, externality

c. Predictors: (Constant), openness, sex, conscientiousness, extraversion, age, agreeable, neuroticism

d. Predictors: (Constant), openness, sex, conscientiousness, extraversion, age, agreeable, neuroticism, transformative, inclusive, eudaimonism, inflexibility, fragility, valuing, fear, externality

ANOVA[a]

Nation	Model		Sum of Squares	df	Mean Square	F	Sig.
Korea	1	Regression	180.153	7	25.736	56.101	.000[b]
		Residual	536.279	1169	.459		
		Total	716.432	1176			
	2	Regression	259.150	15	17.277	43.864	.000[c]
		Residual	457.282	1161	.394		
		Total	716.432	1176			
Canada	1	Regression	278.068	7	39.724	110.835	.000[d]
		Residual	233.682	652	.358		
		Total	511.750	659			
	2	Regression	303.727	15	20.248	62.685	.000[e]
		Residual	208.023	644	.323		
		Total	511.750	659			

a. Dependent Variable: negative_affect

b. Predictors: (Constant), openness, age, sex, extraversion, neuroticism, conscientiousness, agreeable

c. Predictors: (Constant), openness, age, sex, extraversion, neuroticism, conscientiousness, agreeable, inflexibility, transformative, inclusive, eudaimonism, fragility, valuing, fear, externality

d. Predictors: (Constant), openness, sex, conscientiousness, extraversion, age, agreeable, neuroticism

e. Predictors: (Constant), openness, sex, conscientiousness, extraversion, age, agreeable, neuroticism, transformative, inclusive, eudaimonism, inflexibility, fragility, valuing, fear, externality

Coefficients[a]

Nation	Model		Unstandardized Coefficients B	Unstandardized Coefficients Std. Error	Standardized Coefficients Beta	t
Korea	1	(Constant)	1.623	.209		7.749
		age	-.004	.002	-.065	-2.452
		sex	-.101	.041	-.065	-2.491
		extraversion	-.115	.032	-.105	-3.566
		agreeable	-.004	.036	-.003	-.106
		conscientiousness	-.080	.030	-.072	-2.650
		neuroticism	.521	.030	.455	17.086
		openness	-.020	.031	-.017	-.638
	2	(Constant)	.101	.242		.417
		age	-.005	.002	-.083	-3.302
		sex	-.053	.038	-.034	-1.370
		extraversion	-.098	.030	-.090	-3.283
		agreeable	.027	.034	.022	.782
		conscientiousness	-.082	.028	-.073	-2.880
		neuroticism	.395	.030	.345	13.075
		openness	-.004	.029	-.003	-.126
		eudaimonism	.010	.004	.070	2.876
		inclusive	-.007	.017	-.010	-.392
		externality	.121	.021	.165	5.711
		fear	.098	.021	.135	4.675
		transformative	-.023	.019	-.034	-1.243
		fragility	.066	.019	.089	3.502
		valuing	.154	.029	.141	5.400
		inflexibility	-.019	.019	-.026	-1.032
Canada	1	(Constant)	1.585	.237		6.678
		age	-.009	.002	-.155	-5.433
		sex	.123	.052	.068	2.392
		extraversion	-.076	.026	-.085	-2.976
		agreeable	-.058	.033	-.053	-1.768
		conscientiousness	-.147	.030	-.141	-4.916
		neuroticism	.535	.029	.559	18.453
		openness	.063	.031	.055	2.007
	2	(Constant)	.588	.275		2.134
		age	-.008	.002	-.147	-5.336
		sex	.175	.050	.096	3.523
		extraversion	-.053	.025	-.059	-2.099
		agreeable	-.035	.033	-.032	-1.062

Coefficients[a]

Nation	Model		Sig.	95.0% Confidence Interval for B Lower Bound	95.0% Confidence Interval for B Upper Bound	Correlations Zero-order
Korea	1	(Constant)	.000	1.212	2.034	
		age	.014	-.008	-.001	-.151
		sex	.013	-.181	-.022	.027
		extraversion	.000	-.178	-.052	-.127
		agreeable	.916	-.075	.068	-.087
		conscientiousness	.008	-.140	-.021	-.195
		neuroticism	.000	.461	.581	.472
		openness	.524	-.082	.042	-.071
	2	(Constant)	.676	-.374	.576	
		age	.001	-.009	-.002	-.151
		sex	.171	-.128	.023	.027
		extraversion	.001	-.157	-.040	-.127
		agreeable	.434	-.041	.094	-.087
		conscientiousness	.004	-.137	-.026	-.195
		neuroticism	.000	.336	.454	.472
		openness	.900	-.061	.054	-.071
		eudaimonism	.004	.003	.018	-.014
		inclusive	.695	-.040	.027	-.073
		externality	.000	.080	.163	.366
		fear	.000	.057	.139	.324
		transformative	.214	-.060	.013	.074
		fragility	.000	.029	.103	.225
		valuing	.000	.098	.211	.307
		inflexibility	.302	-.056	.017	.071
Canada	1	(Constant)	.000	1.119	2.052	
		age	.000	-.012	-.006	-.381
		sex	.017	.022	.224	.162
		extraversion	.003	-.126	-.026	-.270
		agreeable	.078	-.122	.006	-.201
		conscientiousness	.000	-.205	-.088	-.384
		neuroticism	.000	.478	.592	.692
		openness	.045	.001	.124	-.023
	2	(Constant)	.033	.047	1.129	
		age	.000	-.011	-.005	-.381
		sex	.000	.077	.272	.162
		extraversion	.036	-.103	-.003	-.270
		agreeable	.288	-.098	.029	-.201

Coefficients[a]

Nation	Model		Correlations Partial	Part	Collinearity Statistics Tolerance	VIF
Korea	1	(Constant)				
		age	-.072	-.062	.920	1.086
		sex	-.073	-.063	.941	1.063
		extraversion	-.104	-.090	.739	1.354
		agreeable	-.003	-.003	.730	1.370
		conscientiousness	-.077	-.067	.876	1.142
		neuroticism	.447	.432	.903	1.108
		openness	-.019	-.016	.908	1.101
	2	(Constant)				
		age	-.096	-.077	.871	1.148
		sex	-.040	-.032	.908	1.101
		extraversion	-.096	-.077	.729	1.372
		agreeable	.023	.018	.702	1.424
		conscientiousness	-.084	-.068	.861	1.161
		neuroticism	.358	.307	.789	1.267
		openness	-.004	-.003	.894	1.118
		eudaimonism	.084	.067	.917	1.090
		inclusive	-.011	-.009	.911	1.097
		externality	.165	.134	.655	1.526
		fear	.136	.110	.656	1.526
		transformative	-.036	-.029	.742	1.347
		fragility	.102	.082	.845	1.183
		valuing	.157	.127	.803	1.246
		inflexibility	-.030	-.024	.853	1.172
Canada	1	(Constant)				
		age	-.208	-.144	.862	1.159
		sex	.093	.063	.876	1.142
		extraversion	-.116	-.079	.864	1.157
		agreeable	-.069	-.047	.772	1.296
		conscientiousness	-.189	-.130	.848	1.180
		neuroticism	.586	.488	.764	1.310
		openness	.078	.053	.922	1.085
	2	(Constant)				
		age	-.206	-.134	.829	1.206
		sex	.138	.089	.852	1.174
		extraversion	-.082	-.053	.801	1.249
		agreeable	-.042	-.027	.701	1.426

Coefficients[a]

Nation	Model		Unstandardized Coefficients B	Std. Error	Standardized Coefficients Beta	t
		conscientiousness	-.119	.029	-.114	-4.136
		neuroticism	.446	.030	.466	14.807
		openness	.076	.031	.067	2.477
		eudaimonism	.002	.004	.014	.541
		inclusive	.013	.018	.019	.707
		externality	.076	.023	.114	3.307
		fear	.064	.022	.101	2.939
		transformative	-.003	.019	-.004	-.160
		fragility	.007	.020	.010	.350
		valuing	.106	.027	.118	3.866
		inflexibility	-.027	.021	-.039	-1.303

Coefficients[a]

Nation	Model		Sig.	95.0% Confidence Interval for B Lower Bound	Upper Bound	Correlations Zero-order
		conscientiousness	.000	-.175	-.062	-.384
		neuroticism	.000	.387	.505	.692
		openness	.014	.016	.135	-.023
		eudaimonism	.589	-.006	.011	-.037
		inclusive	.480	-.023	.048	.038
		externality	.001	.031	.121	.365
		fear	.003	.021	.107	.412
		transformative	.873	-.041	.035	.135
		fragility	.726	-.032	.046	.289
		valuing	.000	.052	.160	.426
		inflexibility	.193	-.068	.014	.044

Coefficients[a]

Nation	Model		Correlations Partial	Correlations Part	Collinearity Statistics Tolerance	Collinearity Statistics VIF
		conscientiousness	-.161	-.104	.826	1.211
		neuroticism	.504	.372	.637	1.569
		openness	.097	.062	.876	1.142
		eudaimonism	.021	.014	.904	1.106
		inclusive	.028	.018	.856	1.168
		externality	.129	.083	.529	1.889
		fear	.115	.074	.530	1.888
		transformative	-.006	-.004	.807	1.238
		fragility	.014	.009	.746	1.341
		valuing	.151	.097	.676	1.480
		inflexibility	-.051	-.033	.713	1.402

a. Dependent Variable: negative_affect

Excluded Variables[a]

Nation	Model		Beta In	t	Sig.	Partial Correlation	Collinearity . Tolerance
Korea	1	eudaimonism	.061[b]	2.354	.019	.069	.956
		inclusive	-.006[b]	-.246	.805	-.007	.961
		externality	.255[b]	10.040	.000	.282	.917
		fear	.242[b]	9.633	.000	.271	.937
		transformative	.071[b]	2.757	.006	.080	.972
		fragility	.151[b]	5.953	.000	.172	.966
		valuing	.215[b]	8.336	.000	.237	.906
		inflexibility	.061[b]	2.397	.017	.070	.973
Canada	1	eudaimonism	.005[c]	.190	.850	.007	.925
		inclusive	.056[c]	2.060	.040	.080	.936
		externality	.189[c]	6.720	.000	.255	.832
		fear	.182[c]	6.279	.000	.239	.790
		transformative	.056[c]	2.051	.041	.080	.948
		fragility	.088[c]	3.145	.002	.122	.889
		valuing	.183[c]	6.547	.000	.249	.842
		inflexibility	.062[c]	2.239	.026	.087	.918

Excluded Variables[a]

Nation	Model		Collinearity Statistics VIF	Minimum Tolerance
Korea	1	eudaimonism	1.045	.729
		inclusive	1.040	.725
		externality	1.090	.726
		fear	1.067	.725
		transformative	1.029	.723
		fragility	1.035	.730
		valuing	1.104	.730
		inflexibility	1.028	.721
Canada	1	eudaimonism	1.081	.754
		inclusive	1.068	.764
		externality	1.202	.735
		fear	1.266	.720
		transformative	1.055	.759
		fragility	1.125	.722
		valuing	1.188	.693
		inflexibility	1.089	.744

a. Dependent Variable: negative_affect

[illegible]

b. Predictors in the Model: (Constant), openness, age, sex, extraversion, neuroticism, conscientiousness, agreeable

c. Predictors in the Model: (Constant), openness, sex, conscientiousness, extraversion, age, agreeable,

Collinearity Diagnostics[a]

Nation	Model	Dimension	Eigenvalue	Condition Index	Variance Proportions (Constant)	age	sex
Korea	1	1	7.661	1.000	.00	.00	.00
		2	.105	8.547	.00	.22	.46
		3	.080	9.769	.00	.39	.22
		4	.057	11.572	.00	.00	.24
		5	.040	13.817	.00	.18	.02
		6	.027	16.936	.00	.08	.01
		7	.022	18.558	.00	.00	.02
		8	.007	32.008	.99	.12	.03
	2	1	15.013	1.000	.00	.00	.00
		2	.194	8.799	.00	.01	.01
		3	.132	10.681	.00	.03	.20
		4	.109	11.763	.00	.02	.02
		5	.095	12.593	.00	.12	.17
		6	.087	13.142	.00	.05	.23
		7	.075	14.174	.00	.33	.11
		8	.057	16.243	.00	.15	.02
		9	.054	16.706	.00	.06	.11
		10	.044	18.477	.00	.00	.00
		11	.041	19.092	.00	.05	.05
		12	.030	22.306	.00	.03	.00
		13	.026	23.802	.00	.09	.00
		14	.022	26.408	.00	.00	.01
		15	.017	29.438	.00	.02	.03
		16	.005	54.024	.99	.04	.04
Canada	1	1	7.555	1.000	.00	.00	.00
		2	.149	7.123	.00	.05	.04
		3	.095	8.928	.00	.34	.06
		4	.070	10.412	.00	.01	.51
		5	.055	11.677	.00	.34	.16
		6	.040	13.690	.00	.04	.01
		7	.028	16.433	.00	.03	.16
		8	.008	30.436	1.00	.20	.05
	2	1	14.609	1.000	.00	.00	.00
		2	.396	6.072	.00	.00	.00
		3	.173	9.178	.00	.04	.02
		4	.131	10.545	.00	.07	.00
		5	.107	11.702	.00	.00	.11

Collinearity Diagnostics[a]

Nation	Model	Dimension	Variance Proportions extraversion	agreeable	conscientiousness	neuroticism
Korea	1	1	.00	.00	.00	.00
		2	.00	.00	.02	.04
		3	.13	.03	.00	.00
		4	.09	.02	.00	.56
		5	.32	.01	.20	.08
		6	.00	.00	.56	.02
		7	.46	.83	.05	.01
		8	.00	.11	.17	.30
	2	1	.00	.00	.00	.00
		2	.01	.00	.00	.00
		3	.01	.01	.00	.02
		4	.00	.00	.01	.00
		5	.00	.00	.01	.01
		6	.06	.02	.00	.00
		7	.04	.00	.00	.00
		8	.12	.01	.00	.02
		9	.05	.00	.00	.17
		10	.01	.01	.01	.24
		11	.14	.00	.14	.19
		12	.09	.00	.15	.04
		13	.01	.00	.49	.01
		14	.46	.81	.03	.00
		15	.00	.04	.07	.21
		16	.00	.08	.09	.07
Canada	1	1	.00	.00	.00	.00
		2	.16	.00	.01	.25
		3	.45	.00	.02	.00
		4	.21	.02	.02	.26
		5	.11	.01	.16	.02
		6	.03	.03	.54	.06
		7	.02	.92	.02	.02
		8	.03	.02	.22	.40
	2	1	.00	.00	.00	.00
		2	.03	.00	.00	.00
		3	.02	.00	.00	.05
		4	.01	.00	.02	.00
		5	.02	.00	.00	.10

Collinearity Diagnostics[a]

Nation	Model	Dimension	Variance Proportions openness	eudaimonism	inclusive	externality	fear
Korea	1	1	.00				
		2	.00				
		3	.02				
		4	.00				
		5	.22				
		6	.58				
		7	.03				
		8	.14				
	2	1	.00	.00	.00	.00	.00
		2	.00	.04	.07	.10	.14
		3	.00	.14	.08	.01	.09
		4	.00	.21	.68	.00	.01
		5	.00	.12	.00	.02	.07
		6	.01	.22	.01	.07	.03
		7	.01	.20	.06	.10	.00
		8	.00	.01	.00	.03	.23
		9	.00	.00	.02	.10	.11
		10	.02	.00	.00	.49	.22
		11	.14	.01	.00	.00	.07
		12	.11	.01	.02	.02	.00
		13	.58	.00	.00	.03	.01
		14	.03	.00	.01	.01	.01
		15	.00	.00	.02	.01	.01
		16	.08	.03	.01	.00	.00
Canada	1	1	.00				
		2	.00				
		3	.00				
		4	.00				
		5	.23				
		6	.40				
		7	.18				
		8	.17				
	2	1	.00	.00	.00	.00	.00
		2	.00	.01	.01	.04	.12
		3	.00	.01	.01	.01	.01
		4	.00	.01	.65	.00	.00
		5	.00	.15	.00	.04	.24

Collinearity Diagnostics[a]

Nation	Model	Dimension	Variance Proportions transformative	fragility	valuing	inflexibility
Korea	1	1				
		2				
		3				
		4				
		5				
		6				
		7				
		8				
	2	1	.00	.00	.00	.00
		2	.00	.00	.00	.01
		3	.03	.00	.00	.00
		4	.00	.00	.00	.00
		5	.05	.00	.00	.16
		6	.06	.01	.00	.03
		7	.10	.01	.00	.00
		8	.12	.08	.00	.22
		9	.02	.04	.01	.39
		10	.25	.01	.00	.05
		11	.12	.10	.00	.03
		12	.19	.63	.00	.01
		13	.03	.00	.02	.00
		14	.02	.02	.01	.00
		15	.00	.03	.84	.02
		16	.00	.05	.11	.07
Canada	1	1				
		2				
		3				
		4				
		5				
		6				
		7				
		8				
	2	1	.00	.00	.00	.00
		2	.00	.00	.00	.02
		3	.03	.01	.00	.31
		4	.00	.01	.00	.00
		5	.10	.00	.01	.01

Collinearity Diagnostics[a]

Nation	Model	Dimension	Eigenvalue	Condition Index	Variance Proportions (Constant)	age	sex
		6	.097	12.289	.00	.00	.00
		7	.086	13.039	.00	.21	.09
		8	.073	14.174	.00	.01	.07
		9	.070	14.491	.00	.10	.10
		10	.061	15.481	.00	.00	.33
		11	.051	16.871	.00	.35	.02
		12	.044	18.168	.00	.00	.03
		13	.038	19.549	.00	.00	.01
		14	.031	21.685	.00	.01	.03
		15	.026	23.599	.00	.05	.12
		16	.006	50.079	1.00	.15	.06

Collinearity Diagnostics[a]

Nation	Model	Dimension	Variance Proportions extraversion	agreeable	conscientiousness	neuroticism
		6	.38	.00	.00	.00
		7	.13	.00	.01	.01
		8	.17	.00	.00	.06
		9	.06	.00	.00	.01
		10	.01	.01	.00	.19
		11	.04	.00	.35	.05
		12	.05	.01	.00	.08
		13	.00	.01	.28	.03
		14	.03	.00	.10	.27
		15	.03	.94	.04	.00
		16	.03	.02	.20	.14

Collinearity Diagnostics[a]

Nation	Model	Dimension	Variance Proportions openness	eudaimonism	inclusive	externality	fear
		6	.00	.40	.01	.01	.08
		7	.00	.02	.20	.06	.00
		8	.01	.17	.00	.06	.02
		9	.00	.00	.03	.33	.44
		10	.01	.00	.00	.36	.05
		11	.08	.05	.00	.00	.02
		12	.22	.12	.02	.03	.00
		13	.39	.03	.00	.00	.00
		14	.07	.00	.07	.05	.00
		15	.10	.01	.00	.01	.02
		16	.12	.03	.00	.00	.00

Collinearity Diagnostics[a]

Nation	Model	Dimension	Variance Proportions transformative	fragility	valuing	inflexibility
		6	.00	.00	.00	.06
		7	.00	.02	.00	.28
		8	.38	.03	.00	.08
		9	.16	.03	.01	.00
		10	.00	.06	.02	.14
		11	.08	.03	.00	.00
		12	.13	.55	.00	.00
		13	.05	.21	.12	.00
		14	.01	.02	.75	.00
		15	.03	.00	.01	.03
		16	.02	.02	.06	.06

a. Dependent Variable: negative_affect

```
REGRESSION
 /MISSING LISTWISE
 /STATISTICS COEFF OUTS CI(95) R ANOVA COLLIN TOL CHANGE ZPP
 /CRITERIA=PIN(.05) POUT(.10)
 /NOORIGIN
 /DEPENDENT positive_affect
 /METHOD=ENTER age sex extraversion agreeable conscientiousness neuroticis
```

```
m openness
  /METHOD=ENTER eudaimonism inclusive externality fear transformative fragi
lity valuing
    inflexibility.
```

Regression

Notes

Output Created		20-MAY-2019 12:23:17
Comments		
Input	Data	C:\Users\1\Desktop\korea cnada study\merge korea canada\final.sav
	Active Dataset	DataSet1
	Filter	<none>
	Weight	<none>
	Split File	Nation
	N of Rows in Working Data File	1837
Missing Value Handling	Definition of Missing	User-defined missing values are treated as missing.
	Cases Used	Statistics are based on cases with no missing values for any variable used.
Syntax		REGRESSION /MISSING LISTWISE /STATISTICS COEFF OUTS CI(95) R ANOVA COLLIN TOL CHANGE ZPP /CRITERIA=PIN(.05) POUT(.10) /NOORIGIN /DEPENDENT positive_affect /METHOD=ENTER age sex extraversion agreeable conscientiousness neuroticism openness /METHOD=ENTER eudaimonism inclusive externality fear transformative fragility valuing inflexibility.
Resources	Processor Time	00:00:00.06
	Elapsed Time	00:00:00.04

Notes

	Memory Required	21520 bytes
	Additional Memory Required for Residual Plots	0 bytes

Variables Entered/Removed[a]

Nation	Model	Variables Entered	Variables Removed	Method
Korea	1	openness, age, sex, extraversion, neuroticism, conscientiousness, agreeable[b]	.	Enter
	2	inflexibility, transformative, inclusive, eudaimonism, fragility, valuing, fear, externality[b]	.	Enter
Canada	1	openness, sex, conscientiousness, extraversion, age, agreeable, neuroticism[b]	.	Enter
	2	transformative, inclusive, eudaimonism, inflexibility, fragility, valuing, fear, externality[b]	.	Enter

a. Dependent Variable: positive_affect

b. All requested variables entered.

Model Summary

Nation	Model	R	R Square	Adjusted R Square	Std. Error of the Estimate	Change ... R Square Change
Korea	1	.432[a]	.186	.181	.67855	.186
	2	.542[b]	.294	.285	.63413	.108
Canada	1	.678[c]	.460	.454	.63730	.460
	2	.731[d]	.534	.523	.59547	.074

Model Summary

Nation	Model	Change Statistics F Change	df1	df2	Sig. F Change
Korea	1	38.223	7	1169	.000
	2	22.187	8	1161	.000
Canada	1	79.240	7	652	.000
	2	12.853	8	644	.000

a. Predictors: (Constant), openness, age, sex, extraversion, neuroticism, conscientiousness, agreeable

b. Predictors: (Constant), openness, age, sex, extraversion, neuroticism, conscientiousness, agreeable, inflexibility, transformative, inclusive, eudaimonism, fragility, valuing, fear, externality

c. Predictors: (Constant), openness, sex, conscientiousness, extraversion, age, agreeable, neuroticism

d. Predictors: (Constant), openness, sex, conscientiousness, extraversion, age, agreeable, neuroticism, transformative, inclusive, eudaimonism, inflexibility, fragility, valuing, fear, externality

ANOVA[a]

Nation	Model		Sum of Squares	df	Mean Square	F	Sig.
Korea	1	Regression	123.193	7	17.599	38.223	.000[b]
		Residual	538.244	1169	.460		
		Total	661.438	1176			
	2	Regression	194.570	15	12.971	32.257	.000[c]
		Residual	466.868	1161	.402		
		Total	661.438	1176			
Canada	1	Regression	225.283	7	32.183	79.240	.000[d]
		Residual	264.811	652	.406		
		Total	490.093	659			
	2	Regression	261.742	15	17.449	49.211	.000[e]
		Residual	228.351	644	.355		
		Total	490.093	659			

a. Dependent Variable: positive_affect

b. Predictors: (Constant), openness, age, sex, extraversion, neuroticism, conscientiousness, agreeable

c. Predictors: (Constant), openness, age, sex, extraversion, neuroticism, conscientiousness, agreeable, inflexibility, transformative, inclusive, eudaimonism, fragility, valuing, fear, externality

d. Predictors: (Constant), openness, sex, conscientiousness, extraversion, age, agreeable, neuroticism

e. Predictors: (Constant), openness, sex, conscientiousness, extraversion, age, agreeable, neuroticism, transformative, inclusive, eudaimonism, inflexibility, fragility, valuing, fear, externality

Coefficients[a]

Nation	Model		Unstandardized Coefficients B	Unstandardized Coefficients Std. Error	Standardized Coefficients Beta	t
Korea	1	(Constant)	2.155	.210		10.271
		age	.000	.002	.006	.231
		sex	.058	.041	.039	1.416
		extraversion	.283	.032	.270	8.793
		agreeable	.042	.036	.036	1.163
		conscientiousness	.055	.030	.051	1.807
		neuroticism	-.286	.031	-.260	-9.365
		openness	.092	.031	.081	2.919
	2	(Constant)	2.823	.245		11.543
		age	-.001	.002	-.019	-.703
		sex	.025	.039	.016	.633
		extraversion	.262	.030	.250	8.656
		agreeable	-.021	.035	-.017	-.594
		conscientiousness	.047	.029	.044	1.658
		neuroticism	-.211	.031	-.192	-6.906
		openness	.086	.030	.076	2.900
		eudaimonism	-.013	.004	-.091	-3.518
		inclusive	.098	.017	.147	5.682
		externality	-.093	.021	-.132	-4.337
		fear	-.100	.021	-.144	-4.729
		transformative	.109	.019	.165	5.759
		fragility	-.092	.019	-.130	-4.850
		valuing	-.003	.029	-.003	-.099
		inflexibility	.011	.019	.016	.595
Canada	1	(Constant)	3.427	.253		13.560
		age	-.001	.002	-.026	-.844
		sex	.035	.055	.019	.634
		extraversion	.156	.027	.177	5.714
		agreeable	.116	.035	.110	3.343
		conscientiousness	.093	.032	.092	2.945
		neuroticism	-.511	.031	-.546	-16.559
		openness	-.027	.033	-.024	-.807
	2	(Constant)	3.647	.289		12.638
		age	-.001	.002	-.015	-.491
		sex	-.041	.052	-.023	-.797
		extraversion	.091	.026	.103	3.430
		agreeable	.071	.034	.067	2.074

Coefficients[a]

Nation	Model		Sig.	95.0% Confidence Interval for B Lower Bound	95.0% Confidence Interval for B Upper Bound	Correlations Zero-order
Korea	1	(Constant)	.000	1.743	2.566	
		age	.817	-.003	.004	.044
		sex	.157	-.022	.138	-.001
		extraversion	.000	.220	.346	.317
		agreeable	.245	-.029	.114	.205
		conscientiousness	.071	-.005	.114	.158
		neuroticism	.000	-.346	-.226	-.274
		openness	.004	.030	.153	.167
	2	(Constant)	.000	2.343	3.303	
		age	.482	-.004	.002	.044
		sex	.527	-.052	.101	-.001
		extraversion	.000	.203	.321	.317
		agreeable	.553	-.089	.048	.205
		conscientiousness	.098	-.009	.104	.158
		neuroticism	.000	-.271	-.151	-.274
		openness	.004	.028	.144	.167
		eudaimonism	.000	-.020	-.006	-.004
		inclusive	.000	.064	.131	.227
		externality	.000	-.135	-.051	-.300
		fear	.000	-.142	-.059	-.246
		transformative	.000	.072	.146	.085
		fragility	.000	-.129	-.055	-.193
		valuing	.921	-.060	.054	-.105
		inflexibility	.552	-.026	.048	-.078
Canada	1	(Constant)	.000	2.931	3.923	
		age	.399	-.005	.002	.188
		sex	.527	-.073	.143	-.024
		extraversion	.000	.102	.209	.359
		agreeable	.001	.048	.184	.274
		conscientiousness	.003	.031	.156	.326
		neuroticism	.000	-.572	-.451	-.630
		openness	.420	-.092	.039	.084
	2	(Constant)	.000	3.080	4.213	
		age	.623	-.004	.002	.188
		sex	.426	-.143	.061	-.024
		extraversion	.001	.039	.143	.359
		agreeable	.039	.004	.137	.274

Coefficients[a]

Nation	Model		Correlations Partial	Correlations Part	Collinearity Statistics Tolerance	Collinearity Statistics VIF
Korea	1	(Constant)				
		age	.007	.006	.920	1.086
		sex	.041	.037	.941	1.063
		extraversion	.249	.232	.739	1.354
		agreeable	.034	.031	.730	1.370
		conscientiousness	.053	.048	.876	1.142
		neuroticism	-.264	-.247	.903	1.108
		openness	.085	.077	.908	1.101
	2	(Constant)				
		age	-.021	-.017	.871	1.148
		sex	.019	.016	.908	1.101
		extraversion	.246	.213	.729	1.372
		agreeable	-.017	-.015	.702	1.424
		conscientiousness	.049	.041	.861	1.161
		neuroticism	-.199	-.170	.789	1.267
		openness	.085	.072	.894	1.118
		eudaimonism	-.103	-.087	.917	1.090
		inclusive	.164	.140	.911	1.097
		externality	-.126	-.107	.655	1.526
		fear	-.137	-.117	.656	1.526
		transformative	.167	.142	.742	1.347
		fragility	-.141	-.120	.845	1.183
		valuing	-.003	-.002	.803	1.246
		inflexibility	.017	.015	.853	1.172
Canada	1	(Constant)				
		age	-.033	-.024	.862	1.159
		sex	.025	.018	.876	1.142
		extraversion	.218	.164	.864	1.157
		agreeable	.130	.096	.772	1.296
		conscientiousness	.115	.085	.848	1.180
		neuroticism	-.544	-.477	.764	1.310
		openness	-.032	-.023	.922	1.085
	2	(Constant)				
		age	-.019	-.013	.829	1.206
		sex	-.031	-.021	.852	1.174
		extraversion	.134	.092	.801	1.249
		agreeable	.081	.056	.701	1.426

Coefficients[a]

Nation	Model		Unstandardized Coefficients B	Std. Error	Standardized Coefficients Beta	t
		conscientiousness	.085	.030	.084	2.840
		neuroticism	-.443	.032	-.472	-14.015
		openness	-.056	.032	-.050	-1.748
		eudaimonism	-.001	.004	-.009	-.327
		inclusive	.108	.019	.166	5.705
		externality	-.095	.024	-.146	-3.944
		fear	-.109	.023	-.176	-4.769
		transformative	.040	.020	.058	1.951
		fragility	-.015	.021	-.023	-.725
		valuing	.029	.029	.033	1.022
		inflexibility	.060	.022	.087	2.720

Coefficients[a]

Nation	Model		Sig.	95.0% Confidence Interval for B Lower Bound	Upper Bound	Correlations Zero-order
		conscientiousness	.005	.026	.144	.326
		neuroticism	.000	-.505	-.381	-.630
		openness	.081	-.119	.007	.084
		eudaimonism	.744	-.010	.007	.076
		inclusive	.000	.071	.146	.217
		externality	.000	-.142	-.048	-.376
		fear	.000	-.154	-.064	-.430
		transformative	.051	.000	.080	-.019
		fragility	.469	-.056	.026	-.287
		valuing	.307	-.027	.086	-.247
		inflexibility	.007	.017	.102	-.028

Coefficients[a]

Nation	Model		Correlations Partial	Part	Collinearity Statistics Tolerance	VIF
		conscientiousness	.111	.076	.826	1.211
		neuroticism	-.483	-.377	.637	1.569
		openness	-.069	-.047	.876	1.142
		eudaimonism	-.013	-.009	.904	1.106
		inclusive	.219	.153	.856	1.168
		externality	-.154	-.106	.529	1.889
		fear	-.185	-.128	.530	1.888
		transformative	.077	.052	.807	1.238
		fragility	-.029	-.019	.746	1.341
		valuing	.040	.027	.676	1.480
		inflexibility	.107	.073	.713	1.402

a. Dependent Variable: positive_affect

Excluded Variables[a]

Nation	Model		Beta In	t	Sig.	Partial Correlation	Collinearity . Tolerance
Korea	1	eudaimonism	-.058[b]	-2.146	.032	-.063	.956
		inclusive	.175[b]	6.631	.000	.190	.961
		externality	-.205[b]	-7.620	.000	-.218	.917
		fear	-.166[b]	-6.197	.000	-.178	.937
		transformative	.088[b]	3.308	.001	.096	.972
		fragility	-.138[b]	-5.199	.000	-.150	.966
		valuing	-.044[b]	-1.577	.115	-.046	.906
		inflexibility	-.043[b]	-1.620	.106	-.047	.973
Canada	1	eudaimonism	.008[c]	.272	.785	.011	.925
		inclusive	.169[c]	5.826	.000	.223	.936
		externality	-.161[c]	-5.205	.000	-.200	.832
		fear	-.185[c]	-5.866	.000	-.224	.790
		transformative	.017[c]	.584	.559	.023	.948
		fragility	-.081[c]	-2.671	.008	-.104	.889
		valuing	-.002[c]	-.051	.959	-.002	.842
		inflexibility	.004[c]	.136	.892	.005	.918

Excluded Variables[a]

Nation	Model		Collinearity Statistics VIF	Collinearity Statistics Minimum Tolerance
Korea	1	eudaimonism	1.045	.729
		inclusive	1.040	.725
		externality	1.090	.726
		fear	1.067	.725
		transformative	1.029	.723
		fragility	1.035	.730
		valuing	1.104	.730
		inflexibility	1.028	.721
Canada	1	eudaimonism	1.081	.754
		inclusive	1.068	.764
		externality	1.202	.735
		fear	1.266	.720
		transformative	1.055	.759
		fragility	1.125	.722
		valuing	1.188	.693
		inflexibility	1.089	.744

a. Dependent Variable: positive_affect

b. Predictors in the Model: (Constant), openness, age, sex, extraversion, neuroticism, conscientiousness, agreeable

c. Predictors in the Model: (Constant), openness, sex, conscientiousness, extraversion, age, agreeable,

Collinearity Diagnostics[a]

Nation	Model	Dimension	Eigenvalue	Condition Index	Variance Proportions (Constant)	age	sex
Korea	1	1	7.661	1.000	.00	.00	.00
		2	.105	8.547	.00	.22	.46
		3	.080	9.769	.00	.39	.22
		4	.057	11.572	.00	.00	.24
		5	.040	13.817	.00	.18	.02
		6	.027	16.936	.00	.08	.01
		7	.022	18.558	.00	.00	.02
		8	.007	32.008	.99	.12	.03
	2	1	15.013	1.000	.00	.00	.00
		2	.194	8.799	.00	.01	.01
		3	.132	10.681	.00	.03	.20
		4	.109	11.763	.00	.02	.02
		5	.095	12.593	.00	.12	.17
		6	.087	13.142	.00	.05	.23
		7	.075	14.174	.00	.33	.11
		8	.057	16.243	.00	.15	.02
		9	.054	16.706	.00	.06	.11
		10	.044	18.477	.00	.00	.00
		11	.041	19.092	.00	.05	.05
		12	.030	22.306	.00	.03	.00
		13	.026	23.802	.00	.09	.00
		14	.022	26.408	.00	.00	.01
		15	.017	29.438	.00	.02	.03
		16	.005	54.024	.99	.04	.04
Canada	1	1	7.555	1.000	.00	.00	.00
		2	.149	7.123	.00	.05	.04
		3	.095	8.928	.00	.34	.06
		4	.070	10.412	.00	.01	.51
		5	.055	11.677	.00	.34	.16
		6	.040	13.690	.00	.04	.01
		7	.028	16.433	.00	.03	.16
		8	.008	30.436	1.00	.20	.05
	2	1	14.609	1.000	.00	.00	.00
		2	.396	6.072	.00	.00	.00
		3	.173	9.178	.00	.04	.02
		4	.131	10.545	.00	.07	.00
		5	.107	11.702	.00	.00	.11

Collinearity Diagnostics[a]

Nation	Model	Dimension	Variance Proportions extraversion	agreeable	conscientiousness	neuroticism
Korea	1	1	.00	.00	.00	.00
		2	.00	.00	.02	.04
		3	.13	.03	.00	.00
		4	.09	.02	.00	.56
		5	.32	.01	.20	.08
		6	.00	.00	.56	.02
		7	.46	.83	.05	.01
		8	.00	.11	.17	.30
	2	1	.00	.00	.00	.00
		2	.01	.00	.00	.00
		3	.01	.01	.00	.02
		4	.00	.00	.01	.00
		5	.00	.00	.01	.01
		6	.06	.02	.00	.00
		7	.04	.00	.00	.00
		8	.12	.01	.00	.02
		9	.05	.00	.00	.17
		10	.01	.01	.01	.24
		11	.14	.00	.14	.19
		12	.09	.00	.15	.04
		13	.01	.00	.49	.01
		14	.46	.81	.03	.00
		15	.00	.04	.07	.21
		16	.00	.08	.09	.07
Canada	1	1	.00	.00	.00	.00
		2	.16	.00	.01	.25
		3	.45	.00	.02	.00
		4	.21	.02	.02	.26
		5	.11	.01	.16	.02
		6	.03	.03	.54	.06
		7	.02	.92	.02	.02
		8	.03	.02	.22	.40
	2	1	.00	.00	.00	.00
		2	.03	.00	.00	.00
		3	.02	.00	.00	.05
		4	.01	.00	.02	.00
		5	.02	.00	.00	.10

Collinearity Diagnostics[a]

Nation	Model	Dimension	Variance Proportions openness	eudaimonism	inclusive	externality	fear
Korea	1	1	.00				
		2	.00				
		3	.02				
		4	.00				
		5	.22				
		6	.58				
		7	.03				
		8	.14				
	2	1	.00	.00	.00	.00	.00
		2	.00	.04	.07	.10	.14
		3	.00	.14	.08	.01	.09
		4	.00	.21	.68	.00	.01
		5	.00	.12	.00	.02	.07
		6	.01	.22	.01	.07	.03
		7	.01	.20	.06	.10	.00
		8	.00	.01	.00	.03	.23
		9	.00	.00	.02	.10	.11
		10	.02	.00	.00	.49	.22
		11	.14	.01	.00	.00	.07
		12	.11	.01	.02	.02	.00
		13	.58	.00	.00	.03	.01
		14	.03	.00	.01	.01	.01
		15	.00	.00	.02	.01	.01
		16	.08	.03	.01	.00	.00
Canada	1	1	.00				
		2	.00				
		3	.00				
		4	.00				
		5	.23				
		6	.40				
		7	.18				
		8	.17				
	2	1	.00	.00	.00	.00	.00
		2	.00	.01	.01	.04	.12
		3	.00	.01	.01	.01	.01
		4	.00	.01	.65	.00	.00
		5	.00	.15	.00	.04	.24

Collinearity Diagnostics[a]

Nation	Model	Dimension	Variance Proportions transformative	fragility	valuing	inflexibility
Korea	1	1				
		2				
		3				
		4				
		5				
		6				
		7				
		8				
	2	1	.00	.00	.00	.00
		2	.00	.00	.00	.01
		3	.03	.00	.00	.00
		4	.00	.00	.00	.00
		5	.05	.00	.00	.16
		6	.06	.01	.00	.03
		7	.10	.01	.00	.00
		8	.12	.08	.00	.22
		9	.02	.04	.01	.39
		10	.25	.01	.00	.05
		11	.12	.10	.00	.03
		12	.19	.63	.00	.01
		13	.03	.00	.02	.00
		14	.02	.02	.01	.00
		15	.00	.03	.84	.02
		16	.00	.05	.11	.07
Canada	1	1				
		2				
		3				
		4				
		5				
		6				
		7				
		8				
	2	1	.00	.00	.00	.00
		2	.00	.00	.00	.02
		3	.03	.01	.00	.31
		4	.00	.01	.00	.00
		5	.10	.00	.01	.01

Collinearity Diagnostics[a]

Nation	Model	Dimension	Eigenvalue	Condition Index	Variance Proportions (Constant)	age	sex
		6	.097	12.289	.00	.00	.00
		7	.086	13.039	.00	.21	.09
		8	.073	14.174	.00	.01	.07
		9	.070	14.491	.00	.10	.10
		10	.061	15.481	.00	.00	.33
		11	.051	16.871	.00	.35	.02
		12	.044	18.168	.00	.00	.03
		13	.038	19.549	.00	.00	.01
		14	.031	21.685	.00	.01	.03
		15	.026	23.599	.00	.05	.12
		16	.006	50.079	1.00	.15	.06

Collinearity Diagnostics[a]

Nation	Model	Dimension	Variance Proportions extraversion	agreeable	conscientiousness	neuroticism
		6	.38	.00	.00	.00
		7	.13	.00	.01	.01
		8	.17	.00	.00	.06
		9	.06	.00	.00	.01
		10	.01	.01	.00	.19
		11	.04	.00	.35	.05
		12	.05	.01	.00	.08
		13	.00	.01	.28	.03
		14	.03	.00	.10	.27
		15	.03	.94	.04	.00
		16	.03	.02	.20	.14

Collinearity Diagnostics[a]

Nation	Model	Dimension	Variance Proportions openness	eudaimonism	inclusive	externality	fear
		6	.00	.40	.01	.01	.08
		7	.00	.02	.20	.06	.00
		8	.01	.17	.00	.06	.02
		9	.00	.00	.03	.33	.44
		10	.01	.00	.00	.36	.05
		11	.08	.05	.00	.00	.02
		12	.22	.12	.02	.03	.00
		13	.39	.03	.00	.00	.00
		14	.07	.00	.07	.05	.00
		15	.10	.01	.00	.01	.02
		16	.12	.03	.00	.00	.00

Collinearity Diagnostics[a]

Nation	Model	Dimension	Variance Proportions transformative	fragility	valuing	inflexibility
		6	.00	.00	.00	.06
		7	.00	.02	.00	.28
		8	.38	.03	.00	.08
		9	.16	.03	.01	.00
		10	.00	.06	.02	.14
		11	.08	.03	.00	.00
		12	.13	.55	.00	.00
		13	.05	.21	.12	.00
		14	.01	.02	.75	.00
		15	.03	.00	.01	.03
		16	.02	.02	.06	.06

a. Dependent Variable: positive_affect

```
REGRESSION
 /MISSING LISTWISE
 /STATISTICS COEFF OUTS CI(95) R ANOVA COLLIN TOL CHANGE ZPP
 /CRITERIA=PIN(.05) POUT(.10)
 /NOORIGIN
 /DEPENDENT materialism
 /METHOD=ENTER age sex extraversion agreeable conscientiousness neuroticis
```

```
m openness
  /METHOD=ENTER eudaimonism inclusive externality fear transformative fragi
lity valuing
    inflexibility.
```

Regression

Notes

Output Created		20-MAY-2019 12:23:46
Comments		
Input	Data	C:\Users\1\Desktop\korea cnada study\merge korea canada\final.sav
	Active Dataset	DataSet1
	Filter	<none>
	Weight	<none>
	Split File	Nation
	N of Rows in Working Data File	1837
Missing Value Handling	Definition of Missing	User-defined missing values are treated as missing.
	Cases Used	Statistics are based on cases with no missing values for any variable used.
Syntax		REGRESSION<br>/MISSING LISTWISE<br>/STATISTICS COEFF OUTS CI(95) R ANOVA COLLIN TOL CHANGE ZPP<br>/CRITERIA=PIN(.05) POUT(.10)<br>/NOORIGIN<br>/DEPENDENT materialism<br>/METHOD=ENTER age sex extraversion agreeable conscientiousness neuroticism openness<br>/METHOD=ENTER eudaimonism inclusive externality fear transformative fragility valuing<br>inflexibility.
Resources	Processor Time	00:00:00.02
	Elapsed Time	00:00:00.04

Notes

	Memory Required	21520 bytes
	Additional Memory Required for Residual Plots	0 bytes

Variables Entered/Removed[a]

Nation	Model	Variables Entered	Variables Removed	Method
Korea	1	openness, age, sex, extraversion, neuroticism, conscientiousness, agreeable[b]	.	Enter
	2	inflexibility, transformative, inclusive, eudaimonism, fragility, valuing, fear, externality[b]	.	Enter
Canada	1	openness, sex, conscientiousness, extraversion, age, agreeable, neuroticism[b]	.	Enter
	2	transformative, inclusive, eudaimonism, inflexibility, fragility, valuing, fear, externality[b]	.	Enter

a. Dependent Variable: materialism

b. All requested variables entered.

Model Summary

Nation	Model	R	R Square	Adjusted R Square	Std. Error of the Estimate	Change ... R Square Change
Korea	1	.337[a]	.114	.108	.48796	.114
	2	.546[b]	.298	.289	.43590	.184
Canada	1	.395[c]	.156	.147	.51570	.156
	2	.530[d]	.280	.264	.47915	.124

Model Summary

Nation	Model	Change Statistics F Change	df1	df2	Sig. F Change
Korea	1	21.446	7	1169	.000
	2	37.992	8	1161	.000
Canada	1	17.241	7	652	.000
	2	13.907	8	644	.000

a. Predictors: (Constant), openness, age, sex, extraversion, neuroticism, conscientiousness, agreeable

b. Predictors: (Constant), openness, age, sex, extraversion, neuroticism, conscientiousness, agreeable, inflexibility, transformative, inclusive, eudaimonism, fragility, valuing, fear, externality

c. Predictors: (Constant), openness, sex, conscientiousness, extraversion, age, agreeable, neuroticism

d. Predictors: (Constant), openness, sex, conscientiousness, extraversion, age, agreeable, neuroticism, transformative, inclusive, eudaimonism, inflexibility, fragility, valuing, fear, externality

ANOVA[a]

Nation	Model		Sum of Squares	df	Mean Square	F	Sig.
Korea	1	Regression	35.745	7	5.106	21.446	.000[b]
		Residual	278.348	1169	.238		
		Total	314.093	1176			
	2	Regression	93.494	15	6.233	32.804	.000[c]
		Residual	220.598	1161	.190		
		Total	314.093	1176			
Canada	1	Regression	32.096	7	4.585	17.241	.000[d]
		Residual	173.399	652	.266		
		Total	205.495	659			
	2	Regression	57.640	15	3.843	16.737	.000[e]
		Residual	147.855	644	.230		
		Total	205.495	659			

a. Dependent Variable: materialism

b. Predictors: (Constant), openness, age, sex, extraversion, neuroticism, conscientiousness, agreeable

c. Predictors: (Constant), openness, age, sex, extraversion, neuroticism, conscientiousness, agreeable, inflexibility, transformative, inclusive, eudaimonism, fragility, valuing, fear, externality

d. Predictors: (Constant), openness, sex, conscientiousness, extraversion, age, agreeable, neuroticism

e. Predictors: (Constant), openness, sex, conscientiousness, extraversion, age, agreeable, neuroticism, transformative, inclusive, eudaimonism, inflexibility, fragility, valuing, fear, externality

Coefficients[a]

Nation	Model		Unstandardized Coefficients B	Unstandardized Coefficients Std. Error	Standardized Coefficients Beta	t
Korea	1	(Constant)	3.126	.151		20.723
		age	-.010	.001	-.229	-7.971
		sex	-.019	.029	-.018	-.640
		extraversion	.032	.023	.045	1.402
		agreeable	-.005	.026	-.007	-.210
		conscientiousness	.048	.022	.065	2.214
		neuroticism	.171	.022	.226	7.798
		openness	-.025	.023	-.031	-1.089
	2	(Constant)	2.105	.168		12.520
		age	-.010	.001	-.236	-8.943
		sex	.023	.027	.023	.878
		extraversion	.038	.021	.053	1.832
		agreeable	-.002	.024	-.003	-.104
		conscientiousness	.037	.020	.050	1.871
		neuroticism	.060	.021	.079	2.845
		openness	-.008	.020	-.010	-.400
		eudaimonism	-.011	.003	-.111	-4.313
		inclusive	-.005	.012	-.010	-.381
		externality	.077	.015	.158	5.194
		fear	-.037	.015	-.077	-2.545
		transformative	-.024	.013	-.054	-1.887
		fragility	.023	.013	.047	1.760
		valuing	.267	.020	.369	13.433
		inflexibility	.008	.013	.017	.621
Canada	1	(Constant)	3.247	.205		15.878
		age	-.011	.001	-.301	-7.772
		sex	-.023	.044	-.020	-.523
		extraversion	.037	.022	.066	1.699
		agreeable	-.077	.028	-.112	-2.745
		conscientiousness	-.011	.026	-.017	-.434
		neuroticism	.072	.025	.118	2.862
		openness	-.055	.027	-.076	-2.035
	2	(Constant)	2.205	.232		9.498
		age	-.010	.001	-.291	-7.933
		sex	.012	.042	.010	.284
		extraversion	.039	.021	.068	1.820
		agreeable	-.051	.027	-.074	-1.862

Coefficients[a]

Nation	Model		Sig.	95.0% Confidence Interval for B Lower Bound	Upper Bound	Correlations Zero-order
Korea	1	(Constant)	.000	2.830	3.422	
		age	.000	-.012	-.007	-.253
		sex	.523	-.076	.039	.040
		extraversion	.161	-.013	.078	.047
		agreeable	.834	-.057	.046	.023
		conscientiousness	.027	.006	.091	-.032
		neuroticism	.000	.128	.214	.250
		openness	.276	-.069	.020	-.011
	2	(Constant)	.000	1.775	2.435	
		age	.000	-.012	-.008	-.253
		sex	.380	-.029	.076	.040
		extraversion	.067	-.003	.079	.047
		agreeable	.917	-.049	.044	.023
		conscientiousness	.062	-.002	.075	-.032
		neuroticism	.005	.019	.101	.250
		openness	.690	-.048	.032	-.011
		eudaimonism	.000	-.016	-.006	-.198
		inclusive	.703	-.028	.019	-.057
		externality	.000	.048	.106	.270
		fear	.011	-.066	-.009	.082
		transformative	.059	-.050	.001	-.034
		fragility	.079	-.003	.049	.131
		valuing	.000	.228	.306	.402
		inflexibility	.535	-.017	.033	.092
Canada	1	(Constant)	.000	2.846	3.649	
		age	.000	-.013	-.008	-.343
		sex	.601	-.110	.064	.023
		extraversion	.090	-.006	.081	-.041
		agreeable	.006	-.132	-.022	-.182
		conscientiousness	.664	-.062	.039	-.131
		neuroticism	.004	.022	.121	.223
		openness	.042	-.108	-.002	-.092
	2	(Constant)	.000	1.749	2.661	
		age	.000	-.013	-.008	-.343
		sex	.776	-.070	.094	.023
		extraversion	.069	-.003	.081	-.041
		agreeable	.063	-.105	.003	-.182

Coefficients[a]

Nation	Model		Correlations Partial	Correlations Part	Collinearity Statistics Tolerance	Collinearity Statistics VIF
Korea	1	(Constant)				
		age	-.227	-.219	.920	1.086
		sex	-.019	-.018	.941	1.063
		extraversion	.041	.039	.739	1.354
		agreeable	-.006	-.006	.730	1.370
		conscientiousness	.065	.061	.876	1.142
		neuroticism	.222	.215	.903	1.108
		openness	-.032	-.030	.908	1.101
	2	(Constant)				
		age	-.254	-.220	.871	1.148
		sex	.026	.022	.908	1.101
		extraversion	.054	.045	.729	1.372
		agreeable	-.003	-.003	.702	1.424
		conscientiousness	.055	.046	.861	1.161
		neuroticism	.083	.070	.789	1.267
		openness	-.012	-.010	.894	1.118
		eudaimonism	-.126	-.106	.917	1.090
		inclusive	-.011	-.009	.911	1.097
		externality	.151	.128	.655	1.526
		fear	-.074	-.063	.656	1.526
		transformative	-.055	-.046	.742	1.347
		fragility	.052	.043	.845	1.183
		valuing	.367	.330	.803	1.246
		inflexibility	.018	.015	.853	1.172
Canada	1	(Constant)				
		age	-.291	-.280	.862	1.159
		sex	-.020	-.019	.876	1.142
		extraversion	.066	.061	.864	1.157
		agreeable	-.107	-.099	.772	1.296
		conscientiousness	-.017	-.016	.848	1.180
		neuroticism	.111	.103	.764	1.310
		openness	-.079	-.073	.922	1.085
	2	(Constant)				
		age	-.298	-.265	.829	1.206
		sex	.011	.009	.852	1.174
		extraversion	.072	.061	.801	1.249
		agreeable	-.073	-.062	.701	1.426

Coefficients[a]

Nation	Model		Unstandardized Coefficients B	Unstandardized Coefficients Std. Error	Standardized Coefficients Beta	t
		conscientiousness	.009	.024	.014	.379
		neuroticism	-.006	.025	-.009	-.222
		openness	-.027	.026	-.038	-1.051
		eudaimonism	-.007	.004	-.072	-2.051
		inclusive	.010	.015	.023	.642
		externality	.030	.019	.072	1.566
		fear	-.004	.018	-.010	-.225
		transformative	.007	.016	.015	.400
		fragility	.027	.017	.062	1.614
		valuing	.166	.023	.292	7.187
		inflexibility	.027	.018	.061	1.554

Coefficients[a]

Nation	Model		Sig.	95.0% Confidence Interval for B Lower Bound	95.0% Confidence Interval for B Upper Bound	Correlations Zero-order
		conscientiousness	.705	-.038	.057	-.131
		neuroticism	.824	-.056	.044	.223
		openness	.294	-.078	.023	-.092
		eudaimonism	.041	-.015	.000	-.120
		inclusive	.521	-.020	.040	.080
		externality	.118	-.008	.068	.251
		fear	.822	-.040	.032	.233
		transformative	.689	-.026	.039	.110
		fragility	.107	-.006	.059	.199
		valuing	.000	.121	.211	.414
		inflexibility	.121	-.007	.062	.143

Coefficients[a]

Nation	Model		Correlations Partial	Part	Collinearity Statistics Tolerance	VIF
		conscientiousness	.015	.013	.826	1.211
		neuroticism	-.009	-.007	.637	1.569
		openness	-.041	-.035	.876	1.142
		eudaimonism	-.081	-.069	.904	1.106
		inclusive	.025	.021	.856	1.168
		externality	.062	.052	.529	1.889
		fear	-.009	-.008	.530	1.888
		transformative	.016	.013	.807	1.238
		fragility	.063	.054	.746	1.341
		valuing	.273	.240	.676	1.480
		inflexibility	.061	.052	.713	1.402

a. Dependent Variable: materialism

Excluded Variables[a]

Nation	Model		Beta In	t	Sig.	Partial Correlation	Collinearity . Tolerance
Korea	1	eudaimonism	-.150[b]	-5.380	.000	-.156	.956
		inclusive	-.012[b]	-.436	.663	-.013	.961
		externality	.232[b]	8.312	.000	.236	.917
		fear	.061[b]	2.130	.033	.062	.937
		transformative	-.022[b]	-.800	.424	-.023	.972
		fragility	.091[b]	3.279	.001	.096	.966
		valuing	.393[b]	14.821	.000	.398	.906
		inflexibility	.115[b]	4.140	.000	.120	.973
Canada	1	eudaimonism	-.089[c]	-2.389	.017	-.093	.925
		inclusive	.100[c]	2.709	.007	.106	.936
		externality	.220[c]	5.707	.000	.218	.832
		fear	.156[c]	3.897	.000	.151	.790
		transformative	.072[c]	1.953	.051	.076	.948
		fragility	.150[c]	3.964	.000	.154	.889
		valuing	.352[c]	9.588	.000	.352	.842
		inflexibility	.147[c]	3.971	.000	.154	.918

Excluded Variables[a]

Nation	Model		Collinearity Statistics VIF	Collinearity Statistics Minimum Tolerance
Korea	1	eudaimonism	1.045	.729
		inclusive	1.040	.725
		externality	1.090	.726
		fear	1.067	.725
		transformative	1.029	.723
		fragility	1.035	.730
		valuing	1.104	.730
		inflexibility	1.028	.721
Canada	1	eudaimonism	1.081	.754
		inclusive	1.068	.764
		externality	1.202	.735
		fear	1.266	.720
		transformative	1.055	.759
		fragility	1.125	.722
		valuing	1.188	.693
		inflexibility	1.089	.744

a. Dependent Variable: materialism

b. Predictors in the Model: (Constant), openness, age, sex, extraversion, neuroticism, conscientiousness, agreeable

c. Predictors in the Model: (Constant), openness, sex, conscientiousness, extraversion, age, agreeable,

Collinearity Diagnostics[a]

Nation	Model	Dimension	Eigenvalue	Condition Index	Variance Proportions (Constant)	age	sex
Korea	1	1	7.661	1.000	.00	.00	.00
		2	.105	8.547	.00	.22	.46
		3	.080	9.769	.00	.39	.22
		4	.057	11.572	.00	.00	.24
		5	.040	13.817	.00	.18	.02
		6	.027	16.936	.00	.08	.01
		7	.022	18.558	.00	.00	.02
		8	.007	32.008	.99	.12	.03
	2	1	15.013	1.000	.00	.00	.00
		2	.194	8.799	.00	.01	.01
		3	.132	10.681	.00	.03	.20
		4	.109	11.763	.00	.02	.02
		5	.095	12.593	.00	.12	.17
		6	.087	13.142	.00	.05	.23
		7	.075	14.174	.00	.33	.11
		8	.057	16.243	.00	.15	.02
		9	.054	16.706	.00	.06	.11
		10	.044	18.477	.00	.00	.00
		11	.041	19.092	.00	.05	.05
		12	.030	22.306	.00	.03	.00
		13	.026	23.802	.00	.09	.00
		14	.022	26.408	.00	.00	.01
		15	.017	29.438	.00	.02	.03
		16	.005	54.024	.99	.04	.04
Canada	1	1	7.555	1.000	.00	.00	.00
		2	.149	7.123	.00	.05	.04
		3	.095	8.928	.00	.34	.06
		4	.070	10.412	.00	.01	.51
		5	.055	11.677	.00	.34	.16
		6	.040	13.690	.00	.04	.01
		7	.028	16.433	.00	.03	.16
		8	.008	30.436	1.00	.20	.05
	2	1	14.609	1.000	.00	.00	.00
		2	.396	6.072	.00	.00	.00
		3	.173	9.178	.00	.04	.02
		4	.131	10.545	.00	.07	.00
		5	.107	11.702	.00	.00	.11

Collinearity Diagnostics[a]

Nation	Model	Dimension	Variance Proportions extraversion	agreeable	conscientiousness	neuroticism
Korea	1	1	.00	.00	.00	.00
		2	.00	.00	.02	.04
		3	.13	.03	.00	.00
		4	.09	.02	.00	.56
		5	.32	.01	.20	.08
		6	.00	.00	.56	.02
		7	.46	.83	.05	.01
		8	.00	.11	.17	.30
	2	1	.00	.00	.00	.00
		2	.01	.00	.00	.00
		3	.01	.01	.00	.02
		4	.00	.00	.01	.00
		5	.00	.00	.01	.01
		6	.06	.02	.00	.00
		7	.04	.00	.00	.00
		8	.12	.01	.00	.02
		9	.05	.00	.00	.17
		10	.01	.01	.01	.24
		11	.14	.00	.14	.19
		12	.09	.00	.15	.04
		13	.01	.00	.49	.01
		14	.46	.81	.03	.00
		15	.00	.04	.07	.21
		16	.00	.08	.09	.07
Canada	1	1	.00	.00	.00	.00
		2	.16	.00	.01	.25
		3	.45	.00	.02	.00
		4	.21	.02	.02	.26
		5	.11	.01	.16	.02
		6	.03	.03	.54	.06
		7	.02	.92	.02	.02
		8	.03	.02	.22	.40
	2	1	.00	.00	.00	.00
		2	.03	.00	.00	.00
		3	.02	.00	.00	.05
		4	.01	.00	.02	.00
		5	.02	.00	.00	.10

Collinearity Diagnostics[a]

Nation	Model	Dimension	Variance Proportions openness	eudaimonism	inclusive	externality	fear
Korea	1	1	.00				
		2	.00				
		3	.02				
		4	.00				
		5	.22				
		6	.58				
		7	.03				
		8	.14				
	2	1	.00	.00	.00	.00	.00
		2	.00	.04	.07	.10	.14
		3	.00	.14	.08	.01	.09
		4	.00	.21	.68	.00	.01
		5	.00	.12	.00	.02	.07
		6	.01	.22	.01	.07	.03
		7	.01	.20	.06	.10	.00
		8	.00	.01	.00	.03	.23
		9	.00	.00	.02	.10	.11
		10	.02	.00	.00	.49	.22
		11	.14	.01	.00	.00	.07
		12	.11	.01	.02	.02	.00
		13	.58	.00	.00	.03	.01
		14	.03	.00	.01	.01	.01
		15	.00	.00	.02	.01	.01
		16	.08	.03	.01	.00	.00
Canada	1	1	.00				
		2	.00				
		3	.00				
		4	.00				
		5	.23				
		6	.40				
		7	.18				
		8	.17				
	2	1	.00	.00	.00	.00	.00
		2	.00	.01	.01	.04	.12
		3	.00	.01	.01	.01	.01
		4	.00	.01	.65	.00	.00
		5	.00	.15	.00	.04	.24

Collinearity Diagnostics[a]

Nation	Model	Dimension	Variance Proportions: transformative	fragility	valuing	inflexibility
Korea	1	1				
		2				
		3				
		4				
		5				
		6				
		7				
		8				
	2	1	.00	.00	.00	.00
		2	.00	.00	.00	.01
		3	.03	.00	.00	.00
		4	.00	.00	.00	.00
		5	.05	.00	.00	.16
		6	.06	.01	.00	.03
		7	.10	.01	.00	.00
		8	.12	.08	.00	.22
		9	.02	.04	.01	.39
		10	.25	.01	.00	.05
		11	.12	.10	.00	.03
		12	.19	.63	.00	.01
		13	.03	.00	.02	.00
		14	.02	.02	.01	.00
		15	.00	.03	.84	.02
		16	.00	.05	.11	.07
Canada	1	1				
		2				
		3				
		4				
		5				
		6				
		7				
		8				
	2	1	.00	.00	.00	.00
		2	.00	.00	.00	.02
		3	.03	.01	.00	.31
		4	.00	.01	.00	.00
		5	.10	.00	.01	.01

Collinearity Diagnostics[a]

Nation	Model	Dimension	Eigenvalue	Condition Index	Variance Proportions (Constant)	age	sex
		6	.097	12.289	.00	.00	.00
		7	.086	13.039	.00	.21	.09
		8	.073	14.174	.00	.01	.07
		9	.070	14.491	.00	.10	.10
		10	.061	15.481	.00	.00	.33
		11	.051	16.871	.00	.35	.02
		12	.044	18.168	.00	.00	.03
		13	.038	19.549	.00	.00	.01
		14	.031	21.685	.00	.01	.03
		15	.026	23.599	.00	.05	.12
		16	.006	50.079	1.00	.15	.06

Collinearity Diagnostics[a]

Nation	Model	Dimension	Variance Proportions extraversion	agreeable	conscientiousn ess	neuroticism
		6	.38	.00	.00	.00
		7	.13	.00	.01	.01
		8	.17	.00	.00	.06
		9	.06	.00	.00	.01
		10	.01	.01	.00	.19
		11	.04	.00	.35	.05
		12	.05	.01	.00	.08
		13	.00	.01	.28	.03
		14	.03	.00	.10	.27
		15	.03	.94	.04	.00
		16	.03	.02	.20	.14

Collinearity Diagnostics[a]

Nation	Model	Dimension	Variance Proportions openness	eudaimonism	inclusive	externality	fear
		6	.00	.40	.01	.01	.08
		7	.00	.02	.20	.06	.00
		8	.01	.17	.00	.06	.02
		9	.00	.00	.03	.33	.44
		10	.01	.00	.00	.36	.05
		11	.08	.05	.00	.00	.02
		12	.22	.12	.02	.03	.00
		13	.39	.03	.00	.00	.00
		14	.07	.00	.07	.05	.00
		15	.10	.01	.00	.01	.02
		16	.12	.03	.00	.00	.00

Collinearity Diagnostics[a]

Nation	Model	Dimension	Variance Proportions transformative	fragility	valuing	inflexibility
		6	.00	.00	.00	.06
		7	.00	.02	.00	.28
		8	.38	.03	.00	.08
		9	.16	.03	.01	.00
		10	.00	.06	.02	.14
		11	.08	.03	.00	.00
		12	.13	.55	.00	.00
		13	.05	.21	.12	.00
		14	.01	.02	.75	.00
		15	.03	.00	.01	.03
		16	.02	.02	.06	.06

a. Dependent Variable: materialism
